# Effect of Adiponectin Variant on Lipid Profile and Plasma Adiponectin Levels: A Multicenter Systematic Review and Meta-Analysis

**DOI:** 10.1155/2022/4395266

**Published:** 2022-07-07

**Authors:** Guiqing Wang, Yufeng Wang, Zhi Luo

**Affiliations:** ^1^Department of Cardiology, The First People's Hospital of Ziyang, Ziyang, China; ^2^Department of Digestion, The First People's Hospital of Ziyang, Ziyang, China; ^3^Department of Cardiology, Zhongnan Hospital of Wuhan University, Wuhan University, Wuhan, China

## Abstract

**Background:**

Adiponectin is a recognized antiatherogenic molecule; this study was aimed at clarifying the effects of adiponectin variants on lipid and adiponectin levels.

**Methods:**

By searching PubMed and Cochrane databases for studies published before March 31, 2022, a total of 86,610 individuals were included in the analysis.

**Results:**

Variants of rs2241766 and rs266729 were associated with decreased adiponectin and high-density lipoprotein cholesterol (HDL-C), as well as increased triglycerides (TG), total cholesterol (TC), and low-density lipoprotein cholesterol (LDL-C) levels. In contrast, the rs1501299 variant was correlated with increased adiponectin and HDL-C, as well as decreased TG, TC, and LDL-C levels. Subgroup analysis indicated that the significant effect of the rs2241766 and rs266729 variants on lipid profile was predominant in Chinese, while the significant effect of the rs1501299 variant on lipid profile was primarily in Caucasians. Moreover, a stronger effect of the rs2241766 and rs1501299 variants on LDL-C levels was observed in males, while a considerable effect of the rs266729 variant on LDL-C levels was observed in children.

**Conclusions:**

The present study indicated that Chinese with the rs2241766 and rs266729 variants were at high risk of dyslipidemia, atherosclerosis, or coronary artery disease (CAD). Males with the rs2241766 variant were at high risk of CAD. Children with the rs266729 variant had a high risk to develop dyslipidemia, atherosclerosis, and even early onset of CAD in the future. These findings are beneficial to clinical physicians to choose different management strategies for cardiovascular disease (CVD) prevention.

## 1. Introduction

Adiponectin is a lipid regulator produced by white adipocytes [1]. The high and low levels of adiponectin may induce antiatherosclerotic [2] and atherogenic [3] lipid profiles, respectively. Consistent with this, the increase and decrease in adiponectin levels were proved to have antiatherosclerotic [4] and atherogenic [5] effects, respectively. Therefore, adiponectin may act as a key bridge to link lipid metabolism and atherosclerosis [6].

Dyslipidemia is characterized by increased levels of plasma triglycerides (TG), total cholesterol (TC), and low-density lipoprotein cholesterol (LDL-C) and/or decreased levels of high-density lipoprotein cholesterol (HDL-C) in plasma. Dyslipidemia may result in a variety of severe diseases in humans. For instance, dyslipidemia involving coronary arteries may induce CAD or acute myocardial infarction [7]. Moreover, dyslipidemia involving cerebrovascular vessels may cause acute ischemic stroke [8]. Notably, emerging shreds of evidence have indicated that dyslipidemia may be related to pregnancy-induced hypertension (PIH) [9] and may play an important role in cancer proliferation and metastasis [10].

The adiponectin genes (known as ADIPOQ, APM1, APN, ACDC, and ACRP30) are located in the long arm of human chromosome 3 at q27, composed of three exons and two introns. rs2241766 is located in the 2nd exon, generated by a nucleotide variation from thymine (T) to guanine (G); rs1501299 is located in the 2nd intron, generated by a nucleotide variation from guanine (G) to adenine (A); and rs266729 is located in the promoter region, generated by a nucleotide variation from cytosine (C) to guanine (G). Heid et al. [11] revealed that adiponectin levels are primarily determined by adiponectin expression. Therefore, variants of rs2241766, rs1501299, and rs266729 may affect circulating adiponectin levels by modulating adiponectin expression [12–14].

Recently, a series of animal trials [15–17] showed that adiponectin knockout caused severe dyslipidemia. Moreover, several meta-analyses indicated that variants of rs2241766, rs266729, and rs1501299 impacted CAD risk [18, 19]. Since dyslipidemia accounts for more than 50% of the population-attributable risk for the onset of CAD, indicating the remodeled CAD risk induced by adiponectin variants may originate from a remodeled lipid profile. Therefore, we conducted this study to investigate the effects of adiponectin variants on lipid metabolism under evidence-based medicine.

## 2. Material and Methods

### 2.1. Literature Search

The search of the literature was executed using PubMed and the Cochrane databases from January 1, 2021, to March 31, 2022, by entering the following keywords: (“Adiponectin”, “ADIPOQ”, “APM1”, “APN”, “ACDC”, or “ACRP30”), (“rs2241766”, “rs1501299”, “rs266729”, “+45T>G”, “T45G”, “T94G”, “Gly15Gly”, “+276G>T”, “G276T”, or “-11377C>G”), (“variant”, “mutant”, or “polymorphism”) and (“lipid”, “lipids”, “lipid metabolism”, “lipoprotein”, “cholesterol”, “blood lipid”, “serum lipid”, or “circulating lipid”).

### 2.2. Inclusion Criteria

The procedure for obtaining literature was hierarchical. The titles were first assessed, and the abstracts and contents were then checked. The detailed inclusion criteria include the following: (1) the studies detected the effects of rs2241766, rs1501299, and rs266729 on adiponectin or lipid levels. (2) The studies at least offered one lipid parameter or adiponectin levels by the genotype of rs2241766, rs1501299, and rs266729. (3) The studies provided adiponectin or lipid levels by the mean and standard deviation (SD). (4) The studies provided the genotype frequencies of rs2241766, rs1501299, and rs266729. (5) The language was limited to English and Chinese.

### 2.3. Subgroup Analysis

Subgroup analysis was executed in ethnicity, gender, and disease status. The ethnicity was divided into Chinese, Japanese, Korean, Caucasian, Latino, Indian, Middle Eastern, and other ethnicities. Disease status was divided into CAD, T2DM, hypertension, obesity, polycystic ovarian syndrome (PCOS), metabolic syndrome (Mets), and nonalcoholic fatty liver disease (NAFLD). In addition, healthy subjects, pregnant subjects, and children subjects were also isolated for analysis.

### 2.4. Other Items

Data screening between the authors was compared by kappa statistics [20], since data extraction and analysis, heterogeneity processing, and publication bias tests were adopted from the previous methods, to avoid redundant descriptions (please see Liu et al. [21] publication for more details).

## 3. Results

### 3.1. Study Selection

**Figure 1:**
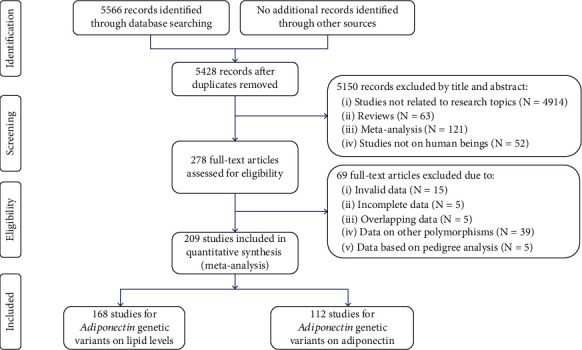


The kappa value was 0.93 (>0.75) between the authors; the details of the study selection were summarized in Figure 1 (please see Figure S1 for the full electronic search strategy).

### 3.2. Effect of rs2241766 on Lipid Profile

**Figure 2:**
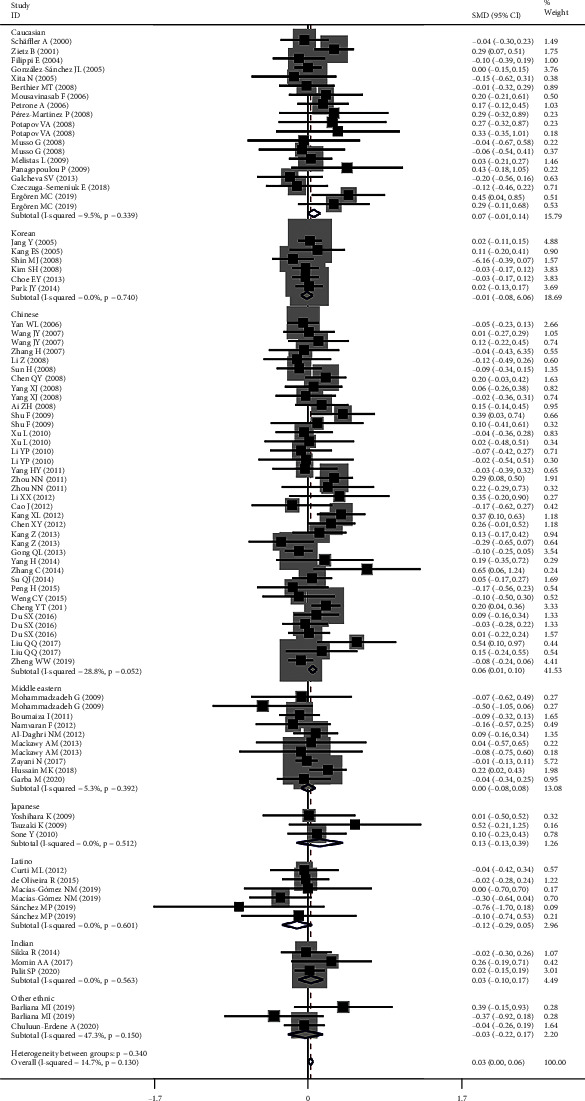


All the results stated below were the data excluding heterogeneity. rs2241766 had a harmful effect on lipid profile (Figure S2–S4 and Figure 2). Subgroup analysis indicated that the significant effect of rs2241766 on lipid profile was primarily in Chinese, males, CAD patients, and T2DM patients (please see Table 1 for more details).

### 3.3. Effect of rs1501299 on Lipid Profile

**Figure 3:**
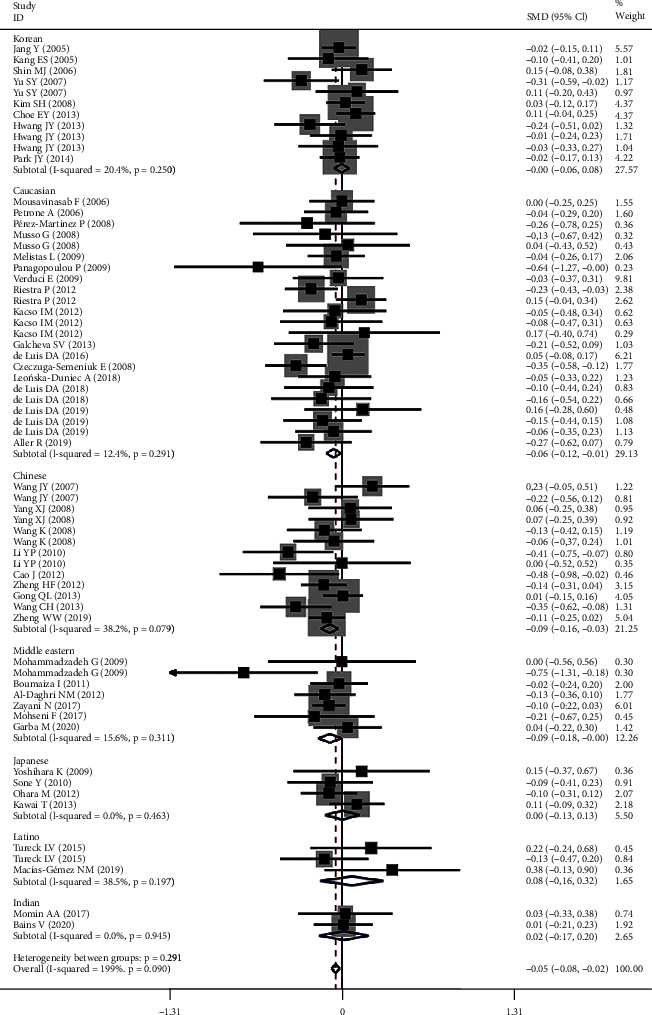


The effects of rs1501299 on lipid profile were beneficial (Figure S5–S7 and Figure 3). Subgroup analysis indicated that the significant effect of rs1501299 on lipid profile was primarily in Chinese, Caucasians, and male subjects (please see Table 2 for more details).

### 3.4. Effect of rs266729 on Lipid Profile

**Figure 4:**
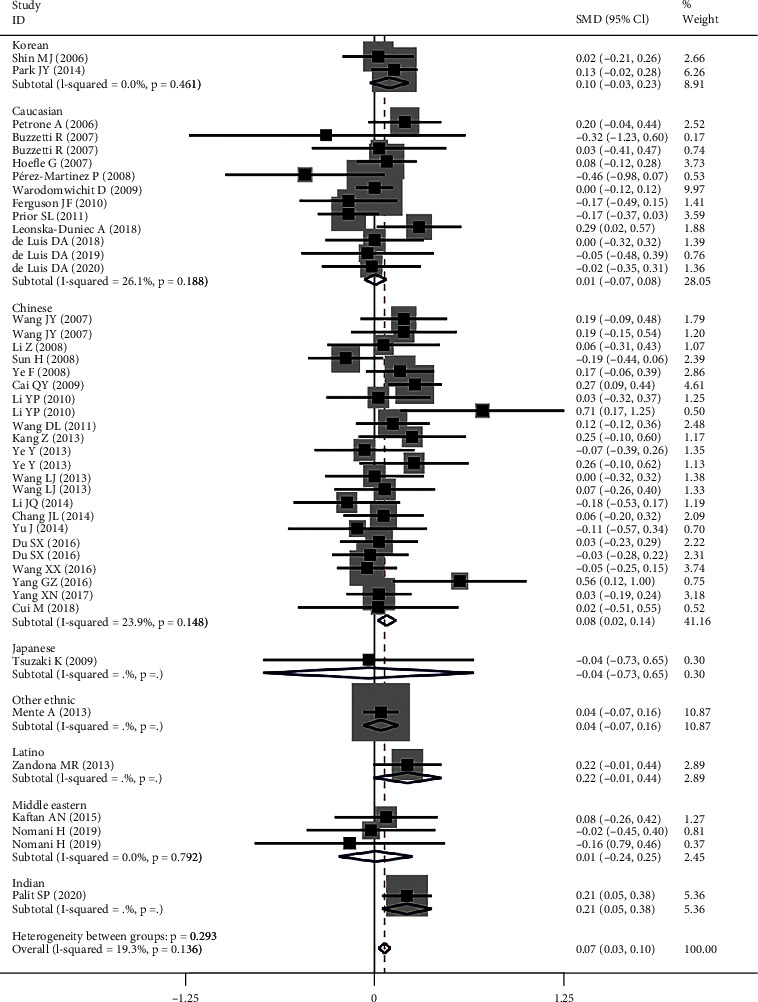


rs2241766 had a harmful effect on lipid profile (Figure S8–S10 and Figure 4). Subgroup analysis indicated that the significant effect of rs1501299 on lipid profile was primarily in Chinese, children, T2DM patients, and CAD patients (please see Table 3 for more details).

### 3.5. Effect of rs2241766, rs1501299, and rs266729 on Adiponectin Levels

rs2241766, rs1501299, and rs266729 had a significant effect on plasma adiponectin levels (Figure S11–S13). Subgroup analysis showed that the effect of rs2241766 and rs150129 on adiponectin levels was primarily in Chinese (please see Table S8 for more details), while the effect of rs266729 on adiponectin levels was primarily in Caucasians (please see Table S8 for more details).

### 3.6. Evaluation of Heterogeneity

Significant heterogeneity was detected in analyzing the effects of adiponectin variants on lipid and adiponectin levels (Tables 1–3 and Table S8). However, the recalculated results did not change significantly after eliminating heterogeneity (see Tables 1–3 and Table S8 for more details), indicating that the analysis results were robust.

### 3.7. Publication Bias Test

No publication bias was detected (see Figure S14–S17 for more details), indicating that the synthetic results were reliable.

## 4. Discussion

Our study indicated that variants of rs2241766, rs1501299, and rs266729 had significant effects on circulating adiponectin and lipid levels. Among them, variants of rs2241766 and rs266729 are atherogenic, while variant rs1501299 is antiatherogenic. *S*ince variants of adiponectin are robustly related to lipid and adiponectin levels in specific populations, it can be helpful for physicians to choose different clinical management to intervention the onset of CVD.

Previous studies showed that variants of rs2241766 [22], rs1501299 [14], and rs266729 [13] may affect adiponectin mRNA splicing, indicating that adiponectin variants may affect adiponectin levels by modulating adiponectin mRNA. The mechanisms underlying adiponectin variants impacted lipid profile have not been elucidated. However, emerging evidence indicated that the effects of adiponectin variants on lipid levels were possibly mediated by the circulating adiponectin levels [23–25].

The present study showed that variants of rs2241766 and rs26672 were associated with higher TG, TC, and LDL-C, as well as lower HDL-C and adiponectin levels (Tables 1 and 3 and Table S8), indicating that variants of rs2241766 and rs26672 decreased adiponectin and caused dyslipidemia. Therefore, rs2241766 and rs266729 should be considered the atherogenic genetic factors. In contrast, variant of rs1501299 was associated with lower TG, TC, and LDL-C, as well as higher HDL-C and adiponectin levels (Table 2 and Table S8), indicating that variant of rs1501299 elevated adiponectin and ameliorated lipid profile. Therefore, rs1501299 should be recognized as an antiatherogenic genetic factor. Intriguingly, the effects of these variants on lipid profile and adiponectin levels can explain, at least in part, the known correlations between the rs2241766, rs266729, and rs1501299 variants and the risk of CAD [18, 19].

The decreased plasma adiponectin (Table S8) was associated with increased TG, TC, and LDL-C, as well as decreased HDL-C levels (Tables 1 and 3), indicating that low levels of adiponectin were linked to an atherogenic lipid profile. In contrast, the increased plasma adiponectin (Table S8) was correlated to decreased TG, TC, and LDL-C, as well as increased HDL-C levels (Table 2), indicating that high levels of adiponectin were linked to an antiatherogenic lipid profile. Taken together, indicating adiponectin was indeed an antiatherogenic molecule, and plasma levels of adiponectin should be recognized as a marker of dyslipidemia.

According to the 2018 ACC/AHA [26], the 2019 ESC/EAS [27], and the adult treatment panel III (ATP III) cholesterol guidelines [28], LDL-C was considered the major cause of CAD and treated as the primary target for therapy, while other lipids were used as the secondary or supplementary therapeutic targets. In the present study, a considerable effect of rs2241766 on LDL-C (SMD = 0.18, 95% CI = 0.04 − 0.32, *P* = 0.01) and TC (SMD = 0.18, 95% CI = 0.05 − 0.31, *P* = 0.01) was observed in males (Table 1). Indicating the males with the rs2241766 variant had an increased risk of CAD. In sharp contrast to rs2241766, substantially decreased LDL-C (SMD = −0.14, 95% CI = −0.26 − −0.02, *P* = 0.03) and TC (SMD = −0.19, 95% CI = −0.31 − −0.07, *P* < 0.01) were observed in males with the rs1501299 variant (Table 2), indicating that males with the rs1501299 variant had reduced susceptibility to CAD. However, whether variant of rs266729 impacted the risk of CAD in males could not be determined due to the absence of data (Table 3). Further clinical trials in males are certainly needed.

Subgroup analysis by ethnicity showed that significantly increased LDL-C, TC, and TG and decreased HDL-C were observed in Chinese with rs2241766 and rs266729 (Tables 1 and 3), indicating that Chinese with variants of rs2241766 and rs266729 were at high risk of dyslipidemia, in other words, Chinese with the rs2241766 and rs266729 variants had an increased risk to develop atherosclerosis or CAD. However, decreased LDL-C and TC were observed in Caucasians with rs1501299 (Table 2), indicating that Caucasians with the rs1501299 variant had a reduced risk of CAD.

Moreover, significant increases in TG and TC, as well as decreases in HDL-C, were detected in T2DM patients with rs2241766 (Table 1), indicating that T2DM patients with the rs2241766 variant had an increased risk of dyslipidemia, but not CAD. Significant increases in HDL-C were detected in T2DM patients with rs1501299 (Table 2), indicating that T2DM patients with the rs1501299 variant were protected against dyslipidemia, whereas significant increases in LDL-C and decreases in HDL-C were detected in T2DM patients with rs266729 (Table 3), indicating that the T2DM patients with the rs266729 variant were at high risk of dyslipidemia and/or CAD.

Notably, a significant increase in LDL-C (SMD = 0.15, 95% CI = 0.05 − 0.25, *P* < 0.01), TC (SMD = 0.15, 95% CI = 0.05 − 0.25, *P* = 0.01), and TG (SMD = 0.16, 95% CI = 0.06 − 0.26, *P* < 0.01) was observed in the children with rs266729 (Table 3), indicating that children with the rs266729 variant were at high risk of dyslipidemia, atherosclerosis, and even early onset of CAD in the future; therefore, these children need our particular attention for early identification.

## 5. Strengths and Limitations

The present meta-analysis has several strengths. For instance, the clinical data of 86,610 individuals were included in the analysis, which increased the reliability of synthetic results due to high statistical power. Secondly, the synthetic results were recalculated after excluding the studies with heterogeneity, which further advanced the preciseness of conclusions drawn in this study and were not likely to be type I errors (false-positive results). However, several limitations of the present study should be noted. Firstly, dyslipidemia is involved in a large number of genes as well as some environmental factors. However, the interactions of the rs2241766, rs1501299, and rs266729 variants with other polymorphic loci or environmental factors on lipid profile have not been investigated in this study due to the lack of the original data from the included studies. In other words, more precise results could have been gained if more detailed individual data were available, or if the stratification analyses based on the environmental factors such as smoking, alcohol consumption, exercise, etc., were performed [29]. Secondly, this meta-analysis only included the studies published in English and Chinese as it was very difficult to get the full papers published in various languages [29]. Thirdly, a protocol (e.g., PROSPERO) had not been preregistered for this meta-analysis due to a huge workload and heavy analytical tasks, which may introduce potential bias to this study.

## 6. Conclusions

The present study indicated that Chinese with the rs2241766 and rs266729 variants were at high risk of dyslipidemia, atherosclerosis, or coronary artery disease (CAD). Males with the rs2241766 variant were at high risk of CAD. Children with the rs266729 variant had a high risk to develop dyslipidemia, atherosclerosis, and even early onset of CAD in the future. These findings are beneficial to clinical physicians to choose different management strategies for cardiovascular disease (CVD) prevention.

## Figures and Tables

**Table 1:** Meta-analysis of adiponectin rs2241766 variant with lipid levels.

Groups or subgroups	Comparisons (subjects)	*P*_H_	SMD (95% CI)	*P*_SMD_
*Overall results*				
TG				
All	120 (29 732)	<0.001	0.07 (0.02-0.12)	<0.01
*Ethnicity*				
Chinese	56 (12 087)	<0.001	0.09 (0.01-0.16)	0.03
Japanese	6 (575)	0.10	0.07 (-0.18-0.31)	0.58
Korean	9 (5 622)	0.09	-0.00 (-0.07-0.07)	0.93
Caucasian	27 (6 099)	0.38	0.02 (-0.05-0.08)	0.65
Latino	6 (695)	0.43	-0.01 (-0.17-0.16)	0.95
Indian	5 (2 762)	<0.001	0.42 (0.04-0.80)	0.03
Middle eastern	10 (1 620)	<0.01	0.06 (-0.13-0.25)	0.53
*Gender*				
Male	5 (1 131)	0.11	0.01 (-0.18-0.20)	0.93
Female	15 (3 121)	<0.001	0.09 (-0.12-0.30)	0.39
*Disease status*				
CAD	4 (817)	0.24	-0.01 (-0.19-0.16)	0.88
T2DM	25 (6 328)	<0.001	0.27 (0.12-0.42)	<0.001
Obesity	13 (1 715)	<0.001	0.06 (-0.20-0.31)	0.66
Mets	3 (357)	0.17	-0.05 (-0.39-0.30)	0.80
PCOS	4 (504)	0.31	-0.09 (-0.34-0.16)	0.48
NAFLD	3 (417)	0.78	0.03 (-0.17-0.23)	0.79
Healthy subjects	41 (10 421)	<0.001	0.03 (-0.03-0.10)	0.34
Children subjects	7 (1 449)	0.01	-0.05 (-0.27-0.17)	0.64
TC				
All	118 (27 932)	<0.001	0.06 (0.01-0.10)	0.02
*Ethnicity*				
Chinese	55 (10 894)	<0.001	0.09 (0.01-0.17)	0.03
Japanese	5 (381)	0.27	0.08 (-0.16-0.32)	0.52
Korean	9 (5 672)	0.05	-0.03 (-0.10-0.05)	0.51
Caucasian	24 (5 103)	0.29	0.01 (-0.07-0.09)	0.79
Latino	6 (695)	<0.01	-0.03 (-0.39-0.32)	0.87
Indian	4 (1 763)	0.19	0.07 (-0.09-0.23)	0.40
Middle eastern	11 (2 741)	0.09	0.03 (-0.09-0.15)	0.59
*Gender*				
Male	5 (1 131)	0.32	0.18 (0.04-0.33)	0.01
Female	14 (2 269)	0.02	0.08 (-0.06-0.23)	0.27
*Disease status*				
CAD	5 (895)	0.47	0.22 (0.09-0.36)	<0.001
T2DM	26 (6 272)	<0.001	0.14 (0.03-0.25)	0.02
Obesity	15 (1 869)	<0.001	0.19 (0.02-0.37)	0.03
PCOS	4 (504)	0.33	-0.12 (-0.36-0.13)	0.35
Healthy subjects	41 (9 778)	<0.001	0.03 (-0.06-0.12)	0.51
Children subjects	8 (1 616)	<0.001	0.12 (-0.14-0.38)	0.36
LDL-C				
All	94 (22 900)	<0.001	0.09 (0.04-0.14)	<0.001
*Ethnicity*				
Chinese	43 (8 954)	<0.001	0.17 (0.08-0.26)	<0.001
Japanese	3 (239)	0.51	0.13 (-0.13-0.39)	0.32
Korean	7 (4 220)	0.53	-0.03 (-0.09-0.03)	0.29
Caucasian	19 (4 258)	0.34	0.07 (-0.01-0.15)	0.09
Latino	6 (695)	0.60	-0.12 (-0.29-0.05)	0.16
Indian	3 (1 442)	0.56	0.03 (-0.10-0.17)	0.63
Middle eastern	10 (2 681)	0.39	0.00 (-0.08-0.09)	0.93
Other ethnic	3 (411)	0.15	-0.02 (-0.35-0.32)	0.93
*Gender*				
Male	5 (1 068)	0.58	0.18 (0.04-0.32)	0.01
Female	10 (1 197)	0.30	-0.01 (-0.16-0.15)	0.91
*Disease status*				
CAD	3 (757)	0.19	0.14 (-0.05-0.33)	0.14
T2DM	23 (6 086)	<0.001	0.12 (0.00-0.24)	0.05
Obesity	13 (1 571)	<0.001	0.19 (-0.05-0.43)	0.12
PCOS	3 (451)	0.88	-0.10 (-0.34-0.14)	0.43
Healthy subjects	32 (6 998)	<0.001	0.09 (0.00-0.17)	0.04
Children subjects	8 (1 616)	<0.001	0.20 (-0.03-0.42)	0.09
HDL-C				
All	119 (30 380)	<0.001	-0.09 (-0.15--0.03)	<0.01
*Ethnicity*				
Chinese	55 (12 479)	<0.001	-0.12 (-0.21--0.03)	0.01
Japanese	5 (497)	0.77	0.00 (-0.18-0.18)	0.99
Korean	10 (5 762)	0.01	-0.03 (-0.12-0.06)	0.53
Caucasian	24 (5 587)	0.30	0.04 (-0.03-0.11)	0.31
Latino	6 (695)	0.44	-0.13 (-0.29-0.04)	0.14
Indian	4 (1 936)	0.01	-0.19 (-0.44-0.06)	0.14
Middle eastern	11 (2 741)	<0.001	-0.29 (-0.73-0.15)	0.19
Other ethnic	4 (683)	0.09	0.02 (-0.27-0.32)	0.88
*Gender*				
Male	5 (1 068)	0.37	-0.03 (-0.17-0.11)	0.68
Female	11 (1 457)	0.54	0.06 (-0.05-0.18)	0.28
*Disease status*				
CAD	4 (817)	0.36	0.03 (-0.12-0.17)	0.73
T2DM	25 (6 397)	<0.001	-0.16 (-0.28--0.04)	0.01
Obesity	16 (1 959)	<0.001	-0.08 (-0.32-0.17)	0.55
PCOS	3 (451)	0.50	0.15 (-0.09-0.39)	0.23
Healthy subjects	42 (10 304)	<0.001	-0.15 (-0.29--0.01)	0.03
Children subjects	8 (1 616)	0.06	0.02 (-0.15-0.18)	0.86
*Recalculated results that eliminated heterogeneity*				
TG				
All	108 (26 484)	0.10	0.03 (0.01-0.06)	0.01
*Ethnicity*				
Chinese	49 (10 726)	0.05	0.04 (0.00-0.08)	0.03
Japanese	6 (575)	0.10	0.07 (-0.10-0.24)	0.41
Korean	8 (4 597)	0.84	0.03 (-0.03-0.08)	0.40
Caucasian	26 (5 907)	0.80	0.00 (-0.06-0.06)	0.98
Latino	6 (695)	0.43	-0.01 (-0.17-0.16)	0.95
Indian	3 (2 146)	0.10	0.08 (-0.02-0.18)	0.11
Middle eastern	9 (1 566)	0.19	0.03 (-0.07-0.14)	0.53
*Gender*				
Male	5 (1 131)	0.11	-0.01 (-0.13-0.12)	0.94
Female	13 (3 020)	0.81	-0.05 (-0.13-0.03)	0.20
*Disease status*				
CAD	4 (817)	0.24	0.00 (-0.14-0.14)	0.99
T2DM	18 (4 777)	0.58	0.06 (0.00-0.12)	0.05
Obesity	10 (1 266)	0.44	-0.02 (-0.14-0.09)	0.71
Mets	3 (357)	0.17	0.01 (-0.21-0.24)	0.91
PCOS	4 (504)	0.31	-0.10 (-0.33-0.12)	0.37
NAFLD	3 (417)	0.78	0.03 (-0.17-0.23)	0.79
Healthy subjects	39 (9 173)	0.20	0.04 (-0.01-0.08)	0.11
Children subjects	6 (1 302)	0.39	0.02 (-0.09-0.13)	0.74
TC				
All	103 (24 758)	0.10	0.03 (0.00-0.05)	0.04
*Ethnicity*				
Chinese	46 (9 093)	0.14	0.03 (-0.01-0.07)	0.17
Japanese	5 (381)	0.27	0.10 (-0.11-0.31)	0.34
Korean	8 (4 914)	0.37	-0.00 (-0.06-0.06)	0.99
Caucasian	22 (4 651)	0.72	0.04 (-0.03-0.11)	0.25
Latino	4 (585)	0.84	-0.09 (-0.26-0.09)	0.34
Indian	4 (1 763)	0.19	0.06 (-0.06-0.18)	0.36
Middle eastern	11 (2 741)	0.09	0.01 (-0.07-0.09)	0.85
*Gender*				
Male	5 (1 131)	0.32	0.18 (0.05-0.31)	0.01
Female	12 (2 152)	0.50	0.05 (-0.05-0.15)	0.32
*Disease status*				
CAD	5 (895)	0.47	0.22 (0.09-0.36)	<0.001
T2DM	21 (4 937)	0.20	0.08 (0.02-0.14)	0.01
Obesity	14 (1 722)	0.02	0.06 (-0.04-0.16)	0.22
PCOS	3 (451)	0.91	-0.03 (-0.27-0.22)	0.83
Healthy subjects	34 (8 203)	0.81	0.03 (-0.02-0.08)	0.22
Children subjects	6 (1 369)	0.23	0.05 (-0.06-0.16)	0.38
LDL-C				
All	88 (21 117)	0.13	0.03 (0.00-0.06)	0.04
*Ethnicity*				
Chinese	38 (7 929)	0.05	0.06 (0.01-0.10)	0.02
Japanese	3 (239)	0.51	0.13 (-0.13-0.39)	0.32
Korean	6 (3 462)	0.74	-0.01 (-0.08-0.06)	0.76
Caucasian	19 (4 258)	0.34	0.07 (-0.01-0.14)	0.08
Latino	6 (695)	0.60	-0.12 (-0.29-0.05)	0.16
Indian	3 (1 442)	0.56	0.03 (-0.10-0.17)	0.63
Middle eastern	10 (2 681)	0.39	0.00 (-0.08-0.08)	0.92
Other ethnic	3 (411)	0.15	-0.03 (-0.22-0.17)	0.79
*Gender*				
Male	5 (1 068)	0.58	0.18 (0.04-0.32)	0.01
Female	10 (1 197)	0.30	-0.02 (-0.15-0.12)	0.82
*Disease status*				
CAD	3 (757)	0.19	0.16 (0.02-0.30)	0.03
T2DM	20 (4 887)	0.35	0.05 (-0.01-0.11)	0.13
Obesity	11 (1 176)	0.01	0.04 (-0.08-0.16)	0.51
PCOS	3 (451)	0.88	-0.10 (-0.34-0.14)	0.43
Healthy subjects	31 (6 809)	0.56	0.04 (-0.01-0.09)	0.16
Children subjects	7 (1 469)	0.11	0.06 (-0.05-0.17)	0.27
HDL-C				
All	107 (27 703)	0.12	-0.03 (-0.06--0.00)	0.04
*Ethnicity*				
Chinese	48 (11 165)	0.18	-0.05 (-0.09--0.01)	0.03
Japanese	5 (497)	0.77	0.00 (-0.18-0.18)	0.99
Korean	9 (5 004)	0.60	0.02 (-0.03-0.08)	0.41
Caucasian	21 (5 280)	0.75	0.01 (-0.06-0.07)	0.80
Latino	6 (695)	0.44	-0.13 (-0.29-0.04)	0.14
Indian	4 (1 936)	0.01	-0.03 (-0.22-0.16)	0.75
Middle eastern	10 (2 443)	0.51	-0.09 (-0.17--0.01)	0.03
Other ethnic	4 (683)	0.09	0.02 (-0.27-0.32)	0.88
*Gender*				
Male	5 (1 068)	0.37	-0.03 (-0.17-0.11)	0.66
Female	10 (1 240)	0.79	0.01 (-0.12-0.14)	0.89
*Disease status*				
CAD	4 (817)	0.36	0.03 (-0.11-0.16)	0.73
T2DM	21 (5 217)	0.05	0.07 (-0.12--0.01)	0.03
Obesity	15 (1 711)	0.16	-0.01 (-0.11-0.09)	0.86
PCOS	3 (451)	0.50	0.15 (-0.09-0.39)	0.23
Healthy subjects	37 (9 342)	0.42	-0.02 (-0.06-0.03)	0.48
Children subjects	7 (1 449)	0.13	-0.01 (-0.12-0.10)	0.86

SMD: standardized mean difference; 95% CI: 95% confidence interval; *P*_H_: *P*_Heterogeneity_; CAD: coronary artery disease; T2DM: type 2 diabetes mellitus; Mets: metabolic syndrome; PCOS: polycystic ovarian syndrome; NAFLD: nonalcoholic fatty liver disease; TG: triglycerides; TC: total cholesterol; LDL-C: low-density lipoprotein cholesterol; HDL-C: high-density lipoprotein cholesterol.

**Table 2:** Meta-analysis of adiponectin rs1501299 variant with lipid levels.

Groups or subgroups	Comparisons (subjects)	*P*_H_	SMD (95% CI)	*P*_SMD_
*Overall results*				
TG				
All	91 (23 853)	<0.001	-0.05 (-0.12-0.02)	0.14
*Ethnicity*				
Chinese	24 (6 525)	0.04	-0.02 (-0.09-0.05)	0.59
Japanese	8 (1 795)	0.18	-0.01 (-0.13-0.10)	0.82
Korean	13 (5 889)	<0.001	0.11 (-0.06-0.28)	0.20
Caucasian	31 (5 889)	<0.001	-0.09 (-0.17-0.00)	0.05
Latino	4 (441)	0.49	-0.05 (-0.24-0.14)	0.60
Indian	5 (2 278)	<0.001	0.27 (0.03-0.52)	0.03
Middle eastern	6 (1 036)	<0.001	-1.19 (-2.02--0.36)	0.01
*Gender*				
Male	5 (1 053)	0.15	-0.01 (-0.18-0.16)	0.91
Female	10 (2 692)	0.17	-0.01 (-0.11-0.09)	0.88
*Disease status*				
T2DM	23 (5 327)	<0.001	0.03 (-0.17-0.23)	0.76
Hypertension	2 (488)	0.82	-0.09 (-0.27-0.09)	0.32
Obesity	16 (2 966)	<0.001	-0.37 (-0.62--0.12)	<0.01
PCOS	2 (351)	0.05	0.20 (-0.37-0.77)	0.49
Healthy subjects	28 (7 279)	0.11	-0.03 (-0.09-0.03)	0.26
Children subjects	8 (2 694)	0.82	0.07 (-0.00-0.15)	0.06
TC				
All	86 (23 252)	<0.001	-0.03 (-0.11-0.06)	0.52
*Ethnicity*				
Chinese	21 (5 108)	<0.001	0.10 (-0.17-0.36)	0.49
Japanese	7 (1 601)	0.56	-0.04 (-0.14-0.06)	0.41
Korean	13 (5 939)	<0.001	-0.08 (-0.18-0.01)	0.08
Caucasian	27 (5 577)	<0.001	-0.12 (-0.23--0.00)	0.04
Latino	4 (441)	0.54	-0.04 (-0.23-0.15)	0.67
Indian	5 (2 278)	<0.001	0.23 (-0.08-0.54)	0.14
Middle eastern	9 (2 308)	<0.001	-0.10 (-0.29-0.10)	0.34
*Gender*				
Male	5 (1 053)	0.48	-0.19 (-0.31--0.07)	<0.01
Female	9 (1 840)	0.14	-0.05 (-0.17-0.07)	0.38
*Disease status*				
T2DM	20 (4 923)	0.16	-0.03 (-0.10-0.04)	0.40
Obesity	16 (2 966)	<0.001	-0.21 (-0.42--0.00)	0.05
PCOS	2 (351)	0.06	-0.11 (-0.64-0.42)	0.68
NAFLD	2 (145)	0.44	-0.14 (-0.49-0.21)	0.44
Healthy subjects	27 (7 166)	<0.001	-0.06 (-0.14-0.02)	0.15
Children subjects	9 (2 862)	0.07	-0.06 (-0.17-0.05)	0.29
LDL-C				
All	70 (18 731)	<0.001	-0.04 (-0.10-0.03)	0.25
*Ethnicity*				
Chinese	13 (3 473)	0.08	-0.10 (-0.19--0.01)	0.03
Japanese	4 (902)	0.46	0.00 (-0.13-0.13)	0.96
Korean	11 (4 487)	0.25	-0.01 (-0.08-0.06)	0.84
Caucasian	26 (5 241)	<0.01	-0.10 (-0.19--0.02)	0.02
Latino	4 (441)	0.20	0.17 (-0.07-0.42)	0.16
Indian	4 (1 957)	<0.001	0.32 (-0.00-0.65)	0.05
Middle eastern	8 (2 230)	0.01	-0.05 (-0.21-0.12)	0.58
*Gender*				
Male	6 (1 269)	0.01	-0.03 (-0.23-0.17)	0.75
Female	9 (1 840)	0.02	0.03 (-0.12-0.18)	0.72
*Disease status*				
T2DM	18 (4 665)	0.07	-0.06 (-0.14-0.01)	0.10
Obesity	15 (2 904)	<0.01	-0.16 (-0.30--0.03)	0.02
PCOS	2 (351)	0.09	-0.16 (-0.63-0.32)	0.52
NAFLD	2 (145)	0.82	-0.18 (-0.53-0.18)	0.33
Healthy subjects	21 (4 860)	0.03	0.01 (-0.07-0.09)	0.81
Children subjects	7 (2 110)	0.06	-0.08 (-0.21-0.06)	0.25
HDL-C				
All	90 (23 986)	<0.001	0.02 (-0.02-0.07)	0.25
*Ethnicity*				
Chinese	21 (6 018)	0.26	0.07 (0.01-0.13)	0.02
Japanese	7 (1 717)	0.03	-0.05 (-0.20-0.10)	0.54
Korean	14 (6 029)	0.05	0.03 (-0.04-0.10)	0.42
Caucasian	31 (6 136)	<0.001	-0.03 (-0.13-0.07)	0.60
Latino	4 (441)	0.51	-0.03 (-0.22-0.15)	0.72
Indian	4 (1 337)	0.03	0.03 (-0.18-0.24)	0.79
Middle eastern	9 (2 308)	0.18	0.12 (-0.00-0.23)	0.05
*Gender*				
Male	6 (1 269)	0.09	-0.15 (-0.30--0.00)	0.05
Female	10 (1 930)	0.37	-0.03 (-0.12-0.07)	0.59
*Disease status*				
T2DM	21 (5 141)	0.02	0.03 (-0.04-0.11)	0.40
Hypertension	2 (488)	0.72	-0.17 (-0.35-0.01)	0.07
Obesity	17 (3 056)	<0.001	0.07 (-0.08-0.22)	0.38
PCOS	2 (351)	0.36	0.00 (-0.21-0.21)	0.99
Healthy subjects	29 (7 623)	<0.01	-0.02 (-0.09-0.05)	0.60
Children subjects	8 (2 188)	0.58	-0.04 (-0.12-0.05)	0.38
*Recalculated results that eliminated heterogeneity*				
TG				
All	78 (19 776)	0.18	-0.04 (-0.07--0.01)	<0.01
*Ethnicity*				
Chinese	23 (6 447)	0.19	-0.03 (-0.08-0.02)	0.20
Japanese	8 (1 795)	0.18	-0.02 (-0.11-0.07)	0.66
Korean	9 (4 116)	0.06	-0.05 (-0.11-0.01)	0.09
Caucasian	28 (5 553)	0.31	-0.04 (-0.10-0.01)	0.10
Latino	4 (441)	0.49	-0.05 (-0.24-0.14)	0.60
Indian	3 (787)	0.85	0.05 (-0.10-0.20)	0.52
Middle eastern	3 (637)	0.58	-0.07 (-0.22-0.09)	0.41
*Gender*				
Male	5 (1 053)	0.15	-0.00 (-0.12-0.12)	0.96
Female	10 (2 692)	0.17	-0.03 (-0.10-0.05)	0.50
*Disease status*				
T2DM	19 (4 498)	0.47	-0.02 (-0.07-0.04)	0.62
Hypertension	2 (488)	0.82	-0.09 (-0.27-0.09)	0.32
Obesity	10 (2 234)	0.22	-0.03 (-0.11-0.06)	0.57
PCOS	2 (351)	0.05	0.05 (-0.16-0.26)	0.65
Healthy subjects	27 (6 254)	0.31	-0.05 (-0.10-0.00)	0.05
Children subjects	8 (2 694)	0.82	0.07 (-0.00-0.15)	0.06
TC				
All	76 (20 042)	0.26	-0.05 (-0.07--0.02)	<0.001
*Ethnicity*				
Chinese	19 (4 315)	0.05	-0.03 (-0.09-0.03)	0.28
Japanese	7 (1 601)	0.56	-0.04 (-0.14-0.06)	0.41
Korean	12 (5 512)	0.45	-0.04 (-0.09-0.01)	0.15
Caucasian	24 (5 204)	0.16	-0.07 (-0.12--0.01)	0.02
Latino	4 (441)	0.54	-0.04 (-0.23-0.15)	0.67
Indian	3 (787)	0.96	0.02 (-0.13-0.17)	0.79
Middle eastern	7 (2 182)	0.69	-0.08 (-0.16-0.01)	0.10
*Gender*				
Male	5 (1 053)	0.48	-0.19 (-0.31--0.07)	<0.01
Female	9 (1 840)	0.14	-0.05 (-0.14-0.04)	0.29
*Disease status*				
T2DM	19 (4 790)	0.32	-0.05 (-0.11-0.01)	0.08
Obesity	12 (2 543)	0.25	-0.02 (-0.09-0.06)	0.71
PCOS	—	—	—	—
NAFLD	2 (145)	0.44	-0.14 (-0.49-0.21)	0.44
Healthy subjects	25 (6 663)	0.33	-0.04 (-0.08-0.01)	0.15
Children subjects	9 (2 862)	0.07	-0.04 (-0.12-0.03)	0.27
LDL-C				
All	63 (16 580)	0.09	-0.05 (-0.08--0.02)	<0.01
*Ethnicity*				
Chinese	13 (3 473)	0.08	-0.09 (-0.16--0.03)	0.01
Japanese	4 (902)	0.46	0.00 (-0.13-0.13)	0.96
Korean	11 (4 487)	0.25	-0.00 (-0.06-0.06)	0.93
Caucasian	23 (4 824)	0.29	-0.06 (-0.12--0.01)	0.03
Latino	3 (274)	0.20	0.08 (-0.16-0.32)	0.54
Indian	2 (466)	0.95	0.02 (-0.17-0.20)	0.88
Middle eastern	7 (2 154)	0.31	-0.09 (-0.18-0.00)	0.04
*Gender*				
Male	5 (1 053)	0.21	-0.14 (-0.26--0.02)	0.03
Female	8 (1 673)	0.04	-0.02 (-0.12-0.08)	0.69
*Disease status*				
T2DM	18 (4 665)	0.07	-0.05 (-0.10-0.01)	0.12
Obesity	13 (2 703)	0.14	-0.03 (-0.11-0.05)	0.46
PCOS	—	—	—	—
NAFLD	2 (145)	0.82	-0.18 (-0.53-0.18)	0.33
Healthy subjects	18 (4 401)	0.71	-0.04 (-0.10-0.02)	0.20
Children subjects	7 (2 110)	0.06	-0.05 (-0.14-0.03)	0.21
HDL-C				
All	80 (22 255)	0.16	0.04 (0.01-0.06)	0.01
*Ethnicity*				
Chinese	21 (6 018)	0.26	0.07 (0.02-0.12)	0.01
Japanese	6 (1 371)	0.28	0.01 (-0.10-0.12)	0.87
Korean	14 (6 029)	0.05	0.02 (-0.04-0.07)	0.53
Caucasian	24 (5 230)	0.39	0.02 (-0.03-0.08)	0.47
Latino	4 (441)	0.51	-0.03 (-0.22-0.15)	0.72
Indian	3 (1 187)	0.62	0.14 (0.02-0.26)	0.02
Middle eastern	8 (1 979)	0.58	0.04 (-0.06-0.13)	0.45
*Gender*				
Male	5 (1 053)	0.65	-0.09 (-0.21-0.03)	0.16
Female	10 (1 930)	0.37	-0.03 (-0.12-0.06)	0.57
*Disease status*				
T2DM	19 (4 645)	0.48	0.06 (0.01-0.12)	0.03
Hypertension	2 (488)	0.72	-0.17 (-0.35-0.01)	0.07
Obesity	14 (2 595)	0.43	0.10 (0.02-0.18)	0.01
PCOS	2 (351)	0.36	0.00 (-0.21-0.21)	0.99
Healthy subjects	27 (7 337)	0.14	-0.00 (-0.05-0.05)	0.98
Children subjects	8 (2 188)	0.58	-0.04 (-0.12-0.05)	0.38

SMD: standardized mean difference; 95% CI: 95% confidence interval; *P*_H_: *P*_Heterogeneity_; T2DM: type 2 diabetes mellitus; PCOS: polycystic ovarian syndrome; NAFLD: nonalcoholic fatty liver disease; TG: triglycerides; TC: total cholesterol; LDL-C: low-density lipoprotein cholesterol; HDL-C: high-density lipoprotein cholesterol.

**Table 3:** Meta-analysis of adiponectin rs266729 variant with lipid levels.

Groups or subgroups	Comparisons (subjects)	*P*_H_	SMD (95% CI)	*P*_SMD_
*Overall results*				
TG				
All	62 (17 815)	<0.001	0.08 (0.01-0.16)	0.03
*Ethnicity*				
Chinese	37 (8 612)	<0.001	0.11 (-0.01-0.23)	0.08
Japanese	2 (1 919)	0.25	0.03 (-0.26-0.31)	0.87
Korean	3 (1 822)	0.86	0.09 (-0.01-0.18)	0.07
Caucasian	13 (2 926)	<0.01	0.02 (-0.10-0.15)	0.72
Middle eastern	3 (290)	0.64	-0.15 (-0.40-0.09)	0.22
*Gender*				
Female	6 (1 816)	<0.001	0.38 (-0.07-0.84)	0.10
*Disease status*				
CAD	3 (448)	<0.01	0.48 (-0.05-1.01)	0.08
T2DM	13 (2 454)	<0.001	-0.06 (-0.23-0.10)	0.45
Obesity	10 (1 473)	0.24	0.10 (-0.03-0.22)	0.13
Mets	4 (3 334)	0.18	-0.09 (-0.20-0.02)	0.11
Healthy subjects	15 (4 374)	<0.01	0.06 (-0.05-0.17)	0.32
Children subjects	4 (1 497)	0.18	0.18 (0.04-0.31)	0.01
TC				
All	60 (15 635)	<0.001	0.09 (0.02-0.16)	0.02
*Ethnicity*				
Chinese	36 (7 763)	<0.001	0.14 (0.02-0.26)	0.02
Japanese	2 (1 919)	0.84	0.06 (-0.03-0.15)	0.17
Korean	2 (970)	0.54	0.12 (-0.01-0.25)	0.07
Caucasian	14 (3 653)	0.10	-0.00 (-0.09-0.09)	0.98
Middle eastern	4 (445)	0.06	-0.07 (-0.40-0.25)	0.65
*Gender*				
Female	5 (964)	<0.001	0.51 (-0.25-1.27)	0.19
*Disease status*				
CAD	3 (448)	0.50	0.26 (0.08-0.45)	0.01
T2DM	13 (2 454)	<0.001	0.06 (-0.11-0.24)	0.48
Obesity	11 (2 545)	0.29	0.04 (-0.05-0.14)	0.36
Mets	3 (2 485)	0.26	0.01 (-0.14-0.15)	0.94
Healthy subjects	13 (2 365)	0.05	0.13 (0.00-0.25)	0.04
Children subjects	4 (1 497)	0.21	0.15 (0.02-0.28)	0.03
LDL-C				
All	53 (13 793)	<0.001	0.14 (0.05-0.23)	<0.01
*Ethnicity*				
Chinese	29 (6 088)	<0.001	0.24 (0.08-0.41)	<0.01
Korean	2 (970)	0.46	0.10 (-0.03-0.23)	0.13
Caucasian	14 (4 216)	<0.01	0.02 (-0.09-0.13)	0.70
Middle eastern	4 (445)	0.30	-0.12 (-0.35-0.10)	0.27
*Gender*				
Female	5 (964)	<0.001	0.48 (-0.38-1.34)	0.27
*Disease status*				
CAD	3 (448)	<0.01	0.37 (-0.17-0.92)	0.18
T2DM	10 (2 024)	<0.001	0.23 (0.01-0.45)	0.05
Obesity	11 (2 545)	0.14	0.07 (-0.04-0.18)	0.21
Mets	2 (598)	0.46	-0.09 (-0.31-0.14)	0.46
Healthy subjects	13 (3 421)	0.16	0.12 (0.03-0.22)	0.01
Children subjects	4 (1 497)	0.80	0.15 (0.05-0.25)	<0.01
HDL-C				
All	57 (15 792)	<0.001	-0.08 (-0.14--0.03)	<0.01
*Ethnicity*				
Chinese	32 (7 169)	<0.001	-0.08 (-0.16--0.01)	0.03
Japanese	2 (1 919)	0.93	-0.03 (-0.12-0.06)	0.51
Korean	2 (970)	0.48	0.04 (-0.09-0.16)	0.58
Caucasian	15 (3 818)	0.24	-0.07 (-0.15-0.02)	0.11
Middle eastern	4 (445)	<0.001	-0.29 (-0.87-0.30)	0.34
*Gender*				
Female	5 (964)	0.04	0.08 (-0.15-0.31)	0.47
*Disease status*				
CAD	3 (448)	0.11	-0.06 (-0.36-0.24)	0.70
T2DM	12 (2 256)	<0.001	-0.13 (-0.33-0.08)	0.22
Obesity	11 (2 545)	0.35	-0.03 (-0.12-0.06)	0.57
Mets	4 (3 334)	0.19	-0.10 (-0.21-0.01)	0.08
Healthy subjects	13 (3 421)	0.31	-0.06 (-0.14-0.03)	0.18
Children subjects	4 (1 497)	0.78	0.06 (-0.04-0.16)	0.25
*Recalculated results that eliminated heterogeneity*				
TG				
All	51 (13 937)	0.06	0.04 (0.00-0.07)	0.04
*Ethnicity*				
Chinese	28 (5 753)	0.51	0.06 (0.00-0.11)	0.04
Japanese	2 (1 919)	0.25	-0.03 (-0.12-0.06)	0.54
Korean	3 (1 822)	0.86	0.09 (-0.01-0.18)	0.07
Caucasian	12 (2 478)	0.01	0.03 (-0.05-0.10)	0.52
Middle eastern	3 (290)	0.64	-0.15 (-0.40-0.09)	0.22
*Gender*				
Female	5 (1 560)	0.42	0.11 (0.01-0.21)	0.04
*Disease status*				
—	—	—	—	—
T2DM	12 (2 363)	0.48	0.04 (-0.04-0.12)	0.35
Obesity	10 (1 473)	0.24	0.10 (0.00-0.21)	0.05
Mets	2 (2 037)	0.52	-0.03 (-0.12-0.06)	0.54
Healthy subjects	14 (4 070)	0.06	0.07 (0.01-0.13)	0.03
Children subjects	4 (1 497)	0.18	0.16 (0.06-0.26)	<0.01
TC				
All	56 (14 415)	0.07	0.05 (0.01-0.08)	0.01
*Ethnicity*				
Chinese	32 (6 543)	0.27	0.06 (0.01-0.11)	0.02
Japanese	2 (1 919)	0.84	0.06 (-0.03-0.15)	0.17
Korean	2 (970)	0.54	0.12 (-0.01-0.25)	0.07
Caucasian	14 (3 653)	0.10	-0.00 (-0.07-0.07)	0.96
Middle eastern	4 (445)	0.06	-0.10 (-0.30-0.09)	0.30
*Gender*				
Female	4 (708)	0.97	0.12 (-0.03-0.27)	0.12
*Disease status*				
CAD	3 (448)	0.50	0.26 (0.08-0.45)	0.01
T2DM	11 (2 108)	0.29	0.03 (-0.06-0.12)	0.47
Obesity	11 (2 545)	0.29	0.03 (-0.05-0.11)	0.40
Mets	3 (2 485)	0.26	0.04 (-0.05-0.12)	0.38
Healthy subjects	13 (2 365)	0.81	0.14 (0.06-0.23)	<0.01
Children subjects	4 (1 497)	0.21	0.15 (0.05-0.25)	0.01
LDL-C				
All	44 (11 297)	0.14	0.07 (0.03-0.10)	<0.001
*Ethnicity*				
Chinese	23 (4 648)	0.15	0.08 (0.02-0.14)	0.01
Korean	2 (970)	0.46	0.10 (-0.03-0.23)	0.13
Caucasian	12 (3 315)	0.19	0.01 (-0.07-0.08)	0.87
Middle eastern	3 (290)	0.79	0.01 (-0.24-0.25)	0.95
*Gender*				
Female	4 (708)	0.26	0.06 (-0.09-0.21)	0.45
*Disease status*				
—	—	—	—	—
T2DM	9 (1 933)	0.08	0.11 (0.02-0.20)	0.02
Obesity	10 (2 411)	0.95	0.02 (-0.06-0.10)	0.57
Mets	2 (598)	0.46	-0.09 (-0.31-0.14)	0.46
Healthy subjects	13 (3 421)	0.16	0.11 (0.04-0.18)	<0.01
Children subjects	4 (1 497)	0.80	0.15 (0.05-0.25)	<0.01
HDL-C				
All	53 (15 033)	0.12	-0.08 (-0.11--0.05)	<0.001
*Ethnicity*				
Chinese	29 (6 545)	0.12	-0.13 (-0.18--0.08)	<0.001
Japanese	2 (1 919)	0.93	-0.03 (-0.12-0.06)	0.51
Korean	2 (970)	0.48	0.04 (-0.09-0.16)	0.58
Caucasian	15 (3 818)	0.24	-0.06 (-0.13-0.01)	0.08
Middle eastern	3 (310)	0.56	-0.05 (-0.29-0.18)	0.66
*Gender*				
Female	4 (708)	0.69	-0.04 (-0.19-0.11)	0.59
*Disease status*				
CAD	3 (448)	0.11	-0.01 (-0.20-0.18)	0.92
T2DM	9 (1 753)	0.12	-0.15 (-0.25--0.05)	<0.01
Obesity	11 (2 545)	0.35	-0.03 (-0.11-0.05)	0.51
Mets	4 (3 334)	0.19	-0.08 (-0.15--0.00)	0.04
Healthy subjects	13 (3 421)	0.31	-0.05 (-0.12-0.02)	0.16
Children subjects	4 (1 497)	0.78	0.06 (-0.04-0.16)	0.25

SMD: standardized mean difference; 95% CI: 95% confidence interval; *P*_H_: *P*_Heterogeneity_; CAD: coronary artery disease; T2DM: type 2 diabetes mellitus; Mets: metabolic syndrome; TG: triglycerides; TC: total cholesterol; LDL-C: low-density lipoprotein cholesterol; HDL-C: high-density lipoprotein cholesterol.

## Data Availability

All data used to support the findings of this study are included within the article and its Supplementary Materials.

## Supplementary Materials

Supplementary Materials The characteristics of eligible studies were summarized in Table S1. The circulating lipids levels by the genotypes rs2241766, rs1501299, and rs266729 were presented in Table S2, Table S3, and Table S4. The circulating adiponectin levels by the genotypes rs2241766, rs1501299, and rs266729 were presented in Table S5, Table S6, and Table S7. Meta-analysis of adiponectin variants with adiponectin level was presented in Table S8. The full electronic search strategy in PubMed was presented in Figure S1. Forest plot of the meta-analysis between the rs2241766 polymorphism and circulating TG, TC, and HDL-C levels was presented in Figure S2, Figure S3, and Figure S4. Forest plot of the meta-analysis between the rs1501299 polymorphism and circulating TG, TC, and HDL-C levels was presented in Figure S5, Figure S6, and Figure S7. Forest plot of the meta-analysis between the rs266729 polymorphism and circulating TG, TC and HDL-C levels was presented in Figure S8, Figure S9, and Figure S10. Forest plot of the meta-analysis between adiponectin polymorphisms and circulating adiponectin levels was presented in Figure S11, Figure S12, and Figure S13. Begg's funnel plot evaluates the publication bias of the effects of adiponectin polymorphisms on lipid levels were presented in Figure S14, Figure S155, and Figure S16. Begg's funnel plot evaluating the publication bias of the effects of adiponectin polymorphisms on circulating adiponectin levels was presented in Figure S17.
