# Supplementary material for: Effect of Adiponectin Variant on Lipid Profile and Plasma Adiponectin Levels: A Multicenter Systematic Review and Meta-Analysis
Source: Cardiovasc Ther. 2022 Jul 7;2022:4395266. doi: 10.1155/2022/4395266 (PMC9283072; doi:10.1155/2022/4395266)
Supplement: Supplementary Materials — The characteristics of eligible studies were summarized in Table S1. The circulating lipids levels by the genotypes rs2241766, rs1501299, and rs266729 were presented in Table S2, Table S3, and Table S4. The circulating adiponectin levels by the genotypes rs2241766, rs1501299, and rs266729 were presented in Table S5, Table S6, and Table S7. Meta-analysis of adiponectin variants with adiponectin level was presented in Table S8. The full electronic search strategy in PubMed was presented in Figure S1. Forest plot of the meta-analysis between the rs2241766 polymorphism and circulating TG, TC, and HDL-C levels was presented in Figure S2, Figure S3, and Figure S4. Forest plot of the meta-analysis between the rs1501299 polymorphism and circulating TG, TC, and HDL-C levels was presented in Figure S5, Figure S6, and Figure S7. Forest plot of the meta-analysis between the rs266729 polymorphism and circulating TG, TC and HDL-C levels was presented in Figure S8, Figure S9, and Figure S10. Forest plot of the meta-analysis between adiponectin polymorphisms and circulating adiponectin levels was presented in Figure S11, Figure S12, and Figure S13. Begg's funnel plot evaluates the publication bias of the effects of adiponectin polymorphisms on lipid levels were presented in Figure S14, Figure S155, and Figure S16. Begg's funnel plot evaluating the publication bias of the effects of adiponectin polymorphisms on circulating adiponectin levels was presented in Figure S17. [file 4395266.f1.docx]

**Supplemental Tables:**

**Table S1.** Characteristics of the included studies.

**Table S2.** Circulating lipids levels by the genotypes of rs2241766.

**Table S3.** Circulating lipids levels by the genotypes of rs1501299.

**Table S4.** Circulating lipids levels by the genotypes of rs266729.

**Table S5.** Circulating adiponectin levels by the genotypes of rs2241766.

**Table S6.** Circulating adiponectin levels by the genotypes of rs1501299.

**Table S7.** Circulating adiponectin levels by the genotypes of rs266729.

**Table S8.** Meta-analysis of Adiponectin variants with adiponectin levels.

**Supplemental Figures:**

**Figure S1.** The full electronic search strategy in Pubmed.

**Figure S2.** Forest plot of the meta-analysis between the rs2241766 polymorphism and circulating TG levels.

**Figure S3.** Forest plot of the meta-analysis between the rs2241766 polymorphism and circulating TC levels.

**Figure S4.** Forest plot of the meta-analysis between the rs2241766 polymorphism and circulating HDL-C levels.

**Figure S5.** Forest plot of the meta-analysis between the rs1501299 polymorphism and circulating TG levels.

**Figure S6.** Forest plot of the meta-analysis between the rs1501299 polymorphism and circulating TC levels.

**Figure S7.** Forest plot of the meta-analysis between the rs1501299 polymorphism and circulating HDL-C levels.

**Figure S8.** Forest plot of the meta-analysis between the rs266729 polymorphism and circulating TG levels.

**Figure S9.** Forest plot of the meta-analysis between the rs266729 polymorphism and circulating TC levels.

**Figure S10.** Forest plot of the meta-analysis between the rs266729 polymorphism and circulating HDL-C levels.

**Figure S11.** Forest plot of the meta-analysis between the rs2241766 polymorphism and circulating adiponectin levels.

**Figure S12.** Forest plot of the meta-analysis between the rs266729 polymorphism and circulating adiponectin levels.

**Figure S13.** Forest plot of the meta-analysis between the rs1501299 polymorphism and circulating adiponectin levels.

**Figure S14.** Begg’s funnel plot evaluates the publication bias of the effects of the rs2241766 polymorphism on lipid levels.

**Figure S15.** Begg’s funnel plot evaluates the publication bias of the effects of the rs1501299 polymorphism on lipid levels.

**Figure S16.** Begg’s funnel plot evaluates the publication bias of the effects of the rs266729 polymorphism on lipid levels.

**Figure S17.** Begg’s funnel plot evaluates the publication bias of the effects of rs2241766, rs1501299 and rs266729 on circulating adiponectin levels.

**Table S1.** Characteristics of the included studies.

| **First author, reference** | **year** | **Ethnicity** | **Gender** | **Study population** | **Outcomes** |
| --- | --- | --- | --- | --- | --- |
| Schäffler A [R1] | 2000 | Caucasian | M/F | Obesity patients and control subjects | TG, TC, LDL-C, HDL-C |
| Zietz B [R2] | 2001 | Caucasian | M/F | T2DM patients | TG, TC, LDL-C, HDL-C |
| Yang WS [R3] | 2003 | Chinese | M/F | Obesity patients and control subjects | TG, TC |
| Filippi E [R4] | 2004 | Caucasian | M/F | Healthy subjects | TG, TC, LDL-C, HDL-C |
| Stenvinkel P [R5] | 2004 | Caucasian | M/F | End-stage renal disease patients | TG, TC, HDL-C |
| Du PF [R6] | 2004 | Chinese | M/F | T2DM patients and control subjects | HDL-C, Adiponectin |
| Yoshioka K [R7] | 2004 | Chinese | M/F | T2DM patients | TG, TC, HDL-C, Adiponectin |
| Schäffler A [R8] | 2004 | Caucasian | M/F | T2DM patients and control subjects | Adiponectin |
| Fumeron F [R9] | 2004 | Caucasian | M | Healthy subjects | Adiponectin |
| Fumeron F [R9] | 2004 | Caucasian | F | Healthy subjects | Adiponectin |
| González-Sánchez JL [R10] | 2005 | Caucasian | M/F | Healthy subjects | TG, TC, HDL-C, Adiponectin |
| Xita N [R11] | 2005 | Caucasian | F | PCOS patients | TG, TC, LDL-C, HDL-C, Adiponectin |
| Berthier MT [R12] | 2005 | Caucasian | M | Healthy subjects | LDL-C, HDL-C, Adiponectin |
| Nakatani K [R13] | 2005 | Japanese | M/F | Healthy subjects | TG, HDL-C |
| Jang Y [R14] | 2005 | Korean | M/F | Healthy subjects | TC, LDL-C, HDL-C, Adiponectin |
| Lee YY [R15] | 2005 | Korean | M/F | Healthy subjects | TG, TC, HDL-C, Adiponectin |
| Kang ES [R16] | 2005 | Korean | M/F | T2DM patients | TG, TC, LDL-C, HDL-C, Adiponectin |
| Ukkola O [R17] | 2005 | Caucasian | M/F | Healthy subjects | TG, TC, HDL-C |
| Ukkola O [R17] | 2005 | Other ethnic | M/F | Healthy subjects | TG, TC, HDL-C |
| Haap M [R18] | 2005 | Caucasian | F | PCOS patients | TG, TC |
| Haap M [R18] | 2005 | Caucasian | F | Healthy subjects | TG, TC |
| Vasseur F [R19] | 2005 | Caucasian | M/F | Obesity patients | Adiponectin |
| Pollin TI [R20] | 2005 | Caucasian | M/F | Healthy subjects | Adiponectin |
| Tankó LB [R21] | 2005 | Caucasian | F | Healthy subjects | Adiponectin |
| Shin MJ [R22] | 2006 | Korean | M/F | Obesity patients | TG, TC, LDL-C, HDL-C, Adiponectin |
| Mousavinasab F [R23] | 2006 | Caucasian | M | Healthy subjects | TG, TC, LDL-C, HDL-C, Adiponectin |
| Yan WL [R24] | 2006 | Chinese | M/F | Healthy subjects | TG, TC, LDL-C, HDL-C |
| Petrone A [R25] | 2006 | Caucasian | M/F | Obesity patients | TG, TC, LDL-C, HDL-C, Adiponectin |
| Tso AW [R26] | 2006 | Chinese | M/F | IGT patients | HDL-C |
| Iacobellis G [R27] | 2006 | Caucasian | M/F | Obesity patients | TG, TC, HDL-C, Adiponectin |
| He L [R28] | 2006 | Chinese | M/F | T2DM patients | TG, TC, Adiponectin |
| He L [R28] | 2006 | Chinese | M/F | Healthy subjects | TG, TC, Adiponectin |
| Woo JG [R29] | 2006 | Caucasian | M/F | Healthy subjects | Adiponectin |
| Woo JG [R29] | 2006 | African | M/F | Healthy subjects | Adiponectin |
| Schwarz PE [R30] | 2006 | Caucasian | M/F | T2DM patients | Adiponectin |
| Wang JY [R31] | 2007 | Chinese | M/F | T2DM patients | TG, TC, LDL-C, HDL-C |
| Wang JY [R31] | 2007 | Chinese | M/F | Healthy subjects | TG, TC, LDL-C, HDL-C |
| Yu SY [R32] | 2007 | Korean | M | T2DM patients | TG, TC, LDL-C, HDL-C, Adiponectin |
| Yu SY [R32] | 2007 | Korean | F | T2DM patients | TG, TC, LDL-C, HDL-C, Adiponectin |
| Buzzetti R [R33] | 2007 | Caucasian | M | Obesity patients | TG, TC, LDL-C, HDL-C, Adiponectin |
| Buzzetti R [R33] | 2007 | Caucasian | F | Obesity patients | TG, TC, LDL-C, HDL-C, Adiponectin |
| Hoefle G [R34] | 2007 | Caucasian | M/F | CAD patients and control subjects | TG, TC, LDL-C, HDL-C |
| Katsuda Y [R35] | 2007 | Japanese | M/F | Obesity patients | TG, TC, HDL-C, Adiponectin |
| Jeng JR [R36] | 2007 | Chinese | M | Hypertension patients and control subjects | TG, TC, Adiponectin |
| Jeng JR [R36] | 2007 | Chinese | F | Hypertension patients and control subjects | TG, TC, Adiponectin |
| Li LL [R37] | 2007 | Chinese | M/F | T2DM patients | Adiponectin |
| Li LL [R37] | 2007 | Chinese | M/F | Healthy subjects | Adiponectin |
| Ishibashi K [R38] | 2007 | Japanese | M/F | Healthy subjects | Adiponectin |
| Zhang H [R39] | 2007 | Chinese | M/F | T2DM patients | TG, TC, LDL-C, HDL-C |
| Li Z [R40] | 2008 | Chinese | M/F | T2DM patients | TG, TC, LDL-C, HDL-C |
| Sun H [R41] | 2008 | Chinese | M/F | T2DM patients | TG, TC, LDL-C, HDL-C |
| Wang ZL [R42] | 2008 | Chinese | M/F | Mets patients and control subjects | TG, TC, LDL-C, HDL-C, Adiponectin |
| Wang ZL [R42] | 2008 | Chinese | M/F | Healthy subjects | TG, TC, LDL-C, HDL-C, Adiponectin |
| Chen QY [R43] | 2008 | Chinese | M/F | T2DM patients and control subjects | TG, TC, LDL-C, HDL-C |
| Yang XJ [R44] | 2008 | Chinese | M/F | Cerebral infarction patients | TG, TC, LDL-C, HDL-C |
| Yang XJ [R44] | 2008 | Chinese | M/F | Healthy subjects | TG, TC, LDL-C, HDL-C |
| Ai ZH [R45] | 2008 | Chinese | M/F | T2DM patients | TG, TC, LDL-C, HDL-C, Adiponectin |
| Pérez-Martínez P [R46] | 2008 | Caucasian | M/F | Healthy subjects | TG, TC, LDL-C, HDL-C |
| Jang Y [R47] | 2008 | Korean | F | Healthy subjects | TG, Adiponectin |
| Potapov VA [R48] | 2008 | Caucasian | M/F | T2DM patients | TG, TC, LDL-C, HDL-C |
| Potapov VA [R48] | 2008 | Caucasian | M/F | Healthy subjects | TG, TC, LDL-C, HDL-C |
| Musso G [R49] | 2008 | Caucasian | M/F | NAFLD patients and control subjects | TG, TC, LDL-C, HDL-C, Adiponectin |
| Musso G [R49] | 2008 | Caucasian | M/F | Healthy subjects | TG, TC, LDL-C, HDL-C, Adiponectin |
| Kim SH [R50] | 2008 | Korean | M/F | T2DM patients | TG, TC, LDL-C, HDL-C, Adiponectin |
| Guo ZX [R51] | 2008 | Chinese | M/F | T2DM patients | TG, TC |
| Guo ZX [R51] | 2008 | Chinese | M/F | T2DM patients | TG, TC |
| Guo ZX [R51] | 2008 | Chinese | M/F | Healthy subjects | TG, TC |
| Ye F [R52] | 2008 | Chinese | M/F | T2DM patients and control subjects | TG, TC, LDL-C, HDL-C |
| Zietz B [R53] | 2008 | Caucasian | M/F | T2DM patients | Adiponectin |
| Kyriakou T [R54] | 2008 | Caucasian | F | Healthy subjects | Adiponectin |
| Kyriakou T [R54] | 2008 | Caucasian | F | Healthy subjects | Adiponectin |
| Yang M [R55] | 2008 | Chinese | M/F | T2DM patients | Adiponectin |
| Yang M [R55] | 2008 | Chinese | M/F | IGT patients | Adiponectin |
| Yang M [R55] | 2008 | Chinese | M/F | Healthy subjects | Adiponectin |
| Mohammadzadeh G [R56] | 2009 | Middle eastern | M/F | Obesity patients | TG, TC, LDL-C, HDL-C, Adiponectin |
| Mohammadzadeh G [R56] | 2009 | Middle eastern | M/F | Obesity patients | TG, TC, LDL-C, HDL-C, Adiponectin |
| Shu F [R57] | 2009 | Chinese | M/F | Schizophrenic patients | TG, TC, LDL-C, HDL-C |
| Shu F [R57] | 2009 | Chinese | M/F | Healthy subjects | TG, TC, LDL-C, HDL-C |
| Yoshihara K [R58] | 2009 | Japanese | F | PCOS patients | TG, TC, LDL-C, HDL-C |
| Tsuzaki K [R59] | 2009 | Japanese | F | Healthy subjects | TG, TC, LDL-C, HDL-C, Adiponectin |
| Melistas L [R60] | 2009 | Caucasian | F | Healthy subjects | TG, TC, LDL-C, HDL-C, Adiponectin |
| Panagopoulou P [R61] | 2009 | Caucasian | M/F | Obesity patients | TG, TC, LDL-C, HDL-C, Adiponectin |
| Verduci E [R62] | 2009 | Caucasian | M/F | Obesity patients | TG, TC, LDL-C, HDL-C |
| Wang K [R63] | 2009 | Chinese | M/F | T2DM patients | TG, LDL-C, HDL-C |
| Wang K [R63] | 2009 | Chinese | M/F | Healthy subjects | TG, LDL-C, HDL-C |
| Cao LF [R64] | 2009 | Chinese | M/F | Obesity patients and control subjects | TG, TC |
| Warodomwichit D [R65] | 2009 | Caucasian | M/F | Obesity patients | TC, LDL-C, HDL-C |
| Oguri M [R66] | 2009 | Japanese | M/F | Mets patients | TG, TC, HDL-C |
| Prior SL [R67] | 2009 | Caucasian | M/F | CAD patients and control subjects | TG, LDL-C |
| Cai QY [R68] | 2009 | Chinese | M/F | T2DM patients | TG, TC, LDL-C, HDL-C, Adiponectin |
| Sheng TX [R69] | 2009 | Chinese | M/F | T2DM patients and control subjects | TG, TC |
| Sheng TX [R69] | 2009 | Chinese | M/F | T2DM patients and control subjects | TG, TC |
| Chung HK [R70] | 2009 | Korean | M/F | T2DM patients | Adiponectin |
| Sone Y [R71] | 2010 | Japanese | M | Healthy subjects | TG, TC, LDL-C, HDL-C |
| Ronconi V [R72] | 2010 | Caucasian | M/F | Primary aldosteronism patients | TG, HDL-C |
| Ronconi V [R72] | 2010 | Caucasian | M/F | Hypertension patients | TG, HDL-C |
| Xu L [R73] | 2010 | Chinese | M/F | CAD patients | TG, TC, LDL-C, HDL-C, Adiponectin |
| Xu L [R73] | 2010 | Chinese | M/F | Healthy subjects | TG, TC, LDL-C, HDL-C, Adiponectin |
| Li YP [R74] | 2010 | Chinese | M/F | T2DM patients | TG, TC, LDL-C, HDL-C |
| Li YP [R74] | 2010 | Chinese | M/F | Healthy subjects | TG, TC, LDL-C, HDL-C |
| Huang MC [R75] | 2010 | Chinese | M/F | Dyslipidemia patients and control subjects | TG, TC, Adiponectin |
| Ferguson JF [R76] | 2010 | Caucasian | M/F | Mets patients | TG, TC, LDL-C, HDL-C, Adiponectin |
| Youpeng B [R77] | 2010 | Chinese | F | Severe preeclamptic patients | Adiponectin |
| Youpeng B [R77] | 2010 | Chinese | F | Mild preeclamptic patients | Adiponectin |
| Youpeng B [R77] | 2010 | Chinese | F | Healthy subjects | Adiponectin |
| Demirci H [R78] | 2010 | Middle eastern | F | PCOS patients | Adiponectin |
| Demirci H [R78] | 2010 | Middle eastern | F | Healthy subjects | Adiponectin |
| Passariello CL [R79] | 2010 | Caucasian | F | Healthy subjects | Adiponectin |
| Liang YL [R80] | 2011 | Chinese | M/F | CAD patients | TC |
| Yang HY [R81] | 2011 | Chinese | M/F | T2DM patients | TG, TC, LDL-C, HDL-C |
| Zhou Q [R82] | 2011 | Chinese | M/F | T2DM patients | TC, Adiponectin |
| Zhou Q [R82] | 2011 | Chinese | M/F | Healthy subjects | TC, Adiponectin |
| Leu HB [R83] | 2011 | Chinese | M/F | Mets patients and control subjects | TG, HDL-C, Adiponectin |
| Zhou NN [R84] | 2011 | Chinese | M | CAD patients | TG, TC, LDL-C, HDL-C, Adiponectin |
| Zhou NN [R84] | 2011 | Chinese | M | Healthy subjects | TG, TC, LDL-C, HDL-C, Adiponectin |
| Wu J [R85] | 2011 | Chinese | M/F | Obesity patients | TG, TC, LDL-C, HDL-C, Adiponectin |
| Prior SL [R86] | 2011 | Caucasian | M/F | T1DM patients | TC, LDL-C, HDL-C, Adiponectin |
| Wang DL [R87] | 2011 | Chinese | M/F | CAD patients and control subjects | TG, TC, LDL-C, HDL-C, Adiponectin |
| Min XH [R88] | 2011 | Chinese | F | Preeclampsia patients | TG, TC, LDL-C, HDL-C |
| Min Y [R89] | 2011 | Chinese | M/F | T2DM patients | TG, TC, HDL-C |
| Min Y [R89] | 2011 | Chinese | M/F | T2DM patients | TG, TC, HDL-C |
| Al Khaldi RM [R90] | 2011 | Middle eastern | M/F | Cancer patients and control subjects | Adiponectin |
| Park JW [R91] | 2011 | Korean | M/F | Healthy subjects | Adiponectin |
| Dhillon PK [R92] | 2011 | Caucasian | M | Prostate cancer patients | Adiponectin |
| Kang XL [R93] | 2012 | Chinese | M/F | Healthy subjects | TG, TC, LDL-C, HDL-C |
| Wang SJ [R94] | 2012 | Chinese | M/F | Mets patients | TG, HDL-C, Adiponectin |
| Wang SJ [R94] | 2012 | Chinese | M/F | Abnormal metabolism patients | TG, HDL-C, Adiponectin |
| Wang SJ [R94] | 2012 | Chinese | M/F | Healthy subjects | TG, HDL-C, Adiponectin |
| Li XX [R95] | 2012 | Chinese | M/F | Obesity patients | TG, TC, LDL-C, HDL-C |
| Lee JY [R96] | 2012 | Korean | M/F | Healthy subjects | TG, TC, HDL-C, Adiponectin |
| Curti ML [R97] | 2012 | Latino | M/F | Mets patients | TG, TC, LDL-C, HDL-C |
| Cao J [R98] | 2012 | Chinese | M/F | OSAHS patients | TG, TC, LDL-C, HDL-C, Adiponectin |
| Namvaran F [R99] | 2012 | Middle eastern | M/F | T2DM patients | TG, TC, LDL-C, HDL-C |
| Kang XL [R100] | 2012 | Chinese | M/F | T2DM patients | TG, TC, LDL-C, HDL-C |
| Kang XL [R100] | 2012 | Chinese | M/F | Healthy subjects | TG, TC, LDL-C, HDL-C |
| Chen XY [R101] | 2012 | Chinese | M/F | Obesity patients | TG, TC, LDL-C, HDL-C, Adiponectin |
| Chen XY [R101] | 2012 | Chinese | M/F | Healthy subjects | TG, TC, LDL-C, HDL-C, Adiponectin |
| Al-Daghri NM [R102] | 2012 | Middle eastern | M/F | Healthy subjects | TG, TC, LDL-C, HDL-C |
| Riestra P [R103] | 2012 | Caucasian | M | Healthy subjects | TG, TC, LDL-C, HDL-C, Adiponectin |
| Riestra P [R103] | 2012 | Caucasian | F | Healthy subjects | TG, TC, LDL-C, HDL-C, Adiponectin |
| Kacso IM [R104] | 2012 | Caucasian | M/F | T2DM patients | TG, LDL-C, HDL-C, Adiponectin |
| Kacso IM [R105] | 2012 | Caucasian | M/F | T2DM patients | TG, LDL-C, HDL-C, Adiponectin |
| Kacso IM [R105] | 2012 | Caucasian | M/F | Healthy subjects | TG, LDL-C, HDL-C, Adiponectin |
| Elshamaa MF [R106] | 2012 | Middle eastern | M/F | Chronic kidney disease patients | TG, TC, HDL-C |
| Tsuzaki K [R107] | 2012 | Japanese | M/F | Healthy subjects | TG, TC, HDL-C, Adiponectin |
| Ohara M [R108] | 2012 | Japanese | M/F | T2DM patients | TG, TC, LDL-C, HDL-C |
| Zheng HF [R109] | 2012 | Chinese | M/F | CAD patients and control subjects | TG, TC, LDL-C, HDL-C, Adiponectin |
| Xu J [R110] | 2012 | Chinese | M/F | T2DM patients | TG, TC, LDL-C, HDL-C |
| Oliveira CS [R111] | 2012 | Latino | M/F | CAD patients and control subjects | Adiponectin |
| Saito M [R112] | 2012 | Japanese | M/F | Healthy subjects | Adiponectin |
| Gui MH [R113] | 2012 | Chinese | M/F | CAD patients | Adiponectin |
| Gui MH [R113 | 2012 | Chinese | M/F | Healthy subjects | Adiponectin |
| Roszkowska-Gancarz M [R114] | 2012 | Caucasian | F | Healthy subjects | Adiponectin |
| Jochmanová I [R115] | 2013 | Caucasian | M/F | Primary aldosteronism patients | TG, TC |
| Jochmanová I [R115] | 2013 | Caucasian | M/F | Healthy subjects | TG, TC |
| Mackawy AM [R116] | 2013 | Middle eastern | F | Obesity patients | TG, TC, LDL-C, HDL-C, Adiponectin |
| Mackawy AM [R116] | 2013 | Middle eastern | F | Healthy subjects | TG, TC, LDL-C, HDL-C, Adiponectin |
| Lee KY [R117] | 2013 | Korean | F | Obesity patients | HDL-C, Adiponectin |
| Choe EY [R118] | 2013 | Korean | M/F | T2DM patients | TG, TC, LDL-C, HDL-C, Adiponectin |
| Galcheva SV [R119] | 2013 | Caucasian | M/F | Healthy subjects | TC, LDL-C, HDL-C, Adiponectin |
| Xu J [R120] | 2013 | Chinese | M/F | Mets patients and control subjects | TG, HDL-C |
| Arnaiz-Villena A [R121] | 2013 | Indian | M/F | Healthy subjects | TG, TC, HDL-C |
| Kang Z [R122] | 2013 | Chinese | M/F | T2DM patients | TG, TC, LDL-C, HDL-C |
| Kang Z [R122] | 2013 | Chinese | M/F | Healthy subjects | TG, TC, LDL-C, HDL-C |
| Gong QL [R123] | 2013 | Chinese | M/F | NAFLD patients and control subjects | TG, TC, LDL-C, HDL-C, Adiponectin |
| Kawai T [R124] | 2013 | Japanese | M/F | Hypertension patients | TG, TC, LDL-C, HDL-C |
| Hwang JY [R125] | 2013 | Korean | M/F | T2DM patients | TG, TC, LDL-C, HDL-C, Adiponectin |
| Hwang JY [R125] | 2013 | Korean | M/F | T2DM patients | TG, TC, LDL-C, HDL-C, Adiponectin |
| Hwang JY [R125] | 2013 | Korean | M/F | T2DM patients | TG, TC, LDL-C, HDL-C, Adiponectin |
| Wang CH [R126] | 2013 | Chinese | M/F | T2DM patients | TG, TC, LDL-C, HDL-C |
| Mente A [R127] | 2013 | Other ethnic | M/F | Healthy subjects | TG, LDL-C, HDL-C |
| Kang Z [R128] | 2013 | Chinese | M/F | Hypertension patients | TG, TC, LDL-C, HDL-C |
| Kang Z [R128] | 2013 | Chinese | M/F | Healthy subjects | TG, TC, LDL-C, HDL-C |
| Zandoná MR [R129] | 2013 | Latino | M/F | Healthy subjects | TG, TC, LDL-C, HDL-C |
| Ye Y [R130] | 2013 | Chinese | M/F | NAFLD patients | TG, TC, LDL-C, HDL-C |
| Ye Y [R130] | 2013 | Chinese | M/F | Healthy subjects | TG, TC, LDL-C, HDL-C |
| Wang LJ [R131] | 2013 | Chinese | M/F | Mets patients | TG, TC, LDL-C, HDL-C |
| Wang LJ [R131] | 2013 | Chinese | M/F | Healthy subjects | TG, TC, LDL-C, HDL-C |
| Yan CJ [R132] | 2013 | Chinese | M/F | Hypertension patients | Adiponectin |
| Yan CJ [R132] | 2013 | Chinese | M/F | Healthy subjects | Adiponectin |
| Chen LD [R133] | 2013 | Chinese | M/F | Cerebral infarction patients | Adiponectin |
| Ramya K [R134] | 2013 | Indian | M/F | T2DM patients | Adiponectin |
| Ramya K [R134] | 2013 | Indian | M/F | Healthy subjects | Adiponectin |
| Ramya K [R134] | 2013 | Indian | M/F | Obesity patients | Adiponectin |
| Ramya K [R134] | 2013 | Indian | M/F | Healthy subjects | Adiponectin |
| Sun ZL [R135] | 2014 | Chinese | M/F | T2DM patients | TG, TC, HDL-C |
| Sun ZL [R135] | 2014 | Chinese | M/F | Healthy subjects | TG, TC, HDL-C |
| Park JY [R136] | 2014 | Korean | M/F | Healthy subjects | TG, TC, LDL-C, HDL-C |
| Foucan L [R137] | 2014 | Caucasian | M/F | T2DM patients | TG |
| Sikka R [R138] | 2014 | Indian | M/F | T2DM patients | TG, TC, LDL-C, HDL-C |
| Shaker OG [R139] | 2014 | Middle eastern | M/F | CAD patients | TG, TC, HDL-C |
| Yang H [R140] | 2014 | Chinese | M/F | T2DM patients | TG, TC, LDL-C, HDL-C, Adiponectin |
| Zhang C [R141] | 2014 | Chinese | F | T2DM patients | TG, TC, LDL-C, HDL-C, Adiponectin |
| Su QJ [R142] | 2014 | Chinese | M/F | T2DM patients and control subjects | TG, TC, LDL-C, HDL-C |
| Yu XY [R143] | 2014 | Chinese | F | Preeclampsia patients | TG, TC, HDL-C |
| Li JQ [R144] | 2014 | Chinese | M/F | CAD patients | TG, TC, LDL-C, HDL-C |
| Chang JL [R145] | 2014 | Chinese | M/F | Obesity patients | TG, TC, LDL-C, HDL-C |
| Yu J [R146] | 2014 | Chinese | M/F | Obesity patients | TG, TC, LDL-C, HDL-C |
| Nikolajević-Starčević J [R147] | 2014 | Caucasian | M/F | T2DM patients | Adiponectin |
| Li Y [R148] | 2014 | Chinese | M/F | Hypertension patients and control subjects | Adiponectin |
| Gu CY [R149] | 2014 | Chinese | M | Prostate cancer patients | Adiponectin |
| Gu CY [R149] | 2014 | Chinese | M | Healthy subjects | Adiponectin |
| Su DY [R150] | 2015 | Chinese | M/F | T2DM patients | TG, TC, LDL-C, HDL-C, Adiponectin |
| Peng H [R151] | 2015 | Chinese | M/F | NAFLD patients | TG, TC, LDL-C, HDL-C |
| Wang CY [R152] | 2015 | Chinese | M/F | T2DM patients | TG, TC, LDL-C, HDL-C |
| Cheng YT [R153] | 2015 | Chinese | M/F | NAFLD patients and control subjects | TG, TC, LDL-C, HDL-C, Adiponectin |
| de Oliveira R [R154] | 2015 | Latino | M/F | Obesity patients and control subjects | TG, TC, LDL-C, HDL-C, Adiponectin |
| Tureck LV [R155] | 2015 | Latino | M | Healthy subjects | TG, TC, LDL-C, HDL-C, Adiponectin |
| Tureck LV [R155] | 2015 | Latino | F | Healthy subjects | TG, TC, LDL-C, HDL-C, Adiponectin |
| Sun Y [R156] | 2015 | Chinese | M/F | Mets patients | TG, HDL-C |
| Kaftan AN [R157] | 2015 | Middle eastern | M/F | T2DM patients | TG, TC, LDL-C, HDL-C |
| Motawi T [R158] | 2015 | Middle eastern | M | T2DM patients | Adiponectin |
| Motawi T [R158] | 2015 | Middle eastern | M | Healthy subjects | Adiponectin |
| Lanas F [R159] | 2015 | Other ethnic | M/F | Healthy subjects | Adiponectin |
| Low CF [R160] | 2015 | Other ethnic | F | GDM patients | Adiponectin |
| Low CF [R160] | 2015 | Other ethnic | F | Healthy subjects | Adiponectin |
| Han Y [R161] | 2015 | Chinese | F | GDM patients | Adiponectin |
| Han Y [R161] | 2015 | Chinese | F | Healthy subjects | Adiponectin |
| Wu HL [R162] | 2015 | Chinese | M/F | Mets patients and control subjects | Adiponectin |
| Wu HL [R162] | 2015 | Chinese | M/F | Mets patients and control subjects | Adiponectin |
| Klemettilä JP [R163] | 2015 | Caucasian | M/F | Schizophrenia patients | Adiponectin |
| Du SX [R164] | 2016 | Chinese | M/F | CAD and NAFLD patients | TG, TC, LDL-C, HDL-C, Adiponectin |
| Du SX [R164] | 2016 | Chinese | M/F | NAFLD patients | TG, TC, LDL-C, HDL-C, Adiponectin |
| Du SX [R164] | 2016 | Chinese | M/F | Healthy subjects | TG, TC, LDL-C, HDL-C, Adiponectin |
| Kato H [R165] | 2016 | Japanese | M/F | HIV patients | TG, TC |
| de Luis DA [R166] | 2016 | Caucasian | M/F | Obesity patients | TG, TC, LDL-C, HDL-C, Adiponectin |
| Hsiao TJ [R167] | 2016 | Chinese | M/F | T2DM patients and control subjects | TG, TC |
| Wang XX [R168] | 2016 | Chinese | F | GDM patients and control subjects | TG, TC, LDL-C, HDL-C |
| Yang GZ [R169] | 2016 | Chinese | M/F | T2DM patients | TG, TC, LDL-C, HDL-C |
| Nambiar V [R170] | 2016 | Indian | F | PCOS patients | Adiponectin |
| Al Hannan FA [R171] | 2016 | Middle eastern | M/F | T2DM patients | Adiponectin |
| Al Hannan FA [R171] | 2016 | Middle eastern | M/F | Healthy subjects | Adiponectin |
| Momin AA [R172] | 2017 | Indian | M/F | T2DM patients | TG, TC, LDL-C, HDL-C |
| Liu QQ [R173] | 2017 | Chinese | M/F | Obesity patients | TG, TC, LDL-C, HDL-C |
| Liu QQ [R173] | 2017 | Chinese | M/F | Healthy subjects | TG, TC, LDL-C, HDL-C |
| Zayani N [R174] | 2017 | Middle eastern | M/F | Obesity patients and control subjects | TC, LDL-C, HDL-C |
| de Luis DA [R175] | 2017 | Caucasian | M/F | Obesity patients | TG, TC, LDL-C, HDL-C, Adiponectin |
| Mohseni F [R176] | 2017 | Middle eastern | M/F | NAFLD patients | TC, LDL-C, HDL-C |
| Mohseni F [R176] | 2017 | Middle eastern | M/F | Healthy subjects | TC, LDL-C, HDL-C |
| Yang XN [R177] | 2017 | Chinese | M/F | T2DM patients | TG, TC, LDL-C, HDL-C, Adiponectin |
| Shi JK [R178] | 2017 | Chinese | M/F | Subarachnoid hemorrhages patients | Adiponectin |
| Shi JK [R178] | 2017 | Chinese | M/F | Healthy subjects | Adiponectin |
| Czeczuga-Semeniuk E [R179] | 2018 | Caucasian | F | PCOS patients | TG, TC, LDL-C, HDL-C |
| Ji MJ [R180] | 2018 | Korean | M/F | T2DM patients | TG, TC, LDL-C, HDL-C |
| Hussain MK [R181] | 2018 | Middle eastern | M/F | T2DM patients | TG, TC, LDL-C, HDL-C |
| Chang CS [R182] | 2018 | Chinese | M/F | Obesity patients | TG, TC, HDL-C, Adiponectin |
| Maistry T [R183] | 2018 | Indian | M/F | Mets patients and control subjects | TG, HDL-C |
| Kaur H [R184] | 2018 | Indian | M/F | Obesity patients and control subjects | TG, TC, LDL-C, HDL-C |
| Leońska-Duniec A [R185] | 2018 | Caucasian | F | Healthy subjects | TG, TC, LDL-C, HDL-C |
| de Luis DA [R186] | 2018 | Caucasian | M/F | Obesity patients | TG, TC, LDL-C, HDL-C, Adiponectin |
| de Luis DA [R186] | 2018 | Caucasian | M/F | Obesity patients | TG, TC, LDL-C, HDL-C, Adiponectin |
| de Luis DA [R187] | 2018 | Caucasian | M/F | Obesity patients | TG, TC, LDL-C, HDL-C, Adiponectin |
| Qian XS [R188] | 2018 | Chinese | M/F | CAD patients | TG, TC, LDL-C, HDL-C |
| Karimi H [R189] | 2018 | Middle eastern | M/F | T2DM patients and control subjects | TC, LDL-C, HDL-C |
| Cui M [R190] | 2018 | Chinese | M/F | T2DM patients | TG, TC, LDL-C, HDL-C |
| Khabour OF [R191] | 2018 | Middle eastern | F | Healthy subjects | Adiponectin |
| Barliana MI [R192] | 2019 | Other ethnic | M/F | Healthy subjects | TC, LDL-C, HDL-C |
| Barliana MI [R192] | 2019 | Other ethnic | M/F | Obesity patients | TC, LDL-C, HDL-C |
| Zheng WW [R193] | 2019 | Chinese | M/F | T2DM patients and control subjects | TC, LDL-C, HDL-C |
| Macías-Gómez NM [R194] | 2019 | Latino | F | Breast cancer patients | TG, TC, LDL-C, HDL-C |
| Macías-Gómez NM [R194] | 2019 | Latino | F | Healthy subjects | TG, TC, LDL-C, HDL-C |
| Ergören MC [R195] | 2019 | Caucasian | M/F | Obesity patients | TG, TC, LDL-C, HDL-C |
| Ergören MC [R195] | 2019 | Caucasian | M/F | Healthy subjects | TG, TC, LDL-C, HDL-C |
| Sánchez MP [R196] | 2019 | Latino | M/F | T2DM patients | TG, TC, LDL-C, HDL-C |
| Sánchez MP [R196] | 2019 | Latino | M/F | Healthy subjects | TG, TC, LDL-C, HDL-C |
| de Luis DA [R197] | 2019 | Caucasian | M/F | Obesity patients | TG, TC, LDL-C, HDL-C, Adiponectin |
| de Luis DA [R198] | 2019 | Caucasian | M/F | Obesity patients | TG, TC, LDL-C, HDL-C, Adiponectin |
| de Luis DA [R198] | 2019 | Caucasian | M/F | Obesity patients | TG, TC, LDL-C, HDL-C, Adiponectin |
| Aller R [R199] | 2019 | Caucasian | M/F | Obesity patients | TG, TC, LDL-C, HDL-C, Adiponectin |
| Aller R [R199] | 2019 | Caucasian | M/F | Obesity patients | TG, TC, LDL-C, HDL-C, Adiponectin |
| Nomani H [R200] | 2019 | Middle eastern | M/F | T1DM patients | TG, TC, LDL-C, HDL-C, Adiponectin |
| Nomani H [R200] | 2019 | Middle eastern | M/F | Healthy subjects | TG, TC, LDL-C, HDL-C, Adiponectin |
| Divella R [R201] | 2019 | Caucasian | M/F | Colorectal cancer patients | TG, HDL-C |
| de Luis DA [R202] | 2019 | Caucasian | M/F | Obesity patients | TG, TC, LDL-C, HDL-C, Adiponectin |
| Gumanova NG [R203] | 2019 | Caucasian | M | CAD patients | Adiponectin |
| Gumanova NG [R203] | 2019 | Caucasian | F | CAD patients | Adiponectin |
| Geriki S [R204] | 2019 | Indian | F | Breast cancer patients and control subjects | Adiponectin |
| Chuluun-Erdene A [R205] | 2020 | Other ethnic | M/F | Mets patients and control subjects | TC, LDL-C, HDL-C |
| Palit SP [R206] | 2020 | Indian | M/F | T2DM patients and control subjects | TG, TC, LDL-C, HDL-C |
| Garba M [R207] | 2020 | Middle eastern | M/F | Acne patients | TG, TC, LDL-C, HDL-C |
| Bains V [R208] | 2020 | Indian | M/F | T2DM patients | TG, TC, LDL-C, HDL-C |
| de Luis DA [R209] | 2020 | Caucasian | M/F | Obesity patients | TG, TC, LDL-C, HDL-C, Adiponectin |
| de Luis DA [R209] | 2020 | Caucasian | M/F | Obesity patients | TG, TC, LDL-C, HDL-C, Adiponectin |

M: male; F: female; CAD: coronary artery disease; T2DM: type 2 diabetes mellitus; PCOS: polycystic ovarian syndrome; IGT: impaired glucose tolerance; Mets: metabolic syndrome; NAFLD: nonalcoholic fatty liver disease; T1DM: type 1 diabetes mellitus; OSAHS: obstructive sleep apnea-hypopnea syndrome; GDM: gestational diabetes mellitus; HIV: Human Immunodeficiency Virus; TG: triglycerides; TC: total cholesterol; LDL-C: low-density lipoprotein cholesterol; HDL-C: high-density lipoprotein cholesterol

**Table S2.** Circulating lipids levels by the genotypes of the rs2241766 polymorphism.

| **First author, reference** | **Genotype** | |  | **TG (x±s), mmol/L** | |  | **TC (x±s), mmol/L** | |  | **LDL-C (x±s), mmol/L** | |  | **HDL-C (x±s), mmol/L** | |
| --- | --- | --- | --- | --- | --- | --- | --- | --- | --- | --- | --- | --- | --- | --- |
|  | **TT** | **TG+GG** |  | **TT** | **TG+GG** |  | **TT** | **TG+GG** |  | **TT** | **TG+GG** |  | **TT** | **TG+GG** |
| Schäffler A [R1] | 275 | 69 |  | 1.78±2.69 | 1.75±1.31 |  | 5.72±1.22 | 5.64±1.45 |  | 3.41±1.03 | 3.37±1.23 |  | 1.55±0.49 | 1.56±0.82 |
| Zietz B [R2] | 457 | 99 |  | 3.44±2.03 | 3.35±2.11 |  | 6.83±1.31 | 7.1±1.32 |  | 4.08±1.21 | 4.43±1.16 |  | 1.18±0.35 | 1.19±0.35 |
| Menzaghi C [R3] | 260 | 139 |  | 1.06±0.75 | 1.09±0.74 |  | 5.09±1.09 | 4.87± 0.91 |  | - | - |  | 1.37±0.34 | 1.37±0.30 |
| Yang WS [R4] | 91 | 154 |  | 1.44±0.86 | 1.4±0.10 |  | 4.66±0.85 | 4.62±1.03 |  | - | - |  | - | - |
| Filippi E [R5] | 189 | 61 |  | 1.49±0.82 | 1.72±1.06 |  | 5.59±1.05 | 5.68±1 |  | 3.56±0.87 | 3.47±1 |  | 1.48±0.39 | 1.46±0.39 |
| Stenvinkel P [R6] | 163 | 41 |  | 2.2±1.28 | 2.15±1.14 |  | 5.7 ±1.28 | 5.49±1.26 |  | - | - |  | 1.2±1.28 | 1.22±0.6 |
| Du PF [R7] | 95 | 71 |  | - | - |  | - | - |  | - |  |  | 1.2±1.0 | 1.4±1.0 |
| González-Sánchez JL [R11] | 472 | 275 |  | 1.13±0.71 | 1.12±0.63 |  | - | - |  | 5.51±0.98 | 5.51±1.03 |  | 1.55±0.44 | 1.55±0.47 |
| Xita N [R12] | 77 | 23 |  | 1.03±0.74 | 1.07±0.42 |  | 4.63±0.91 | 4.53±0.88 |  | 4.16±0.77 | 4.04±0.86 |  | 1.17±0.27 | 1.24±0.6 |
| Berthier MT [R13] | 193 | 52 |  | - | - |  | - | - |  | 3.43±0.76 | 3.42±0.62 |  | 0.24±0.07 | 0.24±0.06 |
| Nakatani K [R14] | 98 | 96 |  | 1.31±0.96 | 1.2±0.76 |  | - | - |  | - | - |  | 1.68±0.39 | 1.65±0.39 |
| Jang Y [R15] | 443 | 459 |  | - | - |  | 5.13±0.95 | 5.18± 1 |  | 3.23±0.86 | 3.25±0.89 |  | 1.22±0.32 | 1.22±0.3 |
| Lee YY [R16] | 201 | 226 |  | 1.54 ±0.80 | 1.59±0.80 |  | 5.15±0.87 | 5.17±0.89 |  | - | - |  | 1.14±0.32 | 1.18±0.33 |
| Kang ES [R17] | 86 | 80 |  | 2.02±1.15 | 2.38±1.58 |  | 4.88±0.85 | 5.15±1.05 |  | 2.75±0.78 | 2.83±0.74 |  | 1.23±0.27 | 1.2±0.31 |
| Ukkola O [R18] | 400 | 102 |  | 1.19±2.40 | 1.13±1.31 |  | 4.38±1.8 | 4.35±1.11 |  | - | - |  | 0.96±0.8 | 0.98±0.61 |
| Ukkola O [R18] | 245 | 27 |  | 0.91±2.03 | 1.06±1.04 |  | 4.26±1.57 | 4.78±0.83 |  | - | - |  | 1.06±1.1 | 1.07±0.52 |
| Haap M [R19] | 38 | 15 |  | 1.65±0.91 | 1.45±1.18 |  | 4.84±0.8 | 4.32±0.9 |  | - | - |  | - | - |
| Haap M [R19] | 414 | 128 |  | 1.19±0.92 | 1.17±0.64 |  | 4.99±1.05 | 5.02±0.88 |  | - | - |  | - | - |
| Shin MJ [R23] | 161 | 133 |  | 1.61±0.83 | 1.66±1.01 |  | 5.05±0.87 | 4.9±0.87 |  | 3.16±0.78 | 3.03±0.8 |  | 1.15±0.31 | 1.11±0.27 |
| Mousavinasab F [R24] | 226 | 26 |  | 0.88±0.50 | 0.93±0.50 |  | 3.7±0.7 | 3.8±0.6 |  | 2.18±0.60 | 2.3±0.6 |  | 1.14±0.30 | 1.03±0.2 |
| Yan WL [R25] | 222 | 275 |  | 1.20±0.6 | 1.22±0.83 |  | 4.77±1.0 | 4.76±0.9 |  | 2.90±0.8 | 2.86±0.8 |  | 1.31±0.3 | 1.33±0.31 |
| Petrone A [R26] | 208 | 62 |  | 1.07±0.59 | 1.08±0.48 |  | 4.3±0.78 | 4.18±0.83 |  | 2.56±0.64 | 2.67±0.72 |  | 1.28±0.31 | 1.3±0.32 |
| Tso AW [R27] | 137 | 125 |  | - | - |  | - | - |  | - | - |  | 1.26±0.34 | 1.15±0.28 |
| Wang JY [R33] | 84 | 115 |  | 2.14±1.92 | 2.03±1.79 |  | 5.14±1.15 | 5.06±1.11 |  | 2.99±0.87 | 3±0.72 |  | 1.37±0.35 | 1.36±0.36 |
| Wang JY [R33] | 65 | 73 |  | 1.26±0.72 | 1.54±1.05 |  | 4.73±0.84 | 4.79 ±0.9 |  | 2.77±0.64 | 2.85±0.71 |  | 1.5±0.31 | 1.44±0.28 |
| Katsuda Y [R37] | 34 | 30 |  | 1.72±1.17 | 1.71±0.93 |  | 5.46±0.91 | 5.28±1.11 |  | - | - |  | 1.19±0.26 | 1.16±0.34 |
| Jeng JR [R38] | 116 | 192 |  | 1.87±2.04 | 1.57±1.06 |  | 5.2±1.03 | 5.18±0.96 |  | - | - |  | - | - |
| Jeng JR [R38] | 92 | 168 |  | 1.46±1.41 | 1.36±0.85 |  | 5.46±1.09 | 5.38±0.99 |  | - | - |  | - | - |
| Zhang H [R41] | 57 | 46 |  | 2.14±1.81 | 2.16±1.63 |  | 5.28±1.06 | 5.24±1.15 |  | 3.27±0.91 | 3.23±1.02 |  | 1.25±0.30 | 1.18±0.20 |
| Li Z [R42] | 65 | 48 |  | 2.15±2.08 | 3.05±3.23 |  | 5.79±1.10 | 5.77 ± 1.03 |  | 3.45±0.90 | 3.35 ±0.78 |  | 1.35±0.33 | 1.25±0.30 |
| Sun H [R43] | 109 | 146 |  | 3.5±4.4 | 2.6±2.5 |  | 4.7±1.9 | 4.7±1.7 |  | 3.0±1.0 | 2.9±1.1 |  | 1.3±0.6 | 1.5±1.2 |
| Wang ZL [R44] | 146 | 118 |  | 1.91±1.24 | 2.33±1.29 |  | 5.24±0.92 | 5.24±1.04 |  | - | - |  | 1.13±0.30 | 1.12±0.45 |
| Wang ZL [R44] | 50 | 26 |  | 1.08±0.53 | 1.39±0.58 |  | 4.86±0.80 | 4.99±0.77 |  | - | - |  | 1.23±0.30 | 1.21±0.35 |
| Chen QY [R45] | 157 | 146 |  | 2.45 ±2.88 | 2.03±1.87 |  | 6.09±2.08 | 5.77±1.32 |  | 3.09 ±1.10 | 3.33±1.33 |  | 1.42±0.54 | 1.4±0.54 |
| Yang XJ [R46] | 88 | 67 |  | 4.67 ±1.18 | 4.76 ±1.10 |  | 1.83±1.29 | 1.63±0.92 |  | 2.73 ±0.96 | 2.79 ±0.95 |  | 1.24±0.45 | 1.27±0.36 |
| Yang XJ [R46] | 95 | 54 |  | 1.48 ±0.49 | 1.55 ±0.45 |  | 4.39±1.19 | 4.47±1.21 |  | 2.46 ±0.88 | 2.44 ±0.84 |  | 1.45±0.43 | 1.44±0.29 |
| Ai ZH [R47] | 89 | 87 |  | 1.74±1.43 | 1.88±2.64 |  | 4.42±0.83 | 4.46±0.98 |  | 2.43±0.64 | 2.54±0.79 |  | 1.29±0.30 | 1.31±0.30 |
| Pérez-Martínez P [R48] | 45 | 14 |  | 0.73±0.29 | 0.74±0.36 |  | 4.16±0.60 | 4.31±0.64 |  | 2.55±0.56 | 2.71±0.55 |  | 1.27±0.25 | 1.26±0.34 |
| Jang Y [R49] | 438 | 414 |  | 1.34±0.71 | 1.33±0.62 |  | - | - |  | - | - |  | - | - |
| Potapov VA [R50] | 117 | 12 |  | 2.2±0.6 | 2.3±0.6 |  | 4.9±1.3 | 5.2±1.2 |  | 3.2±1.1 | 3.5±1.2 |  | 1.3±0.3 | 1.3±0.4 |
| Potapov VA [R50] | 108 | 9 |  | 1.6±0.5 | 1.7±0.5 |  | 4.9±1.1 | 5.2±1.1 |  | 3.3±0.9 | 3.6±1.0 |  | 1.2±0.3 | 1.2±0.3 |
| Musso G [R51] | 58 | 12 |  | 1.14±0.69 | 1.02±0.43 |  | 4.65±0.98 | 4.73±1.16 |  | 2.97±1.18 | 2.92±0.81 |  | 1.19±0.2 | 1.32±0.18 |
| Musso G [R51] | 41 | 29 |  | 0.87±0.43 | 0.89±0.36 |  | 4.5±1.49 | 4.37±1.11 |  | 2.64±1.32 | 2.56±1.11 |  | 1.53±0.17 | 1.6±0.14 |
| Kim SH [R52] | 346 | 362 |  | 1.70±1.13 | 1.71±1.08 |  | 4.62±0.89 | 4.62±0.82 |  | 2.54±0.82 | 2.52±0.75 |  | 1.31±0.30 | 1.32±0.32 |
| Mohammadzadeh G [R58] | 26 | 24 |  | 2.14±0.59 | 2.17±0.75 |  | 4.84±1.22 | 4.8±0.15 |  | 2.96±1.16 | 2.88±1.14 |  | 0.91±0.16 | 0.88±0.13 |
| Mohammadzadeh G [R58] | 25 | 27 |  | 2.02±0.55 | 1.88±0.41 |  | 5.32±1.37 | 5.44±1.57 |  | 4.32±1.65 | 3.56±1.41 |  | 0.95±0.17 | 1.02±0.22 |
| Shu F [R59] | 61 | 64 |  | 1.74±1.06 | 1.87±1.18 |  | 4.68±1.62 | 4.24±1.21 |  | 2.28±1.15 | 2.67±0.85 |  | 1.35±0.40 | 1.32±0.49 |
| Shu F [R59] | 28 | 31 |  | 1.67±1.14 | 1.57±0.82 |  | 4.31±1.07 | 4.48±1.44 |  | 2.40±0.71 | 2.50±1.15 |  | 1.41±0.57 | 1.39±0.31 |
| Yoshihara K [R60] | 31 | 28 |  | 1.3±0.8 | 1.49±0.84 |  | 5.13±1 | 5.17±0.92 |  | 3.12±0.7 | 3.13±0.83 |  | 1.47±0.41 | 1.42±0.46 |
| Tsuzaki K [R61] | 20 | 12 |  | 1.25±0.86 | 0.8±0.34 |  | 5.56±0.88 | 5.84±1.11 |  | 3.03±0.7 | 3.41±0.78 |  | 1.66±0.39 | 1.73±0.41 |
| Melistas L [R62] | 249 | 93 |  | 1.08±0.54 | 1.07±0.55 |  | 5.66±1.12 | 5.68±1.16 |  | 3.82±0.92 | 3.85±0.94 |  | 1.34±0.29 | 1.35±0.27 |
| Panagopoulou P [R63] | 33 | 15 |  | 1.13±0.69 | 0.9±0.69 |  | 3.93±0.83 | 4.11±0.59 |  | 2.43±0.78 | 2.77±0.8 |  | 0.96±0.21 | 1.03±0.18 |
| Sone Y [R74] | 84 | 64 |  | 1.71±1.08 | 2.11±1.42 |  | 5.41±0.99 | 5.74±0.92 |  | 3.26±0.85 | 3.35±0.96 |  | 1.37±0.29 | 1.42±0.33 |
| Ronconi V [R75] | 63 | 26 |  | 1.54±1.02 | 1.54±0.85 |  | - | - |  | - | - |  | 1.23±0.39 | 1.21±0.28 |
| Ronconi V [R75] | 88 | 47 |  | 1.33±0.9 | 1.42±0.78 |  | - | - |  | - | - |  | 1.19±0.32 | 1.24±0.42 |
| Xu L [R76] | 78 | 75 |  | 1.91±1.58 | 1.58 ±1.04 |  | 4.95±1.36 | 4.89 ±1.21 |  | 2.95±1.05 | 2.91±0.91 |  | 1.01±0.32 | 1.08 ±0.27 |
| Xu L [R76] | 50 | 23 |  | 1.36 ±0.55 | 1.42 ±0.66 |  | 4.92±0.86 | 4.79±0.84 |  | 2.74±0.54 | 2.75±0.63 |  | 1.29 ±0.18 | 1.20 ±0.25 |
| Li YP [R77] | 59 | 74 |  | 2.5±1.8 | 2.52±1.58 |  | 4.9±1.3 | 4.6±1.09 |  | 2.4±0.9 | 2.34±0.72 |  | 1.1±0.2 | 1.26±0.4 |
| Li YP [R77] | 24 | 33 |  | 1.1±0.7 | 1.29±0.61 |  | 4.2±0.7 | 4.17±0.62 |  | 2.3±0.6 | 2.29±0.5 |  | 1.4±0.3 | 1.3±0.31 |
| Liang YL [R84] | 30 | 48 |  | - | - |  | 4.28±0.97 | 4.59±1.08 |  | - | - |  | - | - |
| Yang HY [R85] | 64 | 56 |  | 2.10 ±1.66 | 2.27±1.6 |  | 4.88±0.99 | 4.91 ± 0.78 |  | 2.91±0.78 | 2.88±0.99 |  | 1.19±0.30 | 1.04±1.32 |
| Zhou Q [R86] | 43 | 47 |  | - | - |  | 5.47±0.13 | 5.7±0.2 |  | - | - |  | - | - |
| Zhou Q [R86] | 31 | 47 |  | - | - |  | 5.13±0.17 | 5.16±0.14 |  | - | - |  | - | - |
| Leu HB [R87] | 477 | 485 |  | 1.72±3.44 | 1.86±1.56 |  | - | - |  | - | - |  | 1.27±0.88 | 1.28±0.31 |
| Boumaiza I [R88] | 209 | 120 |  | 1.1±0.6 | 1.07±0.58 |  | 5.0 ±1.2 | 4.83±1.09 |  | 3.3±0.9 | 3.09±3.63 |  | 1.2±0.4 | 1.21±0.39 |
| Zhou NN [R89] | 175 | 183 |  | 2.19±0.78 | 2.21±0.85 |  | 4.65±0.71 | 4.84±0.68 |  | 2.38±0.70 | 2.59±0.74 |  | 1.45±0.22 | 1.44±0.24 |
| Zhou NN [R89] | 42 | 23 |  | 2.14±0.81 | 1.98±0.75 |  | 4.49±0.63 | 4.65±0.64 |  | 2.26±0.63 | 2.41±0.77 |  | 1.37±0.26 | 1.38±0.27 |
| Wu J [R90] | 47 | 100 |  | 1.4 ± 0.6 | 1.12±0.3 |  | 3.7±0.5 | 4.12±0.52 |  | 1.96±0.36 | 2.28±0.42 |  | 1.32±0.43 | 1.24±0.23 |
| Kang XL [R98] | 135 | 54 |  | 1.32±0.24 | 1.43±0.28 |  | 4.38±0.40 | 4.68±0.43 |  | 2.69±0.34 | 3.10±0.40 |  | 1.25±0.23 | 1.15±0.21 |
| Wang SJ [R99] | 90 | 90 |  | 2.29±1.61 | 2.45±1.71 |  | - | - |  | - | - |  | 1.12±0.28 | 1.15±0.19 |
| Wang SJ [R99] | 98 | 66 |  | 1.64±0.89 | 1.55±0.73 |  | - | - |  | - | - |  | 1.22±0.30 | 1.23±0.24 |
| Wang SJ [R99] | 31 | 19 |  | 1.1±0.33 | 1.05±0.39 |  | - | - |  | - | - |  | 1.41±0.28 | 1.33±0.26 |
| Li XX [R100] | 33 | 21 |  | 1.69±1.11 | 1.69±1.11 |  | 4.57±0.68 | 4.68±0.60 |  | 3.36±0.56 | 3.6±0.86 |  | 1.32±0.14 | 1.37±0.18 |
| Lee JY [R101] | 511 | 514 |  | 1.28±0.66 | 1.16±0.55 |  | 4.73±0.88 | 4.67±0.8 |  | - | - |  | 1.16±0.33 | 1.19±0.32 |
| Curti ML [R102] | 94 | 37 |  | 1.76±0.74 | 1.77±0.77 |  | 5.21±1.15 | 5.11±1.1 |  | 3.28±1.03 | 3.24±1.08 |  | 1.09±0.32 | 1.1±0.31 |
| Cao J [R103] | 39 | 39 |  | 2.58±4.15 | 1.77±0.90 |  | 4.19±0.96 | 3.96±0.71 |  | 3.05±0.85 | 2.92±0.64 |  | 1.03±0.22 | 1.00±0.24 |
| Namvaran F [R104] | 67 | 34 |  | 1.84±0.97 | 2±1.07 |  | 4.26±1 | 4.38±0.99 |  | 2.44±0.75 | 2.32±0.73 |  | 1.09±0.26 | 1.14±0.22 |
| Kang XL [R105] | 219 | 113 |  | 2.53±0.39 | 2.71±0.42 |  | 4.79±1.09 | 5.22±1.17 |  | 2.47±0.47 | 2.92±0.47 |  | 1.25±0.28 | 1.27±0.32 |
| Kang XL [R105] | 235 | 72 |  | 1.77±0.46 | 1.95±0.48 |  | 3.98±1.13 | 4.85±1.24 |  | 2.11±0.78 | 2.43±1.12 |  | 1.35±0.25 | 1.43±0.28 |
| Chen XY [R106] | 96 | 152 |  | 1.16±1.02 | 1.96±1.03 |  | 5.24±0.91 | 5.25±1.13 |  | 3.23±1.18 | 4.13±1.28 |  | 1.37±0.25 | 1.01±0.28 |
| Chen XY [R106] | 125 | 98 |  | 1.49±0.29 | 1.37±0.22 |  | 4.71±0.9 | 4.3±0.81 |  | 2.96±0.72 | 3.17±0.92 |  | 1.46±0.35 | 1.39±0.33 |
| Al-Daghri NM [R107] | 209 | 89 |  | 1.4± 0.1 | 1.39±0.08 |  | 5.1±1.0 | 5.07±1.04 |  | 3.6±0.93 | 3.68±0.89 |  | 0.69±0.03 | 0.61±0.03 |
| Jochmanová I [R120] | 41 | 6 |  | 1.56±1.00 | 1.44±0.37 |  | 4.60±0.86 | 5.25±1.22 |  | - | - |  | - | - |
| Jochmanová I [R120] | 69 | 21 |  | 1.16±0.48 | 1.18±0.44 |  | 4.88±1.08 | 4.95±1.08 |  | - | - |  | - | - |
| Mackawy AM [R121] | 40 | 14 |  | 1.37±0.09 | 1.48±0.08 |  | 5.67±0.49 | 5.98±0.36 |  | 3.68±0.6 | 3.7±0.19 |  | 1.3±0.11 | 1.28±0.08 |
| Mackawy AM [R121] | 35 | 11 |  | 1.3±0.13 | 1.25±0.16 |  | 5.32±0.41 | 5.51±0.29 |  | 3.6±0.26 | 3.58±0.27 |  | 1.33±0.16 | 1.34±0.09 |
| Lee KY [R122] | 36 | 54 |  | - | - |  | - | - |  | - | - |  | 1.35±0.3 | 1.28±0.31 |
| Choe EY [R123] | 346 | 362 |  | 1.7±1.1 | 1.73±1.89 |  | 4.6± 0.9 | 4.6±0.81 |  | 2.5± 0.8 | 2.48± 0.7 |  | 1.3±0.3 | 1.3±0.39 |
| Galcheva SV [R124] | 129 | 38 |  | - | - |  | 4.3±0.7 | 4.3±0.8 |  | 2.61±0.57 | 2.49±0.71 |  | 1.25±0.29 | 1.36±0.32 |
| Xu J [R125] | 465 | 442 |  | 1.46 ±0.73 | 1. 46 ±0. 84 |  | - | - |  | - | - |  | 1.26 ± 0.31 | 1.26 ± 0.32 |
| Arnaiz-Villena A [R126] | 236 | 85 |  | 2.71±1.94 | 2.35±1.39 |  | 5.62±1.43 | 5.45±1.47 |  | - | - |  | 1.22±0.37 | 1.17±0.33 |
| Kang Z [R127] | 75 | 105 |  | 2.7±1.3 | 2.4±1.69 |  | 5.1±1.6 | 5.68±1.32 |  | 3.9±1.1 | 4.05±1.22 |  | 1.3±0.1 | 1.12±0.19 |
| Kang Z [R127] | 64 | 56 |  | 1.9±1.1 | 2.27±0.57 |  | 4.9 ± 1.3 | 5.27±1.04 |  | 2.90±1.2 | 2.63±0.44 |  | 1.3±0.1 | 0.93±0.21 |
| Gong QL [R128] | 358 | 302 |  | 1.71±1.06 | 1.78±1.22 |  | 5.3±1.0 | 5.23±1.01 |  | 2.82±1.41 | 2.69±1.12 |  | 1.81±0.35 | 1.79±0.34 |
| Sun ZL [R143] | 21 | 29 |  | 2.54±1.36 | 2.53±1.37 |  | 5.24±1.01 | 4.77±1.25 |  | - | - |  | 1.59±0.4 | 1.43±0.49 |
| Sun ZL [R143] | 19 | 21 |  | 1.78±0.86 | 2.2±1.19 |  | 4.41±0.34 | 4.83±0.77 |  | - | - |  | 1.42±0.29 | 1.53±0.35 |
| Park JY [R144] | 324 | 360 |  | 0.75±0.39 | 0.74±0.39 |  | 4.34±0.7 | 4.36±0.68 |  | 2.49±0.63 | 2.5±0.61 |  | 1.5±0.31 | 1.51±0.3 |
| Foucan L [R145] | 169 | 23 |  | 1.1±0.7 | 1.7±1.8 |  | - | - |  | - | - |  | - | - |
| Sikka R [R146] | 410 | 56 |  | 2.05±1.16 | 2.88±1.88 |  | 4.05±1.23 | 4.3±1.35 |  | 1.94±1.14 | 1.92±1.1 |  | 1.16±0.31 | 1.06±0.31 |
| Shaker OG [R147] | 44 | 16 |  | 2.14±0.48 | 2.06±0.13 |  | 6.4±0.86 | 6.54±1.02 |  | - | - |  | 0.81±0.14 | 0.77±0.16 |
| Yang H [R148] | 63 | 17 |  | 1.84±0.95 | 2.14±0.90 |  | 4.65±0.85 | 5.02±0.91 |  | 2.84±0.83 | 2.99±0.69 |  | 1.29±0.29 | 1.26±0.31 |
| Zhang C [R149] | 25 | 22 |  | 1.34±0.54 | 3.65±0.93 |  | 4.93±0.82 | 5.17±1.33 |  | 3.27±0.47 | 3.71±0.85 |  | - | - |
| Su QJ [R150] | 155 | 158 |  | 2.11±1.67 | 1.96±1.75 |  | 5.90±1.19 | 5.9±1.19 |  | 3.31±1.14 | 3.36±0.92 |  | 1.44±0.48 | 1.39±0.41 |
| Yu XY [R151] | 96 | 121 |  | 3.96±1.99 | 3.92±1.84 |  | 6.55±1.94 | 6.97±1.99 |  | - | - |  | 1.83±0.49 | 1.98±0.52 |
| Su DY [R159] | 49 | 60 |  | 2.28±0.63 | 2.94±0.7 |  | 5.03±1.07 | 5.86±1.4 |  | 2.79±0.67 | 3.39±0.91 |  | 0.96±0.25 | 0.78±0.21 |
| Peng H [R160] | 46 | 54 |  | 2.00±1.50 | 2.07±1.66 |  | 4.13±1.38 | 4.37±0.79 |  | 3.07±1.69 | 2.85±0.87 |  | 0.94±0.26 | 1.01±0.3 |
| Wang CY [R161] | 52 | 45 |  | 2.88±1.47 | 2.92±1.51 |  | 5.18±0.45 | 5.24±0.66 |  | 3.29±0.25 | 3.26±0.34 |  | 1.26±0.36 | 1.29±0.43 |
| Cheng YT [R162] | 313 | 305 |  | 1.54±1.52 | 1.74±1.8 |  | 4.7±0.93 | 4.71±0.97 |  | 3.06±0.86 | 3.24±0.95 |  | 1.41±0.42 | 1.33±0.40 |
| de Oliveira R [R163] | 163 | 86 |  | 1.4±0.79 | 1.52±1.05 |  | 5.3±0.93 | 5.28±1.11 |  | 3.23±0.83 | 3.21±0.88 |  | 1.42±0.39 | 1.37±0.41 |
| Du SX [R174] | 126 | 120 |  | 2.68±0.96 | 2.83±1.00 |  | 5.36±1.01 | 5.67±1.02 |  | 3.21±0.71 | 3.29±0.97 |  | 1.41±0.41 | 1.43±0.39 |
| Du SX [R174] | 127 | 120 |  | 2.17±0.69 | 2.21 ± 0.76 |  | 5.04±0.69 | 5.04±0.84 |  | 2.61±0.67 | 2.59±0.65 |  | 1.35±0.32 | 1.32±0.25 |
| Du SX [R174] | 185 | 119 |  | 1.36 ±0.41 | 1.29 ± 0.33 |  | 4.28±0.52 | 4.01±0.36 |  | 2.60±0.68 | 2.61±0.76 |  | 1.54±0.42 | 1.54±0.42 |
| Kato H [R175] | 22 | 56 |  | 1.52±0.74 | 1.75±0.63 |  | 4.14±0.88 | 3.95±0.93 |  | - | - |  | - | - |
| Momin AA [R182] | 127 | 23 |  | 1.79±0.24 | 2.17±0.2 |  | 5.25±0.79 | 5.54±0.86 |  | 3.29±0.76 | 3.49±0.82 |  | 1.14±0.15 | 1.06±0.11 |
| Liu QQ [R183] | 30 | 70 |  | 1.09±0.17 | 1.18±0.23 |  | 4.16±0.26 | 4.29±0.28 |  | 2.28±0.19 | 2.41±0.26 |  | 1.29±0.20 | 1.21±0.18 |
| Liu QQ [R183] | 54 | 46 |  | 0.80±0.17 | 0.82±0.16 |  | 3.97±0.22 | 3.86±0.16 |  | 2.07±0.20 | 2.10±0.19 |  | 1.43±0.15 | 1.40±0.18 |
| Zayani N [R184] | 694 | 427 |  | - | - |  | 4.92±0.99 | 4.88±0.96 |  | 3.31±0.80 | 3.3±0.83 |  | 1.16±0.35 | 1.12±0.34 |
| Czeczuga-Semeniuk E [R189] | 254 | 38 |  | 1.05±0.67 | 0.85±0.5 |  | 4.42±0.77 | 4.41±0.81 |  | 2.56±0.68 | 2.48±0.58 |  | 1.42±0.42 | 1.53±0.58 |
| Ji MJ [R190] | 410 | 348 |  | 1.75 ±0.99 | 1.81 ± 1.31 |  | 4.44±0.88 | 4.25±0.83 |  | 2.55±0.73 | 2.45±0.75 |  | 1.22±0.32 | 1.13±0.29 |
| Hussain MK [R191] | 257 | 143 |  | 2.71±0.34 | 2.81±0.36 |  | 6.39±1.17 | 6.68±1.3 |  | 3.92±1.55 | 4.25±1.38 |  | 1.23±0.34 | 1.15±0.37 |
| Chang CS [R192] | 118 | 116 |  | 2.07±0.23 | 2.05±0.22 |  | 4.95±1.04 | 4.93±1 |  | - | - |  | 1.15±0.29 | 1.17±0.24 |
| Maistry T [R193] | 745 | 254 |  | 1.8±1.2 | 1.86±1.19 |  | - | - |  | - | - |  | 1.4±0.7 | 1.57±5.01 |
| Barliana MI [R202] | 27 | 26 |  | - | - |  | 6.37±0.5 | 5.83±0.59 |  | 3.48±0.6 | 3.71±0.58 |  | 0.86±0.1 | 0.87±0.14 |
| Barliana MI [R202] | 32 | 22 |  | - | - |  | 5.51±0.3 | 5.71±0.22 |  | 3.26±1.05 | 2.95±0.3 |  | 0.88±0.1 | 0.93±0.1 |
| Zheng WW [R203] | 452 | 372 |  | - | - |  | 4.91±0.91 | 4.92±1.01 |  | 2.80±0.76 | 2.74±0.81 |  | 1.21±0.29 | 1.24±0.30 |
| Macías-Gómez NM [R204] | 55 | 9 |  | 2.59±3.08 | 2.59±3.08 |  | 5.38±1.58 | 7.4±1.6 |  | 3.36±1.06 | 3.36±1.06 |  | 1.24±0.08 | 1.24±0.44 |
| Macías-Gómez NM [R204] | 115 | 46 |  | 2.59±1.87 | 2.41±1.91 |  | 5.82±1.45 | 5.64±1.42 |  | 3.67±1.5 | 3.21±1.6 |  | 1.32±0.54 | 1.24±0.47 |
| Ergören MC [R205] | 60 | 40 |  | 1.98±1.09 | 1.67±0.66 |  | 5.93±0.86 | 6.48±1.37 |  | 3.74±0.72 | 4.22±1.45 |  | 1.21±0.29 | 1.27±0.83 |
| Ergören MC [R205] | 56 | 44 |  | 1.15±0.55 | 1.5±0.82 |  | 5.25±0.72 | 5.2±0.5 |  | 3.29±0.69 | 3.5±0.79 |  | 1.51±0.18 | 1.27±0.64 |
| Sánchez MP [R206] | 41 | 5 |  | 2.45±0.67 | 1.85±0.91 |  | 5.74±1.18 | 4.58±0.56 |  | 3.58±1.02 | 2.83±0.59 |  | 1.01±0.17 | 0.83±0.04 |
| Sánchez MP [R206] | 30 | 14 |  | 0.68±0.21 | 0.64±0.19 |  | 4.24±0.57 | 4.06±0.53 |  | 2.43±0.56 | 2.38±0.31 |  | 1.37±0.26 | 1.34±0.13 |
| Chuluun-Erdene A [R215] | 157 | 147 |  | - | - |  | 3.87±0.98 | 4.05±0.92 |  | 2.51±1.06 | 2.47±1 |  | 0.92±0.37 | 0.84±0.33 |
| Palit SP [R216] | 649 | 177 |  | 1.85±0.17 | 1.9±0.29 |  | 4.24±0.96 | 4.28±1.14 |  | 2.48±1.02 | 2.5±1.01 |  | - | - |
| Garba M [R217] | 171 | 59 |  | 0.99±0.57 | 1.00±0.66 |  | 4.22±0.85 | 4.03±1.08 |  | 2.35±0.70 | 2.32±0.67 |  | 1.44±0.377 | 1.42±0.315 |

TG: triglycerides; TC: total cholesterol; LDL-C: low-density lipoprotein cholesterol; HDL-C: high-density lipoprotein cholesterol

**Table S3.** Circulating lipids levels by the genotypes of the rs1501299 polymorphism.

| **First author, reference** | **Genotype** | |  | **TG (x±s), mmol/L** | |  | **TC (x±s), mmol/L** | |  | **LDL-C (x±s), mmol/L** | |  | **HDL-C (x±s), mmol/L** | |
| --- | --- | --- | --- | --- | --- | --- | --- | --- | --- | --- | --- | --- | --- | --- |
|  | **GG** | **GT+TT** |  | **GG** | **GT+TT** |  | **GG** | **GT+TT** |  | **GG** | **GT+TT** |  | **GG** | **GT+TT** |
| Stenvinkel P [R5] | 100 | 104 |  | 2.3±1 | 2.08±0.91 |  | 5.7±2 | 5.54±1.11 |  | - | - |  | 1.2±1 | 1.18±0.85 |
| Du PF [R6] | 15 | 151 |  | - | - |  | - | - |  | - | - |  | 1.5±1.1 | 1.24±1.0 |
| Yoshioka K [R7] | 170 | 176 |  | 1.55±1.1 | 1.54±0.98 |  | 5.4±0.91 | 5.24±0.98 |  | - | - |  | 1.54±0.39 | 1.42±0.35 |
| Berthier MT [R12] | 109 | 107 |  | - | - |  | - | - |  | 3.28 ± 0.77 | 3.52 ± 0.71 |  | 0.25±0.07 | 0.22±0.05 |
| Nakatani K [R13] | 80 | 114 |  | 1.21±0.68 | 1.29±0.98 |  | - | - |  | - | - |  | 1.64±0.36 | 1.67±0.41 |
| Jang Y [R14] | 451 | 451 |  | - | - |  | 5.19±0.94 | 5.12±1.01 |  | 3.25±0.85 | 3.23±0.91 |  | 1.21±0.28 | 1.23±0.33 |
| Lee YY [R15] | 225 | 202 |  | 1.57±0.79 | 1.57± 0.82 |  | 5.57±0.90 | 5.09±0.84 |  | - | - |  | 1.16±0.31 | 1.15±0.34 |
| Kang ES [R16] | 91 | 75 |  | 2.15±1.48 | 2.22±1.23 |  | 5.05±0.99 | 4.95±0.91 |  | 2.82 ± 0.67 | 2.74± 0.87 |  | 1.19±0.27 | 1.24±0.31 |
| Shin MJ [R22] | 145 | 149 |  | 1.78±0.99 | 1.5±0.82 |  | 4.95±0.91 | 5.03±0.84 |  | 3.05±0.83 | 3.17±0.76 |  | 1.1±0.27 | 1.17±0.31 |
| Mousavinasab F [R23] | 137 | 115 |  | 0.9±0.6 | 0.86±0.42 |  | 3.8±0.7 | 3.72±0.6 |  | 2.2±0.6 | 2.2±0.58 |  | 1.1±0.3 | 1.1±0.3 |
| Petrone A [R25] | 162 | 108 |  | 1.13±0.48 | 1.16±0.51 |  | 4.31±0.95 | 4.25±0.76 |  | 2.58±0.67 | 2.55±0.68 |  | 1.29±0.33 | 1.3±0.3 |
| Iacobellis G [R27] | 23 | 39 |  | 0.94±0.27 | 0.99±0.28 |  | 4.89±0.58 | 4.69±0.58 |  | - | - |  | 1.36±0.35 | 1.34±0.26 |
| Wang JY [R31] | 109 | 91 |  | 2.06±1.93 | 2.10±1.73 |  | 4.97±1.17 | 5.23±1.07 |  | 2.91±0.76 | 3.09±0.8 |  | 1.35±0.36 | 1.39±0.34 |
| Wang JY [R31] | 83 | 55 |  | 1.42±0.98 | 1.39±0.81 |  | 4.81±0.96 | 4.69±0.73 |  | 2.87±0.74 | 2.72±0.57 |  | 1.51±0.3 | 1.42±0.28 |
| Yu SY [R32] | 98 | 93 |  | 1.44±0.89 | 1.56±0.82 |  | 4.81±1.04 | 4.63±0.86 |  | 2.91±0.88 | 2.65±0.8 |  | 1.27±0.37 | 1.24±0.38 |
| Yu SY [R32] | 69 | 91 |  | 1.5±0.76 | 1.63±1.18 |  | 5.14±0.97 | 5.16±1.12 |  | 2.99±0.91 | 3.1±1 |  | 1.4±0.43 | 1.38±0.37 |
| Wang ZL [R42] | 113 | 151 |  | 2.24±1.28 | 2.04±1.21 |  | 5.23±0.93 | 5.14±0.94 |  | - | - |  | 1.13±0.46 | 1.14±0.39 |
| Wang ZL [R42] | 28 | 48 |  | 1.09±0.63 | 0.96±0.31 |  | 4.48±0.56 | 4.66±0.54 |  | - | - |  | 1.24±0.36 | 1.25±0.39 |
| Yang XJ [R44] | 70 | 85 |  | 1.54±0.74 | 1.92±1.38 |  | 4.60±1.13 | 4.8±1.15 |  | 2.72 ±0.97 | 2.78±0.95 |  | 1.22 ±0.35 | 1.28±0.45 |
| Yang XJ [R44] | 79 | 70 |  | 1.46±0.50 | 1.56±0.44 |  | 4.32±1.20 | 4.52±1.18 |  | 2.43 ±0.84 | 2.49±0.89 |  | 1.45 ±0.44 | 1.44±0.31 |
| Pérez-Martínez P [R46] | 31 | 28 |  | 0.78±0.32 | 0.68±0.30 |  | 4.25±0.60 | 4.12±0.63 |  | 2.66 ± 0.54 | 2.51 ± 0.60 |  | 1.22±0.31 | 1.29±0.23 |
| Jang Y [R47] | 416 | 436 |  | 1.39±0.69 | 1.29±0.62 |  | - | - |  | - | - |  | - | - |
| Musso G [R49] | 17 | 53 |  | 0.94±0.47 | 1.15±0.66 |  | 4.65±0.75 | 4.68±1.13 |  | 3.08±0.53 | 2.95±1.13 |  | 1.42±0.21 | 1.27±0.19 |
| Musso G [R49] | 38 | 32 |  | 0.86±0.35 | 0.9±0.38 |  | 4.32±1.43 | 4.5±1.17 |  | 2.69±1.28 | 2.74±0.88 |  | 1.6±0.16 | 1.5±0.15 |
| Kim SH [R50] | 351 | 357 |  | 1.78±1.20 | 1.63±1.00 |  | 4.65±0.88 | 4.59±0.82 |  | 2.52±0.81 | 2.54±0.75 |  | 1.32±0.31 | 1.3±0.30 |
| Guo ZX [R51] | 70 | 38 |  | 1.43±0.84 | 1.44±0.89 |  | 4.82±1.37 | 4.74±0.91 |  | -- | - |  | - | - |
| Guo ZX [R51] | 50 | 28 |  | 1.63±0.35 | 2.05±0.92 |  | 4.39±1.25 | 5.01±1.72 |  | - | - |  | - | - |
| Guo ZX [R51] | 35 | 41 |  | 1.50±0.60 | 1.54±0.81 |  | 4.76±0.78 | 4.54±0.77 |  | - | - |  | - | - |
| Mohammadzadeh G [R56] | 29 | 21 |  | 1.95±0.42 | 0.24±0.80 |  | 4.73±1.11 | 3.08±1.49 |  | 2.92±1.04 | 2.92±1.29 |  | 0.89±0.14 | 0.9±0.17 |
| Mohammadzadeh G [R56] | 27 | 25 |  | 2.09±0.6 | 0.75±0.41 |  | 5.41±1.22 | 5.27±1.58 |  | 4.86±2.27 | 3.42±1.47 |  | 0.93±0.17 | 1±0.19 |
| Yoshihara K [R58] | 34 | 25 |  | 1.19±0.8 | 1.67±0.99 |  | 5.12±0.8 | 5.34±1.29 |  | 3.1±0.8 | 3.22±0.81 |  | 1.52±0.48 | 1.42±0.41 |
| Melistas L [R60] | 171 | 163 |  | 1.07±0.54 | 1.08±0.56 |  | 5.69±1.16 | 5.64±1.1 |  | 3.85±0.95 | 3.81±0.9 |  | 1.35±0.29 | 1.33±0.27 |
| Panagopoulou P [R61] | 17 | 24 |  | 1.1±0.47 | 1.05±0.84 |  | 4.19±0.44 | 3.8±0.59 |  | 2.72±0.26 | 2.3±0.83 |  | 0.98±0.16 | 1.01±0.26 |
| Verduci E [R62] | 67 | 64 |  | 1.18±0.67 | 1.22±0.6 |  | 4.36±0.78 | 4.38±0.67 |  | 2.51±0.78 | 2.49±0.64 |  | 1.31±0.35 | 1.28±0.28 |
| Wang K [R63] | 110 | 86 |  | 2.87±0.53 | 2.9±0.54 |  | - | - |  | 3.30±0.43 | 3.24±0.47 |  | 1.15±0.25 | 1.23±0.28 |
| Wang K [R63] | 76 | 89 |  | 2.04±0.47 | 1.88±0.53 |  | - | - |  | 2.89±0.32 | 2.87±0.33 |  | 1.22±0.25 | 1.32±0.29 |
| Cao LF [R64] | 334 | 340 |  | 0.9±0.7 | 0.99±0.57 |  | 3.9±0.8 | 3.93±0.8 |  | - | - |  | - | - |
| Sone Y [R71] | 67 | 81 |  | 2.12±1.55 | 1.69±0.89 |  | 5.68±0.9 | 5.44±1.02 |  | 3.34±0.86 | 3.26±0.93 |  | 1.38±0.32 | 1.4±0.3 |
| Ronconi V [R72] | 49 | 40 |  | 1.43±0.85 | 1.32±0.95 |  | - | - |  | - | - |  | 1.08±0.28 | 1.35±0.49 |
| Ronconi V [R72] | 47 | 88 |  | 1.48±0.55 | 1.37±1.01 |  | - | - |  | - | -- |  | 1.24±0.31 | 1.2±0.39 |
| Li YP [R74] | 65 | 68 |  | 2.4±1.5 | 2.58±1.96 |  | 4.6±1.1 | 5.0±1.29 |  | 2.7±0.7 | 2.38±0.85 |  | 1.1±0.3 | 1.18±0.37 |
| Li YP [R74] | 25 | 32 |  | 1.29±0.7 | 1.1±0.6 |  | 4.2±0.8 | 4.2±0.6 |  | 2.3±0.6 | 2.3±0.5 |  | 1.28±0.3 | 1.4±0.3 |
| Huang MC [R75] | 344 | 206 |  | 1.54±1.52 | 1.4±1.05 |  | 4.71±0.97 | 4.7±0.93 |  | - | - |  | - | - |
| Leu HB [R83] | 523 | 439 |  | 1.83±1.5 | 1.83±1.64 |  | - | - |  | - | - |  | 1.26±0.37 | 1.27±0.35 |
| Lee JY [R96] | 517 | 508 |  | 1.18±0.55 | 1.26±0.65 |  | 4.67±0.8 | 4.73±0.87 |  | - | - |  | 1.19±0.31 | 1.16±0.33 |
| Cao J [R98] | 34 | 44 |  | 2.54±4.39 | 1.84±1.24 |  | 4.28±0.94 | 3.88±0.73 |  | 3.17±0.79 | 2.82±0.69 |  | 0.98±0.25 | 1.04±0.21 |
| Al-Daghri NM [R102] | 122 | 175 |  | 1.53±0.08 | 1.35± 0.12 |  | 5.1±1.02 | 4.98±0.95 |  | 3.7± 0.9 | 3.58± 0.94 |  | 0.68±0.03 | 0.68±0.04 |
| Riestra P [R103] | 202 | 186 |  | 0.88±0.37 | 0.92±0.43 |  | 4.27±0.74 | 4.07±0.7 |  | 2.54±0.67 | 2.39±0.61 |  | 1.33±0.39 | 1.26±0.35 |
| Riestra P [R103] | 203 | 224 |  | 0.85±0.31 | 0.88±0.32 |  | 4.35±0.69 | 4.45±0.77 |  | 2.55±0.64 | 2.65±0.72 |  | 1.4±0.35 | 1.4±0.37 |
| Kacso IM [R104] | 43 | 60 |  | 2.62±2.03 | 2.24±1.78 |  | - | - |  | 2.71±1.21 | 2.65±1.02 |  | 1.07±0.34 | 1.14±0.34 |
| Kacso IM [R105] | 44 | 61 |  | 2.59±2.02 | 2.18±1.53 |  | - | - |  | 5.01±3.34 | 4.74±3.27 |  | 1.07±0.34 | 1.13±0.34 |
| Kacso IM [R105] | 28 | 20 |  | 1.54±3.18 | 1.56±2.31 |  | - | - |  | 2.7±2.63 | 3.08±1.51 |  | 1.54±1.24 | 1.3±0.3 |
| Elshamaa MF [R106] | 56 | 22 |  | 1.74±0.81 | 1.52±0.48 |  | 4.82±1.22 | 4.61±1.55 |  | - | - |  | 0.64±0.29 | 0.73±0.36 |
| Tsuzaki K [R107] | 135 | 140 |  | 1.15±0.65 | 1.11± 0.5 |  | 4.84±0.93 | 4.82±0.91 |  | - | - |  | 1.37±0.34 | 1.43±0.4 |
| Ohara M [R108] | 196 | 146 |  | 1.65±1.36 | 1.66±1.32 |  | 4.96±1.02 | 4.99±1.07 |  | 3±0.89 | 2.91±0.94 |  | 1.38±0.44 | 1.41±0.47 |
| Zheng HF [R109] | 263 | 249 |  | 1.89±1.40 | 1.69±1.3 |  | 4.92±1.13 | 4.78±1.12 |  | 2.68±0.94 | 2.55± 0.94 |  | 1.4±0.4 | 1.38±0.31 |
| Jochmanová I [R115] | 21 | 26 |  | 1.74±1.23 | 1.38±0.61 |  | 4.81±1.04 | 4.58±0.83 |  | - | - |  | - | - |
| Jochmanová I [R115] | 45 | 45 |  | 1.23±0.51 | 1.09±0.42 |  | 4.93±1.12 | 4.86±1.06 |  | - | - |  | - | - |
| Lee KY [R117] | 46 | 44 |  | - | - |  | - | - |  | - | - |  | 1.25±0.29 | 1.37±0.31 |
| Choe EY [R118] | 351 | 357 |  | 1.8± 1.2 | 1.62± 1.0 |  | 4.7±0.9 | 4.58±0.8 |  | 2.5± 0.8 | 2.58± 0.72 |  | 2.5±0.3 | 2.58±0.72 |
| Galcheva SV [R119] | 84 | 84 |  | - | - |  | 4.3±0.8 | 4.24±0.7 |  | 2.66±0.64 | 2.53±0.58 |  | 1.26±0.32 | 1.29±0.28 |
| Xu J [R120] | 489 | 418 |  | 1.49±0.87 | 1.43±0.68 |  | - | - |  | - | - |  | 1.25±0.29 | 1.27±0.32 |
| Arnaiz-Villena A [R121] | 242 | 79 |  | 2.62±1.88 | 2.61±1.62 |  | 5.57±1.49 | 5.61±1.29 |  | - | - |  | 1.2±0.36 | 1.23±0.35 |
| Gong QL [R123] | 356 | 304 |  | 1.8±1.1 | 1.66±1.26 |  | 3.31±1.05 | 5.22±0.94 |  | 2.75±1.29 | 2.76±1.48 |  | 1.77±0.33 | 1.83±0.36 |
| Kawai T [R124] | 171 | 182 |  | 1.69±0.87 | 1.62±0.91 |  | 5.26±0.98 | 5.28±0.87 |  | 3.01±0.95 | 3.11±0.8 |  | 1.51±0.44 | 1.43±0.42 |
| Hwang JY [R125] | 96 | 123 |  | 1.4±0.12 | 1.43±0.13 |  | 5.08±1.28 | 4.81±1.3 |  | 3.04±1.18 | 2.75±1.2 |  | 1.3±0.45 | 1.31±0.46 |
| Hwang JY [R125] | 133 | 145 |  | 1.44±0.14 | 1.61±0.14 |  | 4.84±1.36 | 4.86±1.4 |  | 2.82±1.27 | 2.81±1.31 |  | 1.26±0.44 | 1.21±0.46 |
| Hwang JY [R125] | 70 | 106 |  | 1.44±0.1 | 1.49±0.12 |  | 4.63±1.23 | 4.79±1.23 |  | 2.83±1.11 | 2.8±1.1 |  | 1.09±0.42 | 1.22±0.42 |
| Wang CH [R126] | 101 | 116 |  | 3.18±2.77 | 3.51 ± 2.25 |  | 5.43±1.63 | 5.15±1.4 |  | 2.68±0.92 | 2.41±0.61 |  | 1.27±0.38 | 1.27±0.34 |
| Sun ZL [R135] | 26 | 24 |  | 2.81±1.47 | 2.46±1.3 |  | 4.93±1.31 | 4.89±1.01 |  | - | - |  | 1.51±0.47 | 1.43±0.44 |
| Sun ZL [R135] | 20 | 20 |  | 1.89±1.07 | 1.66±1.07 |  | 4.88±0.66 | 4.25±1.3 |  | - | - |  | 1.64±0.49 | 1.52±0.41 |
| Park JY [R136] | 322 | 363 |  | 0.74±0.4 | 0.75±0.37 |  | 4.37±0.67 | 4.34±0.71 |  | 2.5±0.59 | 2.49±0.64 |  | 1.52±0.29 | 1.5±0.31 |
| Yang H [R140] | 36 | 44 |  | 1.89±0.86 | 1.92±1.02 |  | 4.57±0.86 | 4.86±0.86 |  | - | - |  | 1.24±0.30 | 1.32±0.29 |
| Tureck LV [R155] | 33 | 41 |  | 1.67±0.81 | 1.76±0.95 |  | 5.33±0.8 | 5.48±0.97 |  | 3.4±0.73 | 3.58±0.87 |  | 1.16±0.27 | 1.12±0.19 |
| Tureck LV [R155] | 69 | 67 |  | 1.44±0.69 | 1.28±0.66 |  | 5.43±0.94 | 5.2±1.05 |  | 3.39±0.85 | 3.28±0.79 |  | 1.38±0.36 | 1.38±0.35 |
| Kato H [R165] | 43 | 35 |  | 1.63±0.56 | 1.75±0.77 |  | 3.98±0.95 | 4.04±0.88 |  | - | - |  | - | - |
| de Luis DA [R166] | 526 | 481 |  | 1.44±0.71 | 1.42±0.56 |  | 5.3±0.85 | 5.33±0.75 |  | 3.29±0.57 | 3.32±0.74 |  | 1.37±0.27 | 1.39±0.23 |
| Momin AA [R172] | 109 | 41 |  | 1.84±0.26 | 1.87±0.3 |  | 5.3±0.81 | 5.28±0.81 |  | 3.31±0.76 | 3.33±0.81 |  | 1.15±0.15 | 1.09±0.13 |
| Zayani N [R174] | 357 | 764 |  | - | - |  | 4.94±1.00 | 4.87±1 |  | 3.36±0.81 | 3.28±0.81 |  | 1.16±0.34 | 1.15±0.33 |
| de Luis DA [R175] | 33 | 31 |  | 1.68±0.31 | 1.3±0.32 |  | 5.24±0.44 | 3.92±0.76 |  | 3.13±0.34 | 2.56±0.75 |  | 1.34±0.22 | 1.32±0.27 |
| Mohseni F [R176] | 33 | 42 |  | - | - |  | 4.97±0.74 | 4.74±1.02 |  | 2.93±0.74 | 2.75±0.95 |  | 1.12±0.22 | 1.14±0.28 |
| Mohseni F [R176] | 39 | 37 |  | - | - |  | 4.45±0.73 | 4.95±0.74 |  | 2.47±0.69 | 2.95±0.7 |  | 1.15±0.29 | 1.25±0.3 |
| Czeczuga-Semeniuk E [R179] | 156 | 136 |  | 1.04±0.66 | 1.01±0.65 |  | 4.54±0.8 | 4.28±0.73 |  | 2.66±0.7 | 2.43±0.61 |  | 1.42±0.42 | 1.44±0.47 |
| Kaur H [R184] | 325 | 225 |  | 1.31±0.46 | 1.61±0.5 |  | 4.27±0.65 | 4.82±0.73 |  | 2.74±0.98 | 3.23±1.02 |  | 0.98±0.24 | 1.03±0.27 |
| Leońska-Duniec A [R185] | 93 | 108 |  | 0.9±0.36 | 0.91±0.37 |  | 4.45±0.69 | 4.38±0.64 |  | 2.31±0.59 | 2.28±0.52 |  | 1.74±0.34 | 1.65±0.35 |
| de Luis DA [R186] | 66 | 69 |  | 1.55±0.32 | 1.35±0.24 |  | 5.26±0.38 | 5.19±0.61 |  | 3.21±0.58 | 3.14±0.8 |  | 1.39±0.21 | 1.28±0.16 |
| de Luis DA [R186] | 35 | 114 |  | 1.35±0.11 | 1.22±0.26 |  | 5.19±0.16 | 5.23±0.26 |  | 3.25±0.34 | 3.17±0.55 |  | 1.29±0.16 | 1.47±0.24 |
| Zheng WW [R193] | 407 | 406 |  | - | - |  | 4.98±0.96 | 4.86±0.95 |  | 2.82±0.79 | 2.73±0.78 |  | 1.22±0.27 | 1.23±0.3 |
| Macías-Gómez NM [R194] | 23 | 41 |  | 2.79±4.63 | 2.29±1.38 |  | 5.35±2.09 | 5.29±1.27 |  | 3.03±1.34 | 3.46±0.98 |  | 1.47±1.06 | 1.24±0.44 |
| Macías-Gómez NM [R194] | 87 | 80 |  | 2.51±2.01 | 2.65±1.75 |  | 5.77±1.29 | 5.8±1.54 |  | 3.34±1.47 | 3.81±1.53 |  | 1.27±0.52 | 1.32±0.49 |
| de Luis DA [R197] | 42 | 37 |  | 1.42±0.69 | 1.41±0.58 |  | 5.46±1.11 | 5.71±0.82 |  | 3.48±0.83 | 3.59±0.49 |  | 1.3±0.27 | 1.4±0.24 |
| de Luis DA [R198] | 95 | 82 |  | 1.43±0.24 | 1.48±0.23 |  | 5.41±0.36 | 5.18±0.6 |  | 3.34±0.52 | 3.26±0.57 |  | 1.39±0.18 | 1.31±0.16 |
| de Luis DA [R198] | 104 | 82 |  | 1.57±0.15 | 1.55±0.12 |  | 5.27±0.21 | 5.25±0.28 |  | 3.25±0.31 | 3.22±0.62 |  | 1.29±0.16 | 1.31±0.24 |
| Aller R [R199] | 65 | 72 |  | 1.36±0.2 | 1.44±0.14 |  | 5.46±0.24 | 5.33±0.57 |  | 3.5±0.26 | 3.27±0.55 |  | 1.35±0.24 | 1.3±0.21 |
| Aller R [R199] | 57 | 75 |  | 1.41±0.26 | 1.4±0.24 |  | 5.31±0.57 | 5.65±0.26 |  | 3.29±0.27 | 3.21±0.31 |  | 1.34±0.26 | 1.42±0.32 |
| Palit SP [R206] | 427 | 514 |  | 1.62±0.88 | 2.09±1.11 |  | 3.96±0.76 | 4.17±0.87 |  | 1.82±0.7 | 2.39±0.93 |  | - | - |
| Garba M [R207] | 118 | 112 |  | 0.99±0.56 | 0.99±0.63 |  | 4.11±0.87 | 4.24±0.96 |  | 2.33±0.60 | 2.36±0.78 |  | 1.42±0.37 | 1.45±0.36 |
| Bains V [R208] | 137 | 179 |  | 1.84±0.56 | 1.88±0.58 |  | 4.49±0.9 | 4.52±0.98 |  | 2.62±0.86 | 2.63±0.96 |  | 1.06±0.26 | 1.08±0.27 |

TG: triglycerides; TC: total cholesterol; LDL-C: low-density lipoprotein cholesterol; HDL-C: high-density lipoprotein cholesterol

**Table S4.** Circulating lipids levels by the genotypes of the rs266729 polymorphism.

| **First author, reference** | **Genotype** | |  | **TG (x±s), mmol/L** | |  | **TC (x±s), mmol/L** | |  | **LDL-C (x±s), mmol/L** | |  | **HDL-C (x±s), mmol/L** | |
| --- | --- | --- | --- | --- | --- | --- | --- | --- | --- | --- | --- | --- | --- | --- |
|  | **CC** | **CG+GG** |  | **CC** | **CG+GG** |  | **CC** | **CG+GG** |  | **CC** | **CG+GG** |  | **CC** | **CG+GG** |
| Stenvinkel P [R5] | 117 | 87 |  | 2.0±1.08 | 2.47±1.73 |  | 5.7±1.08 | 5.51±1.64 |  | - | - |  | 1.2±1.08 | 1.2±0.84 |
| Shin MJ [R22] | 153 | 130 |  | 1.59±0.82 | 1.69±1.01 |  | 4.97±0.87 | 5.02±0.88 |  | 3.1±0.75 | 3.12±0.86 |  | 1.14±0.29 | 1.13±0.29 |
| Petrone A [R25] | 148 | 122 |  | 1.08±0.42 | 1.23±0.52 |  | 4.2±0.72 | 4.42±0.84 |  | 2.55±0.57 | 2.67±0.64 |  | 1.29±0.3 | 1.32±0.27 |
| He L [R28] | 107 | 91 |  | 2.6±1.6 | 2.4±1.49 |  | 5.1±1.1 | 4.9±1.34 |  | - | - |  | - | - |
| He L [R28] | 53 | 48 |  | 1.4±0.6 | 1.8±1.25 |  | 4.6±0.9 | 4.62±0.81 |  | - | - |  | - | - |
| Wang JY [R31] | 120 | 79 |  | 1.96±1.78 | 2.24±1.92 |  | 5.03±1.07 | 5.17±1.22 |  | 2.93±0.8 | 3.08±0.74 |  | 1.42±0.37 | 1.28±0.31 |
| Wang JY [R31] | 88 | 50 |  | 1.56±1.02 | 1.45±0.61 |  | 4.74±0.85 | 4.79±0.91 |  | 2.76±0.64 | 2.89±0.73 |  | 1.46±0.3 | 1.49±0.29 |
| Buzzetti R [R33] | 11 | 8 |  | 1.08±0.47 | 1.3±0.55 |  | 4.95±0.7 | 5.2±0.72 |  | 3.2±0.7 | 2.99±0.6 |  | 1.34±0.3 | 1.36±0.3 |
| Buzzetti R [R33] | 45 | 35 |  | 1.11±0.46 | 1.07±0.51 |  | 4.97±0.66 | 5.1±0.74 |  | 3.15±0.61 | 3.17±0.68 |  | 1.33±0.3 | 1.38±0.27 |
| Hoefle G [R34] | 227 | 175 |  | 2.10±1.49 | 1.94±1.25 |  | 5.57±1.09 | 5.57±1.05 |  | 3.34±0.88 | 3.41±0.85 |  | 1.17±0.31 | 1.14±0.28 |
| Li Z [R40] | 58 | 55 |  | 2.54±2.77 | 2.58±2.59 |  | 5.64±0.99 | 5.93±1.13 |  | 3.38±0.74 | 3.43±0.95 |  | 1.23±0.23 | 1.38±0.38 |
| Sun H [R41] | 119 | 136 |  | 2.7±2.9 | 3.0±3.5 |  | 5.1±1.5 | 4.4±2.0 |  | 3.1±1.0 | 2.9±1.1 |  | 1.3±0.6 | 1.5±1.3 |
| Pérez-Martínez P [R46] | 34 | 25 |  | 0.78±0.33 | 0.69 ±0.28 |  | 4.32±0.61 | 4.01±0.56 |  | 2.69±0.59 | 2.44±0.48 |  | 1.27±0.30 | 1.25±0.24 |
| Jang Y [R47] | 454 | 398 |  | 1.3±0.72 | 1.38±0.81 |  | - | - |  | - | - |  | - | - |
| Ye F [R52] | 161 | 144 |  | 2.1±1.3 | 2.24±1.44 |  | 4.9±1.0 | 4.8±1.18 |  | 3.0±0.8 | 3.3±2.46 |  | 1.2±0.7 | 1.02±0.23 |
| Tsuzaki K [R59] | 16 | 16 |  | 0.94±0.38 | 1.22±0.96 |  | 5.61±1.03 | 5.74±0.91 |  | 3.18±0.75 | 3.15±0.78 |  | 1.68±0.41 | 1.68±0.39 |
| Warodomwichit D [R65] | 604 | 468 |  | - | - |  | 5±1.91 | 4.97±1.68 |  | 3.13±1.53 | 3.13±1.34 |  | 1.17±0.51 | 1.15±0.45 |
| Oguri M [R66] | 1072 | 815 |  | 2.21±1.56 | 2.16±1.19 |  | 5.23±0.97 | 5.29±0.97 |  | - | - |  | 1.2±0.32 | 1.19±0.33 |
| Prior SL [R67] | 427 | 340 |  | 1.7±1.1 | 1.59±0.88 |  | - | - |  | 2.9±0.9 | 2.79±0.91 |  | - | - |
| Cai QY [R68] | 278 | 221 |  | 1.48 ±0.56 | 1.5±0.59 |  | 4.76±1.1 | 4.87±1.23 |  | 2.86±1 | 3.13±1.04 |  | 1.39±0.29 | 1.38±0.3 |
| Sheng TX [R69] | 31 | 24 |  | 2.01±1.54 | 2.44±2.23 |  | 4.97±0.74 | 4.87±0.98 |  | - | - |  | - | - |
| Sheng TX [R69] | 22 | 20 |  | 2.99±3.1 | 2.71±2.23 |  | 5.46±1.33 | 5.19±1.11 |  | - | - |  | - | - |
| Li YP [R74] | 72 | 61 |  | 2.4±1.6 | 2.58±1.85 |  | 4.8±1.1 | 4.83±1.31 |  | 2.4±0.8 | 2.42±0.77 |  | 1.2±0.4 | 1.13±0.21 |
| Li YP [R74] | 26 | 31 |  | 1.0±0.5 | 1.36±0.76 |  | 4.0±0.7 | 4.36±0.62 |  | 2.1±0.5 | 2.44±0.46 |  | 1.4±0.3 | 1.3±0.22 |
| Ferguson JF [R76] | 41 | 407 |  | 2.2±1.28 | 1.74±1.46 |  | 5.6±0.64 | 5.3±1.45 |  | 3.5±0.64 | 3.26±1.46 |  | 1.2±0.64 | 1.1±0.0 |
| Prior SL [R86] | 204 | 179 |  | - | - |  | 5.9±1.1 | 5.83±1.13 |  | 3.3±0.4 | 3.23±0.42 |  | 1.8±0.6 | 1.8±0.61 |
| Wang DL [R87] | 148 | 118 |  | 1.72±0.23 | 1.86±0.28 |  | 4.8±1.06 | 4.91±1.08 |  | 2.57±0.9 | 2.68±0.93 |  | 1.4 ± 0.29 | 1.38±0.31 |
| Min XH [R88] | 94 | 162 |  | 2.02±1.44 | 3.87±1.02 |  | 3.14±0.98 | 5.39±1.26 |  | 2.09±1.01 | 3.86±0.71 |  | 1.26±0.30 | 1.37±0.25 |
| Min Y [R89] | 61 | 66 |  | 1.75±1.23 | 1.76±1.18 |  | 4.62±1.19 | 4.56±1.03 |  | - | - |  | 1.25±0.41 | 1.13±0.33 |
| Min Y [R89] | 53 | 52 |  | 1.84±1.41 | 1.57±1.12 |  | 4.70±1.27 | 4.57±0.97 |  | - | - |  | 1.24±0.44 | 1.18±0.32 |
| Xu J [R110] | 56 | 35 |  | 2.29±0.27 | 1.97±0.18 |  | 5.25±0.15 | 5.45±0.18 |  | 3.2±0.12 | 3.42±0.17 |  | 1.21±0.03 | 1.22±0.05 |
| Mente A [R127] | 621 | 536 |  | 1.59±1.5 | 1.54±1.08 |  | - | - |  | 3.18±0.75 | 3.21±0.67 |  | 1.08±0.25 | 1.06±0.42 |
| Kang Z [R128] | 95 | 58 |  | 2.8±1.3 | 2.33±1.44 |  | 5.2±1.5 | 4.92±1.28 |  | 3.9±0.9 | 3.43±0.78 |  | 1.1±0.4 | 1.03±0.29 |
| Kang Z [R128] | 70 | 56 |  | 2.2±1.5 | 1.98±0.88 |  | 4.8±1.4 | 4.43±0.97 |  | 2.9±0.7 | 3.09±0.84 |  | 1.3±0.5 | 1.08±0.22 |
| Zandoná MR [R129] | 179 | 135 |  | 0.63±0.21 | 0.71±0.36 |  | 3.26±0.67 | 3.4±0.66 |  | 1.79±0.6 | 1.92±0.6 |  | 1.16±0.28 | 1.15±0.26 |
| Ye Y [R130] | 91 | 59 |  | 2.33±1.48 | 2.3±1.4 |  | 5.34±0.95 | 5.47±1.05 |  | 3.48±1.12 | 3.4±1.35 |  | 1.22±0.39 | 1.18±0.28 |
| Ye Y [R130] | 83 | 47 |  | 1.34±1.25 | 1.43±1.01 |  | 5.11±0.91 | 5.27±0.76 |  | 3.08 ± 0.82 | 3.28±0.67 |  | 1.48±0.38 | 1.42±0.37 |
| Wang LJ [R131] | 87 | 63 |  | 2.49±1.66 | 2.61±1.44 |  | 5.64±0.92 | 5.63±0.93 |  | 3.65±0.84 | 3.65±0.85 |  | 1.18±0.34 | 1.1±0.24 |
| Wang LJ [R131] | 94 | 56 |  | 1.09±0.53 | 1.12±0.57 |  | 4.91±0.79 | 4.94±0.85 |  | 3.02±0.73 | 3.07±0.78 |  | 1.49±0.37 | 1.48±0.34 |
| Park JY [R136] | 407 | 280 |  | 0.74±0.39 | 0.76±0.38 |  | 4.31±0.7 | 4.41±0.68 |  | 2.46±0.62 | 2.54±0.61 |  | 1.5±0.31 | 1.52±0.29 |
| Li JQ [R144] | 64 | 62 |  | 1.92±1.03 | 1.86±1.17 |  | 5.88±1.01 | 5.97±0.99 |  | 3.21±1.17 | 3.01±1.03 |  | 1.32±0.24 | 1.36±0.65 |
| Chang JL [R145] | 129 | 97 |  | 2.0±1.2 | 2.14±1.33 |  | 4.8±1.1 | 4.7±1.28 |  | 3.1±0.9 | 3.2±2.36 |  | 1.1±0.6 | 1.16±0.29 |
| Yu J [R146] | 51 | 29 |  | 2.28±1.63 | 3.12± 2.52 |  | 5.57±0.95 | 5.7±0.91 |  | 3.53±0.88 | 3.43±0.9 |  | 1.18±0.28 | 1.12±0.28 |
| Cheng YT [R153] | 322 | 296 |  | 1.43±1.52 | 1.87±1.79 |  | 4.54±0.9 | 4.88±0.98 |  | 2.95±0.74 | 3.36±1.02 |  | 1.4±0.48 | 1.34±0.32 |
| Sun Y [R156] | 465 | 384 |  | 2.96±2.71 | 2.58±2.33 |  | - | - |  | - | - |  | 2.96±2.71 | 2.58±2.33 |
| Kaftan AN [R157] | 73 | 62 |  | 2.81±0.86 | 2.58±0.88 |  | 6.5±1.64 | 5.88±1.95 |  | 4.01±1.67 | 4.13±1.44 |  | 1.53±0.34 | 1.16±0.33 |
| Du SX [R164] | 127 | 119 |  | 2.21±0.96 | 2.85±1.08 |  | 5.21±0.99 | 5.62±1.34 |  | 2.96±0.89 | 3.43±0.94 |  | 1.34±0.42 | 1.37±0.41 |
| Du SX [R164] | 151 | 96 |  | 2.01±0.75 | 2.23±0.81 |  | 5.05±0.89 | 5.04±0.91 |  | 2.61±0.68 | 2.63±0.67 |  | 1.36±0.41 | 1.34±0.32 |
| Du SX [R164] | 219 | 85 |  | 1.41±0.52 | 1.21±0.41 |  | 4.02±0.71 | 4.32±0.81 |  | 2.61±0.71 | 2.59±0.76 |  | 1.55±0.41 | 1.54±0.46 |
| Hsiao TJ [R167] | 592 | 455 |  | 1.68±1.45 | 1.62±1.19 |  | 5.05±0.99 | 5.08±1.02 |  | - | - |  | - | - |
| Wang XX [R168] | 213 | 182 |  | 3.32±1.34 | 3.36±1.57 |  | 5.71±1.67 | 5.84±1.35 |  | 3.12±1.0 | 3.07±1.05 |  | 2.11±0.55 | 2.09±0.61 |
| Yang GZ [R169] | 26 | 94 |  | 3.62±0.74 | 3.73±0.84 |  | 4.45±0.68 | 4.67±0.68 |  | 3.62±0.49 | 3.94±0.59 |  | 1.81±0.36 | 1.84±0.46 |
| Yang XN [R177] | 238 | 130 |  | 1.51±0.77 | 1.71±1.00 |  | 5.09±1.08 | 5.18±1.25 |  | 3.24±0.97 | 3.27±1.19 |  | 1.16±0.36 | 1.06±0.31 |
| Leońska-Duniec A [R185] | 105 | 96 |  | 0.85±0.33 | 0.96±0.39 |  | 4.36±0.61 | 4.46±0.71 |  | 2.22±0.51 | 2.38±0.58 |  | 1.71±0.3 | 1.66±0.39 |
| de Luis DA [R187] | 84 | 65 |  | 1.68±0.31 | 1.69±0.35 |  | 5.25±0.44 | 5.16±0.83 |  | 3.13 ± 0.34 | 3.13±0.61 |  | 1.34±0.21 | 1.37±0.28 |
| Qian XS [R188] | 26 | 50 |  | 2.38±0.48 | 2.91±0.62 |  | 5.51±1.08 | 5.9±1.55 |  | 2.82±0.62 | 3.52±0.94 |  | 1.02±0.34 | 0.82±0.44 |
| Karimi H [R189] | 94 | 61 |  | - | - |  | 4.58±1.18 | 4.68±1.25 |  | 3.01±1.05 | 2.66±0.79 |  | 1.41±0.84 | 1.31±0.86 |
| Cui M [R190] | 95 | 16 |  | 2.17± 5.38 | 1.83±1.54 |  | 4.88±1.00 | 4.96±0.93 |  | 2.85±0.83 | 2.87±0.83 |  | 1.33±0.42 | 1.37±0.54 |
| Nomani H [R200] | 53 | 36 |  | 1.28±0.66 | 1.24±0.47 |  | 4.4±0.69 | 4.16±0.85 |  | 2.22±0.4 | 2.21±0.48 |  | 1.26±0.21 | 1.24±0.25 |
| Nomani H [R200] | 54 | 12 |  | 1.46±0.62 | 1.48±0.56 |  | 4.5±0.8 | 4.89±0.81 |  | 2.85±0.37 | 2.79±0.35 |  | 1.11±0.16 | 1.16±0.29 |
| Divella R [R201] | 47 | 118 |  | 1.37±0.15 | 1.49±0.85 |  | - | - |  | - | - |  | 1.14±0.45 | 0.94±0.41 |
| de Luis DA [R202] | 48 | 35 |  | 1.32±1.42 | 1.32±0.88 |  | 5.25±1.56 | 5.17±1.27 |  | 3.44±1.67 | 3.37±1.24 |  | 1.3±0.73 | 1.32±0.76 |
| Palit SP [R206] | 292 | 279 |  | 1.39±0.89 | 1.71±1.13 |  | 4.18±1.02 | 4.19±1.01 |  | 2.43±0.97 | 2.64±1 |  | - | - |
| de Luis DA [R209] | 81 | 53 |  | 1.49±0.24 | 1.41±0.32 |  | 5.15±0.34 | 5.26±0.31 |  | 3.07±0.21 | 3.29±0.47 |  | 1.36±0.16 | 1.3±0.13 |
| de Luis DA [R209] | 88 | 61 |  | 1.56±0.24 | 1.57±0.13 |  | 5.29±2.09 | 5.07±0.31 |  | 3.13±0.47 | 3.12±0.57 |  | 1.48±1.09 | 1.26±0.23 |

TG: triglycerides; TC: total cholesterol; LDL-C: low-density lipoprotein cholesterol; HDL-C: high-density lipoprotein cholesterol

**Table S5.** Circulating adiponectin levels by the genotypes of the rs2241766 polymorphism.

| **First author, reference** | **Genotype** | | **Adiponectin (x±s), ug/ml** | |
| --- | --- | --- | --- | --- |
|  | **TT** | **TG+GG** | **TT** | **TG+GG** |
| Du PF [R6] | 95 | 71 | 20.4±1.1 | 18.97±1.51 |
| Schäffler A [R8] | 482 | 103 | 9.5±6.59 | 9.4±6.09 |
| Fumeron F [R9] | 219 | 80 | 21.5±9.4 | 23.12±9.56 |
| Fumeron F [R9] | 121 | 37 | 32.8±11.8 | 36.37±15.96 |
| González-Sánchez JL [R10] | 472 | 275 | 10.4±5.1 | 11.0±5.4 |
| Xita N [R11] | 77 | 23 | 11.5±5.5 | 10.8±3.6 |
| Berthier MT [R12] | 117 | 26 | 9.46±4.50 | 11.76±5.96 |
| Jang Y [R14] | 443 | 459 | 6.40±3.79 | 6.49±4.18 |
| Lee YY [R15] | 252 | 241 | 4.32±2.81 | 4.92±3.25 |
| Lee YY [R15] | 201 | 226 | 6.11±3.10 | 8.26±4.27 |
| Kang ES [R16] | 86 | 80 | 4.92±4.12 | 4.84±4.85 |
| Vasseur F [R19] | 516 | 187 | 5.88±2.73 | 5.84±2.79 |
| Pollin TI [R20] | 463 | 85 | 11.7±21.52 | 13.28±5.37 |
| Tankó LB [R21] | 237 | 44 | 4.23±1.58 | 4.02±1.43 |
| Shin MJ [R22] | 161 | 133 | 5.49±3.55 | 5.69±3.81 |
| Mousavinasab F [R23] | 218 | 26 | 10.54±3.8 | 10.49±2.7 |
| Petrone A [R25] | 208 | 62 | 19.95±8.58 | 16.4±7.11 |
| Woo JG [R29] | 505 | 89 | 10.4±4.49 | 9.82±3.59 |
| Woo JG [R29] | 486 | 48 | 8.9±4.41 | 8.08±3.4 |
| Katsuda Y [R35] | 34 | 30 | 5.5±2.3 | 6.8±4.5 |
| Jeng JR [R36] | 116 | 192 | 8.7±10.7 | 8.5±8.57 |
| Jeng JR [R36] | 92 | 168 | 12.5±10.0 | 14.12±11.55 |
| Li LL [R37] | 25 | 11 | 10.6±3 | 8.96±3.48 |
| Li LL [R37] | 24 | 18 | 16.1±7.84 | 15.4±7.21 |
| Wang ZL [R42] | 146 | 118 | 6.52±3.49 | 4.65±2.44 |
| Wang ZL [R42] | 50 | 26 | 12.82±7.81 | 9.36±4.16 |
| Ai ZH [R45] | 89 | 87 | 19.64±6.64 | 17.44±7.47 |
| Jang Y [R47] | 438 | 414 | 7.25±3.77 | 7.52±3.98 |
| Musso G [R49] | 58 | 12 | 5.23±3.91 | 6.47±2.39 |
| Musso G [R49] | 41 | 29 | 10.91±6.29 | 12.84±5.96 |
| Kim SH [R50] | 346 | 362 | 9.83±8.50 | 10.26±9.26 |
| Zietz B [R53] | 386 | 86 | 9.3±5.89 | 8.3±3.71 |
| Mohammadzadeh G [R56] | 26 | 9 | 6.51±2.64 | 6.29±3.40 |
| Mohammadzadeh G [R56] | 20 | 15 | 5.02±2.18 | 4.31±1.73 |
| Tsuzaki K [R59] | 20 | 12 | 6.6±2.7 | 6.6±2.2 |
| Melistas L [R60] | 249 | 93 | 14.31±7.00 | 14.74±7.69 |
| Panagopoulou P [R61] | 33 | 15 | 8.03±4.96 | 10.11±6.19 |
| Chung HK [R70] | 128 | 135 | 4.40±2.6 | 5.08±3.22 |
| Xu L [R73] | 78 | 75 | 6±4.03 | 4.12±2.91 |
| Xu L [R73] | 50 | 23 | 8.88±7.13 | 5.63±4.4 |
| Youpeng B [R77] | 59 | 28 | 4.96±2.87 | 4.39±2.46 |
| Youpeng B [R77] | 15 | 5 | 5.19±0.10 | 3.83±2.93 |
| Youpeng B [R77] | 53 | 28 | 3.75±1.18 | 4.15±1.22 |
| Demirci H [R78] | 70 | 26 | 4.12±1.59 | 4.02±1.76 |
| Demirci H [R78] | 74 | 19 | 6.42±2.76 | 5.98±1.79 |
| Passariello CL [R79] | 157 | 13 | 14.91±6.65 | 16.83±7.91 |
| Zhou Q [R82] | 43 | 47 | 1.58±0.12 | 1.58±0.13 |
| Zhou Q [R82] | 31 | 47 | 1.35±0.11 | 1.4±0.09 |
| Leu HB [R83] | 477 | 485 | 16.09±30.14 | 13.2±8.13 |
| Zhou NN [R84] | 175 | 183 | 6.1±0.75 | 5.51±1.05 |
| Zhou NN [R84] | 42 | 23 | 7.4±0.56 | 6.35±0.7 |
| Wu J [R85] | 47 | 100 | 10.0±2.8 | 7.22±1.62 |
| Al Khaldi RM [R90] | 134 | 66 | 6.8±4 | 6.68±3.88 |
| Park JW [R91] | 533 | 453 | 7.00±3.69 | 7.04±3.69 |
| Wang SJ [R94] | 90 | 90 | 9.79±0.73 | 9.83±0.81 |
| Wang SJ [R94] | 98 | 66 | 12.01±0.72 | 12.13±1.04 |
| Wang SJ [R94] | 31 | 19 | 11.85±1.22 | 13.22±1.91 |
| Lee JY [R96] | 511 | 514 | 5.9±3.6 | 5.5±3.3 |
| Cao J [R98] | 39 | 39 | 3.02±1.23 | 2.53±0.85 |
| Chen XY [R101] | 96 | 152 | 6.83±2.39 | 6.13±2.41 |
| Chen XY [R101] | 125 | 98 | 12.1±2.52 | 10.98±2.46 |
| Oliveira CS [R111] | 394 | 149 | 11.4±7.94 | 12.15±8.26 |
| Mackawy AM [R116] | 40 | 14 | 17.67±2.58 | 16.07±1.72 |
| Mackawy AM [R116] | 35 | 11 | 23.31±3.14 | 22.01±2.5 |
| Lee KY [R117] | 36 | 54 | 8.69±3.54 | 9.39±4.54 |
| Choe EY [R118] | 346 | 362 | 9.8±8.5 | 10.25± 9.25 |
| Galcheva SV [R119] | 129 | 38 | 10.5±5.1 | 11.6±4.7 |
| Gong QL [R123] | 358 | 302 | 14.54±1.44 | 13.03±1.29 |
| Yan CJ [R132] | 123 | 42 | 2.92±0.94 | 2.03±0.86 |
| Yan CJ [R132] | 102 | 24 | 4.26±2.83 | 4.04±3.05 |
| Chen LD [R133] | 102 | 81 | 3.47±0.3 | 2.43±0.23 |
| Yang H [R140] | 63 | 17 | 4.51±3.92 | 6.07±3.64 |
| Zhang C [R141] | 25 | 22 | 8.97±1.53 | 5.29±1.06 |
| Nikolajević-Starčević J [R147] | 338 | 51 | 5.1±1.7 | 5.5±2.3 |
| Su DY [R150] | 49 | 60 | 2.6±0.65 | 1.44±0.76 |
| Cheng YT [R153] | 313 | 305 | 15.79±4.02 | 15.25±3.84 |
| de Oliveira R [R154] | 163 | 86 | 25.7±25.4 | 21.5±24.0 |
| Motawi T [R158] | 42 | 68 | 15.8 ±6.03 | 9.46 ±6.51 |
| Motawi T [R158] | 72 | 18 | 23.8±13.83 | 21.1±6.32 |
| Lanas F [R159] | 72 | 35 | 11.0 ±3.5 | 11.07±3.22 |
| Low CF [R160] | 11 | 15 | 7.42±2.57 | 6.70±2.64 |
| Low CF [R160] | 35 | 18 | 8.91±3.44 | 7.43±1.96 |
| Han Y [R161] | 53 | 75 | 7.64±1.41 | 6.16±1.17 |
| Han Y [R161] | 75 | 65 | 10.63±1.06 | 9.69±0.89 |
| Wu HL [R162] | 126 | 179 | 10.71±2.36 | 8.21±2.03 |
| Wu HL [R162] | 23 | 215 | 8.56±5.59 | 11.95±9.59 |
| Du SX [R164] | 126 | 120 | 14.98±3.92 | 13.38±3.21 |
| Du SX [R164] | 127 | 120 | 15.63±4.01 | 14.26±3.89 |
| Du SX [R164] | 185 | 119 | 19.36±3.02 | 19.01±2.97 |
| Nambiar V [R170] | 213 | 69 | 18.33±2.85 | 16.06±3.9 |
| Al Hannan FA [R171] | 92 | 37 | 6.5±4.1 | 3.4±3.24 |
| Al Hannan FA [R171] | 42 | 6 | 7.8±4.3 | 3.8±1.1 |
| Shi JK [R178] | 60 | 78 | 6.07±1.29 | 4.67±1.51 |
| Shi JK [R178] | 61 | 41 | 10.54±1.10 | 10.49±1.04 |
| Chang CS [R182] | 118 | 116 | 6.80±0.32 | 6.77±0.34 |
| Gumanova NG [R203] | 276 | 39 | 8.86±5.80 | 9.67±6.5 |
| Gumanova NG [R203] | 115 | 17 | 11.38±7.85 | 13.11±6.27 |

**Table S6.** Circulating adiponectin levels by the genotypes of the rs1501299 polymorphism.

| **First author, reference** | **Genotype** | | **Adiponectin (x±s), ug/ml** | |
| --- | --- | --- | --- | --- |
|  | **GG** | **GT+TT** | **GG** | **GT+TT** |
| Du PF [R6] | 15 | 151 | 44.7±1.3 | 23.44±2.88 |
| Yoshioka K [R7] | 170 | 176 | 5.6±3.7 | 6.03±3.67 |
| Fumeron F [R9] | 149 | 146 | 22.2±10.2 | 21.7±8.7 |
| Fumeron F [R9] | 93 | 64 | 33.8±12.8 | 33.37±13.18 |
| Berthier MT [R12] | 60 | 63 | 9.33±4.16 | 10.02±5.09 |
| Jang Y [R14] | 451 | 451 | 6.22±3.61 | 6.64±4.18 |
| Lee YY [R15] | 224 | 269 | 4.66±3.13 | 4.6±2.99 |
| Lee YY [R15] | 225 | 202 | 7.84±3.64 | 6.11±4.59 |
| Kang ES [R16] | 91 | 75 | 5.43±5.17 | 4.24±3.37 |
| Vasseur F [R19] | 337 | 298 | 5.26±2.75 | 5.86±3.25 |
| Pollin TI [R20] | 345 | 199 | 11.3±18.57 | 12.31±6.6 |
| Tankó LB [R21] | 143 | 139 | 4.10±1.58 | 4.37±1.53 |
| Shin MJ [R22] | 145 | 149 | 5.42±3.61 | 5.74±3.91 |
| Mousavinasab F [R23] | 134 | 110 | 9.1±3.3 | 11.22±3.94 |
| Petrone A [R25] | 162 | 108 | 18.6±7.24 | 19.46±8.58 |
| Iacobellis G [R27] | 23 | 39 | 25.6±14.8 | 30.74±11.15 |
| Woo JG [R29] | 335 | 286 | 10.1± 3.66 | 10.77±4.55 |
| Woo JG [R29] | 229 | 315 | 8.6±4.54 | 9.02±4.61 |
| Yu SY [R32] | 98 | 93 | 4.21±4.85 | 3.88±4.85 |
| Yu SY [R32] | 69 | 91 | 6.64±5.9 | 6.93±6.65 |
| Ishibashi K [R38] | 89 | 147 | 6.7±3.77 | 7.2±3.64 |
| Wang ZL [R42] | 113 | 151 | 4.86±2.64 | 4.90±3.20 |
| Wang ZL [R42] | 28 | 48 | 9.13±3.63 | 11.04±7.11 |
| Jang Y [R47] | 416 | 436 | 7.01±3.67 | 7.74±3.93 |
| Musso G [R49] | 17 | 53 | 5.91±3.58 | 5.47±4.02 |
| Musso G [R49] | 38 | 32 | 11.85±4.38 | 10.59±3.79 |
| Kim SH [R50] | 351 | 357 | 9.61±8.33 | 10.49±9.41 |
| Kyriakou T [R54] | 391 | 355 | 19.51±12.7 | 21.43±11.63 |
| Kyriakou T [R54] | 927 | 699 | 7.89±3.77 | 8.69±4.22 |
| Mohammadzadeh G [R56] | 17 | 18 | 6.27±3.23 | 6.62±2.40 |
| Mohammadzadeh G [R56] | 18 | 17 | 4.95±2.05 | 4.47±1.9 |
| Melistas L [R60] | 171 | 163 | 14.07±7.43 | 15.16±6.86 |
| Panagopoulou P [R61] | 17 | 24 | 7.77±5.65 | 9.73±5.19 |
| Verduci E [R62] | 67 | 64 | 18.5±8.3 | 18.4±9.2 |
| Wang K [R63] | 110 | 86 | 8.18±2.64 | 9.09±2.48 |
| Wang K [R63] | 76 | 89 | 11.43±2.12 | 12.13±2.5 |
| Chung HK [R70] | 136 | 127 | 4.95±3.03 | 4.54±2.8 |
| Huang MC [R75] | 344 | 206 | 9.8±8.5 | 8.49±8.63 |
| Youpeng B [R77] | 15 | 72 | 3.11±1.32 | 5.08±2.84 |
| Youpeng B [R77] | 4 | 16 | 3.40±0.89 | 4.00±2.95 |
| Youpeng B [R77] | 19 | 62 | 3.85±1.24 | 3.89±1.20 |
| Passariello CL [R80] | 79 | 91 | 14.69±5.99 | 15.31±7.37 |
| Leu HB [R84] | 523 | 439 | 13.54±9.54 | 13.2±9.7 |
| Al Khaldi RM [R90] | 15 | 185 | 9.8±3 | 6.46±3.29 |
| Park JW [R91] | 501 | 485 | 6.94±3.81 | 7.07±3.78 |
| Lee JY [R96] | 517 | 508 | 5.6±3.3 | 5.8±3.6 |
| Cao J [R98] | 34 | 44 | 2.67±0.76 | 2.84±1.25 |
| Riestra P [R103] | 202 | 186 | 11.2±6.5 | 11.41±7.14 |
| Riestra P [R103] | 203 | 224 | 14.8±8.0 | 15.4±7.96 |
| Kacso IM [R104] | 43 | 60 | 8.71±10.49 | 11.62±12.69 |
| Kacso IM [R105] | 44 | 61 | 8.71±10.61 | 11.45±12.69 |
| Kacso IM [R105] | 28 | 20 | 15.42±19.1 | 19.31±23.5 |
| Tsuzaki K [R107] | 135 | 140 | 8.6±6.1 | 8.43±5.51 |
| Zheng HF [R109] | 263 | 249 | 7.17±3.18 | 7.96±2.53 |
| Oliveira CS [R111] | 246 | 300 | 11.1±7.84 | 11.85±7.78 |
| Saito M [R112] | 258 | 267 | 38.4±22.1 | 38.56±24.11 |
| Gui MH [R113] | 68 | 84 | 7.94±1.62 | 7.68±1.74 |
| Gui MH [R113] | 69 | 86 | 13.59±1.59 | 14.15±1.54 |
| Lee KY [R117] | 46 | 44 | 9.31±4.27 | 8.93±4.1 |
| Choe EY [R118] | 351 | 357 | 9.6±8.3 | 10.46±9.44 |
| Galcheva SV [R119] | 84 | 84 | 11.2±5.0 | 10.24±5 |
| Gong QL [R123] | 356 | 304 | 12.95±1.33 | 14.9±1.09 |
| Hwang JY [R125] | 96 | 123 | 4.95±10.68 | 4.48±11.98 |
| Hwang JY [R125] | 133 | 145 | 3.49±12.57 | 3.6±13 |
| Hwang JY [R125] | 70 | 106 | 4.81±9.45 | 4.22±11.22 |
| Ramya K [R134] | 555 | 274 | 11.3±4.1 | 5.36±1.62 |
| Ramya K [R134] | 669 | 220 | 11.6±4.5 | 5.03±1.33 |
| Ramya K [R134] | 552 | 266 | 11.1±3.9 | 5.07±1.43 |
| Ramya K [R134] | 667 | 228 | 11.8±4.6 | 5.3±1.55 |
| Nikolajević-Starčević J [R147] | 156 | 145 | 4.9±1.8 | 5.32±1.81 |
| Li Y [R148] | 269 | 330 | 6.81±3.91 | 7.08±4.06 |
| Tureck LV [R155] | 21 | 16 | 3.96±2.18 | 5.01±2.13 |
| Tureck LV [R155] | 48 | 43 | 4.52±1.70 | 4.38±2.33 |
| Lanas F [R159] | 6 | 101 | 6.9±4.6 | 11.33±3.12 |
| Klemettilä JP [R163] | 89 | 91 | 3.12±1.40 | 3.37±1.83 |
| de Luis DA [R166] | 526 | 481 | 20.2±2.4 | 15.43±3.24 |
| Nambiar V [R170] | 23 | 259 | 15.64±4.52 | 15.68±3.9 |
| Al Hannan FA [R171] | 119 | 10 | 5.3±3.9 | 8.44±5.33 |
| Al Hannan FA [R171] | 41 | 7 | 7.15±4.9 | 7.96±3.29 |
| de Luis DA [R175] | 33 | 31 | 11.7±5.1 | 12.9±6.0 |
| de Luis DA [R186] | 66 | 69 | 11.7±4.1 | 12.5±7.1 |
| de Luis DA [R186] | 35 | 114 | 12.1±6.4 | 11.5±8.1 |
| Khabour OF [R191] | 92 | 308 | 2.46±0.84 | 2.34±0.97 |
| de Luis DA [R197] | 42 | 37 | 25.7±11.4 | 28.7±13.4 |
| de Luis DA [R198] | 95 | 82 | 9.6±4.0 | 10.5±3.1 |
| de Luis DA [R198] | 104 | 82 | 11.9±6.4 | 11.0±8.9 |
| Aller R [R199] | 65 | 72 | 10.0±3.1 | 10.2±4.1 |
| Aller R [R199] | 57 | 75 | 11.0±4.9 | 9.8±5.4 |
| Geriki S [R204] | 172 | 107 | 14.21±9.34 | 16.2±8.49 |

**Table S7.** Circulating adiponectin levels by the genotypes of the rs266729 polymorphism.

| **First author, reference** | **Genotype** | | **Adiponectin (x±s), ug/ml** | |
| --- | --- | --- | --- | --- |
|  | **CC** | **CG+GG** | **CC** | **CG+GG** |
| Fumeron F [R9] | 180 | 118 | 22.5±9.3 | 21.07±9.57 |
| Fumeron F [R9] | 81 | 76 | 32.2±13.0 | 35.13±12.71 |
| Vasseur F [R19] | 414 | 289 | 6.05±2.65 | 5.39±2.84 |
| Pollin TI [R20] | 170 | 383 | 11.8±13.04 | 12.21±8.04 |
| Tankó LB [R21] | 138 | 149 | 4.55±1.68 | 3.85±1.36 |
| Shin MJ [R22] | 153 | 130 | 5.63±3.96 | 5.49±3.53 |
| Petrone A [R25] | 148 | 122 | 20.55±7.7 | 17.65±7.67 |
| He L [R28] | 107 | 91 | 6.8±3.6 | 6.11±2.97 |
| He L [R28] | 53 | 48 | 8.3±3.8 | 6.44±2.88 |
| Schwarz PE [R30] | 309 | 241 | 10.53±14.59 | 9.59±6.33 |
| Buzzetti R [R33] | 11 | 8 | 30.1±13.8 | 26±11.9 |
| Buzzetti R [R33] | 45 | 35 | 33.5±13.7 | 27.8±10.6 |
| Jang Y [R47] | 454 | 398 | 7.54±4.05 | 7.24±3.64 |
| Yang M [R55] | 100 | 112 | 4.8±3.0 | 4.64±2.88 |
| Yang M [R55] | 154 | 124 | 5.1±3.1 | 5.0±2.84 |
| Yang M [R55] | 325 | 260 | 8.7±4.6 | 8.48±4.11 |
| Tsuzaki K [R59] | 16 | 16 | 6.2±1.7 | 7.0±3.0 |
| Cai QY [R68] | 278 | 221 | 5.7±3.58 | 4.72±2.34 |
| Ferguson JF [R76] | 41 | 407 | 3.4±1.92 | 3.82±2.91 |
| Prior SL [R86] | 204 | 179 | 14.9±4.6 | 15.02±4.95 |
| Park JW [R91] | 534 | 452 | 7.10±3.7 | 6.86±3.68 |
| Dhillon PK [R92] | 483 | 397 | 8.78±0.61 | 8.66±0.61 |
| Saito M [R112] | 322 | 204 | 34.1±20.2 | 45.36±27.5 |
| Roszkowska-Gancarz M [R114] | 13 | 27 | 17.19±9.86 | 11.26±4.2 |
| Ramya K [R134] | 572 | 501 | 8.4±5.0 | 6.69±2.99 |
| Ramya K [R134] | 621 | 432 | 11.1±5.5 | 8.49±3.06 |
| Ramya K [R134] | 521 | 375 | 8.1±4.8 | 7.81±4.06 |
| Ramya K [R134] | 670 | 552 | 8.6±5.3 | 8.69±4.88 |
| Li Y [R148] | 331 | 268 | 6.93±3.87 | 7.13±4.19 |
| Gu CY [R149] | 148 | 157 | 7.31±2.10 | 7.42±2.12 |
| Gu CY [R149] | 159 | 171 | 7.54±2.35 | 7.41±2.26 |
| Cheng YT [R153] | 322 | 296 | 16.82±4.24 | 14.08±3.08 |
| Lanas F [R159] | 35 | 72 | 11.2±3.3 | 10.91±3.4 |
| Du SX [R164] | 127 | 119 | 16.82±4.47 | 13.62±4.32 |
| Du SX [R164] | 151 | 96 | 15.56±4.31 | 13.26±4.21 |
| Du SX [R164] | 219 | 85 | 19.02±3.11 | 18.78±3.21 |
| Yang GZ [R169] | 26 | 94 | 2.42±0.22 | 5.74±2.1 |
| Yang XN [R177] | 238 | 130 | 35.95±21.13 | 33.16±16.63 |
| Shi JK [R178] | 70 | 68 | 5.94±1.38 | 4.44±1.58 |
| Shi JK [R178] | 68 | 34 | 10.97±1.01 | 9.62±1.29 |
| de Luis DA [R187] | 84 | 65 | 20.8±5 | 12.1±5 |
| Nomani H [R200] | 53 | 36 | 23.9±13 | 25±18.2 |
| Nomani H [R200] | 54 | 12 | 18.6±11.2 | 13.2±6.3 |
| de Luis DA [R202] | 48 | 35 | 28.7±5.1 | 18.5±5 |
| Gumanova NG [R203] | 170 | 145 | 9.42±6.23 | 8.41±5.46 |
| Gumanova NG [R203] | 70 | 62 | 11.23±7.10 | 11.95±8.29 |
| Geriki S [R204] | 137 | 142 | 19.15±10.42 | 12.5±5.65 |
| de Luis DA [R209] | 81 | 53 | 9.6±4.0 | 10.1±3.9 |
| de Luis DA [R209] | 88 | 61 | 10.1±4.1 | 11.9±8.1 |

**Table S8.** Effects of rs2241766, rs1501299 and rs266729 on circulating adiponectin levels.

| Groups or subgroups | Comparisons  (Subjects) | *P*_H_ | SMD (95% CI) | *P*_SMD_ |  | Groups or subgroups | Comparisons  (Subjects) | *P*_H_ | SMD (95% CI) | *P*_SMD_ |
| --- | --- | --- | --- | --- | --- | --- | --- | --- | --- | --- |
| ***Overall results*** | | | | |  | ***Recalculated results that eliminated heterogeneity*** | | | | |
| **Adiponectin (rs2241766 )** | | | | |  | **Adiponectin (rs2241766 )** | | | | |
| All | 97 (25 042) | <0.001 | -0.25 (-0.34--0.15) | <0.001 |  | All | 56 (12 555) | 0.13 | -0.10 (-0.14--0.06) | <0.001 |
| ***Ethnicity*** |  |  |  |  |  | ***Ethnicity*** |  |  |  |  |
| Chinese | 43 (8 455) | <0.001 | -0.53 (-0.71--0.34) | <0.001 |  | Chinese | 20 (4 171) | 0.30 | -0.15 (-0.21--0.09) | <0.001 |
| Korean | 12 (6 914) | <0.001 | 0.10 (0.00-0.19) | 0.05 |  | Korean | 4 (1 575) | 0.40 | -0.06 (-0.16-0.04) | 0.25 |
| Caucasian | 22 (6 847) | 0.04 | 0.05 (-0.03-0.13) | 0.21 |  | Caucasian | 17 (5 111) | 0.15 | -0.02 (-0.09-0.05) | 0.62 |
| Latino | 2 (792) | 0.11 | -0.02 (-0.28-0.23) | 0.87 |  | - | - | - | - | - |
| Middle eastern | 11 (936) | 0.01 | -0.42 (-0.67--0.17) | <0.001 |  | Middle eastern | 9 (697) | 0.47 | -0.20 (-0.38--0.03) | 0.02 |
| Other ethnic | 3 (186) | 0.35 | -0.17 (-0.49-0.14) | 0.29 |  | Other ethnic | 3 (186) | 0.35 | -0.17 (-0.47-0.14) | 0.29 |
| ***Gender*** |  |  |  |  |  | ***Gender*** |  |  |  |  |
| Male | 9 (1 932) | <0.001 | -0.29 (-0.64-0.07) | 0.11 |  | Male | 4 (957) | 0.72 | -0.00 (-0.16-0.16) | 0.99 |
| Female | 23 (3 570) | <0.001 | -0.28 (-0.49--0.07) | 0.01 |  | Female | 15 (1 622) | 0.33 | -0.08 (-0.20-0.04) | 0.19 |
| ***Disease status*** |  |  |  |  |  | ***Disease status*** |  |  |  |  |
| CAD | 5 (1 204) | <0.001 | -0.29 (-0.61-0.03) | 0.07 |  | CAD | 2 (447) | 0.78 | 0.16 (-0.12-0.44) | 0.25 |
| T2DM | 15 (3 976) | <0.001 | -0.32 (-0.56--0.08) | 0.01 |  | T2DM | 5 (940) | 0.49 | -0.17 (-0.31--0.02) | 0.02 |
| Obesity | 12 (2 222) | <0.001 | -0.20 (-0.43-0.04) | 0.10 |  | Obesity | 10 (2 011) | 0.04 | -0.10 (-0.20--0.01) | 0.04 |
| PCOS | 3 (478) | 0.02 | -0.34 (-0.80-0.13) | 0.15 |  | PCOS | 2 (196) | 0.82 | -0.10 (-0.42-0.23) | 0.56 |
| Healthy subjects | 37 (10 318) | <0.001 | -0.04 (-0.15-0.06) | 0.42 |  | Healthy subjects | 23 (5 167) | 0.31 | -0.09 (-0.15--0.02) | 0.01 |
| Children subjects | 5 (802) | <0.001 | -0.20 (-0.83-0.43) | 0.54 |  | Children subjects | 4 (655) | 0.10 | -0.07 (-0.26-0.13) | 0.52 |
| Pregnant woman | 4 (261) | 0.01 | -0.65 (-1.24--0.07) | 0.03 |  | Pregnant woman | 3 (133) | 0.42 | -0.32 (-0.68-0.05) | 0.09 |
| **Adiponectin (rs1501299)** | | | | |  | **Adiponectin (rs1501299)** | | | | |
| All | 89 (30 414) | <0.001 | -0.06 (-0.19-0.08) | 0.42 |  | All | 75 (23 369) | 0.35 | 0.09 (0.07-0.12) | <0.001 |
| ***Ethnicity*** |  |  |  |  |  | ***Ethnicity*** |  |  |  |  |
| Chinese | 16 (4 723) | <0.001 | -0.10 (-0.48-0.28) | 0.61 |  | Chinese | 12 (3 260) | 0.05 | 0.11 (0.04-0.18) | <0.01 |
| Japanese | 4 (1 382) | 0.69 | 0.05 (-0.06-0.15) | 0.37 |  | Japanese | 4 (1 382) | 0.69 | 0.05 (-0.06-0.15) | 0.37 |
| Korean | 17 (7 938) | <0.01 | 0.00 (-0.07-0.07) | 0.99 |  | Korean | 15 (7 345) | 0.69 | 0.06 (0.01-0.11) | 0.01 |
| Caucasian | 35 (10 207) | <0.001 | 0.05 (-0.12-0.23) | 0.55 |  | Caucasian | 33 (8 956) | 0.57 | 0.12 (0.08-0.16) | <0.001 |
| Latino | 3 (674) | 0.38 | 0.09 (-0.06-0.25) | 0.22 |  | Latino | 3 (674) | 0.38 | 0.09 (-0.06-0.25) | 0.22 |
| Indian | 5 (3 713) | <0.001 | -1.11 (-1.69--0.53) | <0.001 |  | - | - | - | - | - |
| Middle eastern | 6 (847) | <0.01 | -0.08 (-0.52-0.35) | 0.71 |  | Middle eastern | 5 (647) | 0.11 | -0.02 (-0.21-0.17) | 0.85 |
| ***Gender*** |  |  |  |  |  | ***Gender*** |  |  |  |  |
| Male | 6 (1 278) | <0.01 | 0.16 (-0.07-0.38) | 0.18 |  | Male | 5 (1 034) | 0.53 | 0.02 (-0.10-0.14) | 0.77 |
| Female | 17 (6 084) | 0.40 | 0.14 (0.08-0.19) | <0.001 |  | Female | 16 (5 997) | 0.65 | 0.14 (0.08-0.19) | <0.001 |
| ***Disease status*** |  |  |  |  |  | ***Disease status*** |  |  |  |  |
| T2DM | 17 (5 371) | <0.001 | -0.02 (-0.31-0.27) | 0.90 |  | T2DM | 15 (4 376) | 0.18 | 0.08 (0.02-0.14) | 0.01 |
| Obesity | 19 (4 477) | <0.001 | -0.12 (-0.54-0.30) | 0.58 |  | Obesity | 17 (2 652) | 0.59 | 0.10 (0.02-0.17) | 0.02 |
| Healthy subjects | 36 (14 959) | <0.001 | 0.01 (-0.16-0.18) | 0.92 |  | Healthy subjects | 31 (12 397) | 0.37 | 0.10 (0.07-0.14) | <0.001 |
| Children subjects | 8 (2 120) | 0.77 | 0.03 (-0.05-0.12) | 0.43 |  | Children subjects | 8 (2 120) | 0.77 | 0.03 (-0.05-0.12) | 0.43 |
| **Adiponectin (rs266729)** | | | | |  | **Adiponectin (rs266729)** | | | | |
| All | 49 (18 344) | <0.001 | -0.22 (-0.33--0.12) | <0.001 |  | All | 30 (11 661) | 0.56 | -0.05 (-0.09--0.01) | 0.01 |
| ***Ethnicity*** |  |  |  |  |  | ***Ethnicity*** |  |  |  |  |
| Chinese | 17 (5 250) | <0.001 | -0.23 (-0.43--0.04) | 0.02 |  | Chinese | 9 (3 179) | 0.84 | -0.04 (-0.11-0.03) | 0.24 |
| Japanese | 2 (558) | 0.67 | 0.47 (0.30-0.65) | <0.001 |  | - | - | - | - | - |
| Korean | 3 (2 121) | 0.96 | -0.07 (-0.15-0.02) | 0.13 |  | Korean | 3 (2 121) | 0.96 | -0.07 (-0.15-0.02) | 0.13 |
| Caucasian | 19 (5 630) | <0.001 | -0.28 (-0.45--0.10) | <0.01 |  | Caucasian | 12 (3 949) | 0.09 | -0.07 (-0.13--0.00) | 0.05 |
| Indian | 5 (4 523) | <0.001 | -0.35 (-0.62--0.08) | 0.01 |  | Indian | 2 (2 118) | 0.36 | -0.02 (-0.10-0.07) | 0.70 |
| ***Gender*** |  |  |  |  |  | ***Gender*** |  |  |  |  |
| Male | 6 (2 147) | 0.51 | -0.13 (-0.22--0.05) | <0.01 |  | Male | 6 (2 147) | 0.51 | -0.13 (-0.22--0.05) | <0.01 |
| Female | 8 (1 859) | <0.001 | -0.25 (-0.54-0.03) | 0.08 |  | Female | 5 (1 253) | 0.09 | -0.03 (-0.15-0.08) | 0.54 |
| ***Disease status*** |  |  |  |  |  | ***Disease status*** |  |  |  |  |
| CAD | 3 (693) | <0.001 | -0.28 (-0.73-0.18) | 0.23 |  | CAD | 2 (447) | 0.20 | -0.09 (-0.28-0.09) | 0.33 |
| T2DM | 7 (3 020) | <0.001 | 0.01 (-0.27-0.30) | 0.94 |  | T2DM | 4 (1 328) | 0.84 | -0.11(-0.22--0.00) | 0.05 |
| Obesity | 10 (2 766) | <0.001 | -0.45 (-0.77--0.13) | 0.01 |  | Obesity | 5 (1 412) | 0.35 | -0.07 (-0.17-0.04) | 0.22 |
| Healthy subjects | 18 (7 601) | <0.001 | -0.17 (-0.32--0.02) | 0.03 |  | Healthy subjects | 12 (5 492) | 0.60 | -0.03 (-0.09-0.02) | 0.22 |

SMD: standardized mean difference; 95% CI: 95% confidence interval; *P*_H:_ *P*_Heterogeneity;_ CAD: coronary artery disease; T2DM: type 2 diabetes mellitus; PCOS: polycystic ovarian syndrome; TG: triglycerides; TC: total cholesterol; LDL-C: low-density lipoprotein cholesterol; HDL-C: high-density lipoprotein cholesterol


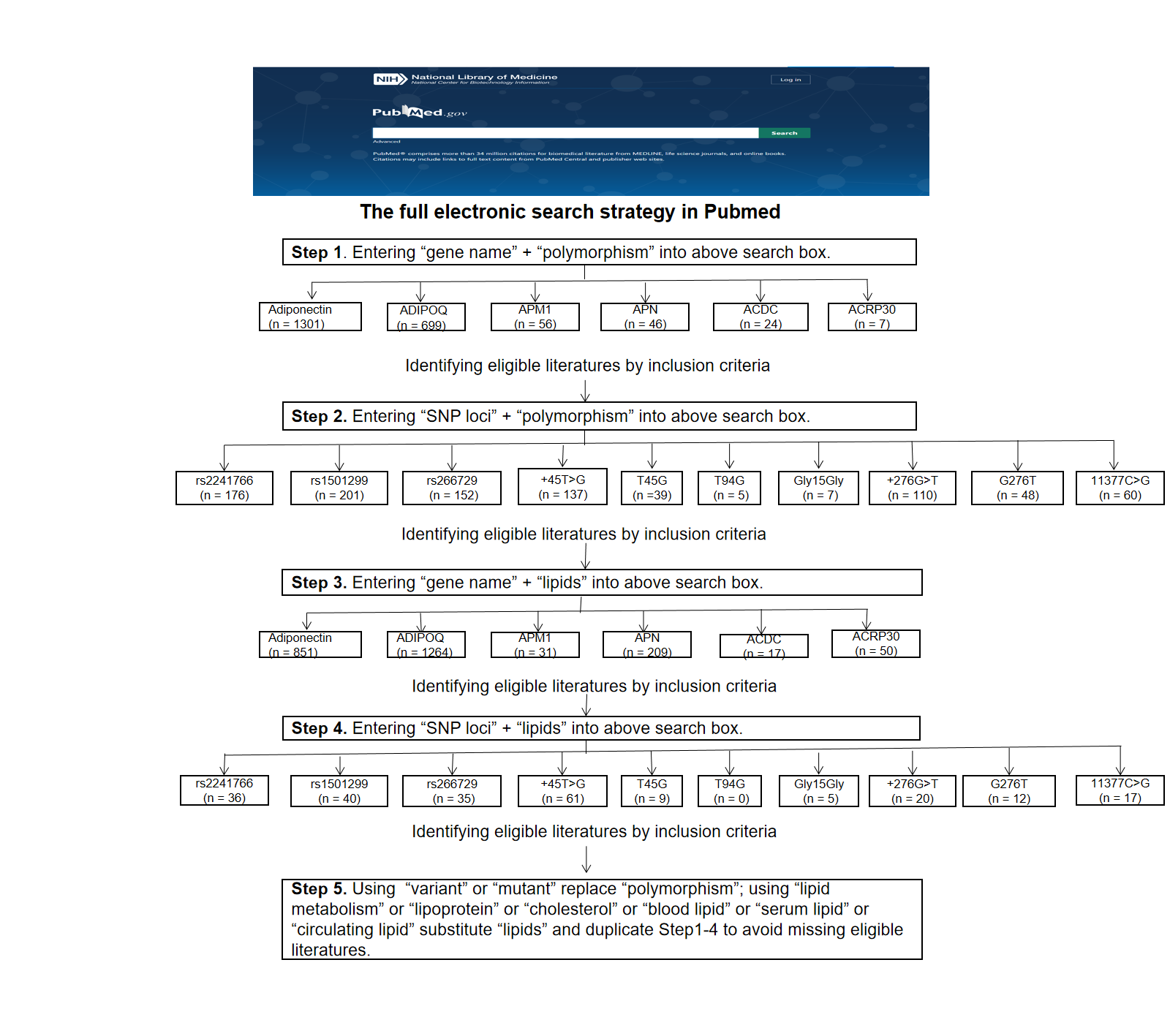


Figure S1. The full electronic search strategy in Pubmed.

**Figure S2.** Forest plot of the meta-analysis between the rs2241766 polymorphism and circulating TG levels.

**Figure S3.** Forest plot of the meta-analysis between the rs2241766 polymorphism and circulating TC levels.

**Figure S4.** Forest plot of the meta-analysis between the rs2241766 polymorphism and circulating HDL-C levels.

**Figure S5.** Forest plot of the meta-analysis between the rs1501299 polymorphism and circulating TG levels.

**Figure S6.** Forest plot of the meta-analysis between the rs1501299 polymorphism and circulating TC levels.

**Figure S7.** Forest plot of the meta-analysis between the rs1501299 polymorphism and circulating HDL-C levels.

**Figure S8.** Forest plot of the meta-analysis between the rs266729 polymorphism and circulating TG levels.

**Figure S9.** Forest plot of the meta-analysis between the rs266729 polymorphism and circulating TC levels.

**Figure S10.** Forest plot of the meta-analysis between the rs266729 polymorphism and circulating HDL-C levels.

**Figure S11.** Forest plot of the meta-analysis between the rs2241766 polymorphism and circulating adiponectin levels.

**Figure S12.** Forest plot of the meta-analysis between the rs266729 polymorphism and circulating adiponectin levels.

**Figure S13.** Forest plot of the meta-analysis between the rs1501299 polymorphism and circulating adiponectin levels.


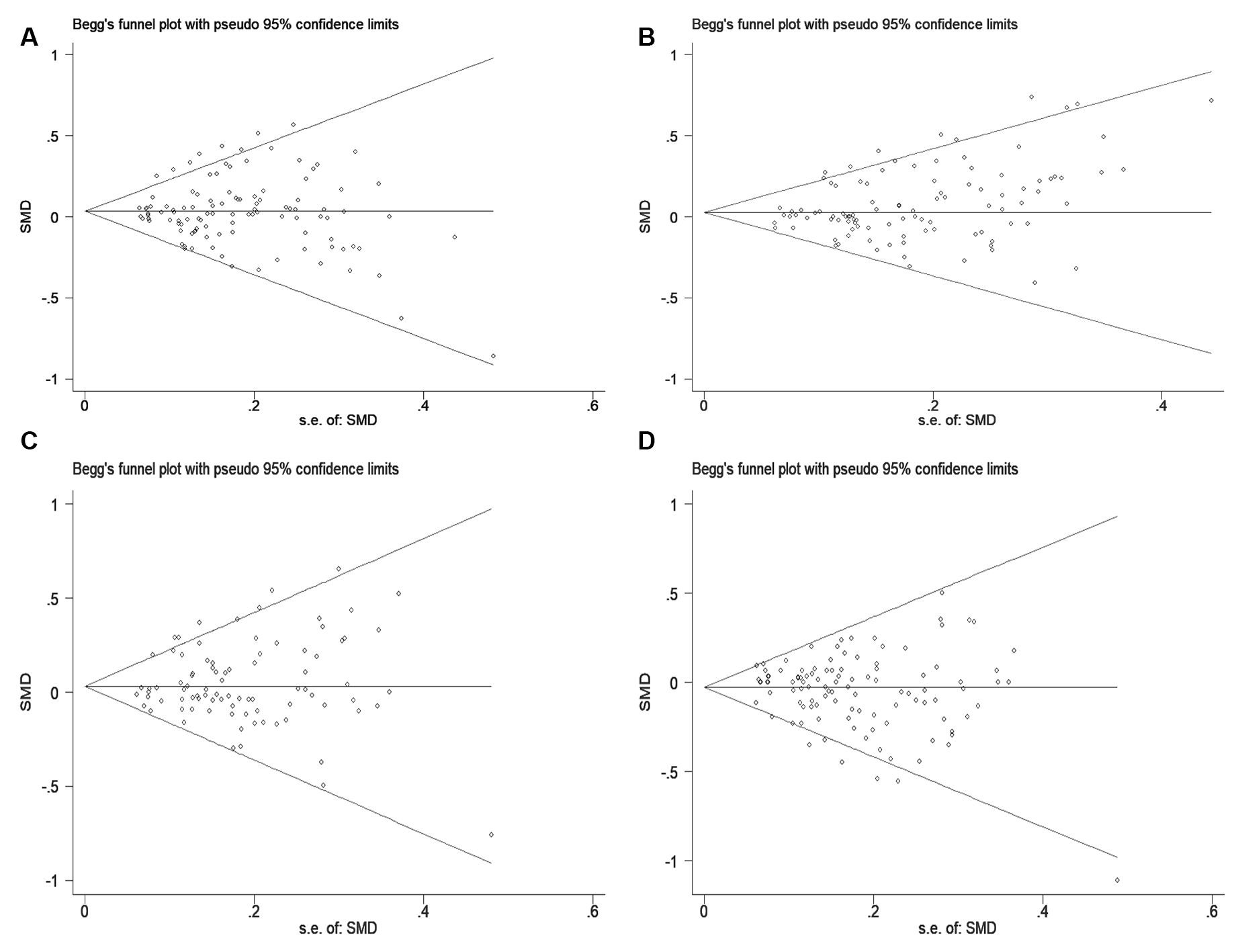


**Figure S14.** Begg’s funnel plot evaluate the publication bias of the effects of the rs2241766 polymorphism on lipid levels. Each small circle represents a separate study. The diverging lines represent 95% CI and the central line is SMD. The funnel plots should be asymmetric when there is publication bias and symmetric in the case of no publication bias [**A**: rs2241766 on TG (*P* = 0.88); **B**: rs2241766 on TC (*P* = 0.06); **C**: rs2241766 on LDL-C (*P* = 0.86); **D**: rs2241766 on HDL-C (*P* = 0.40)].


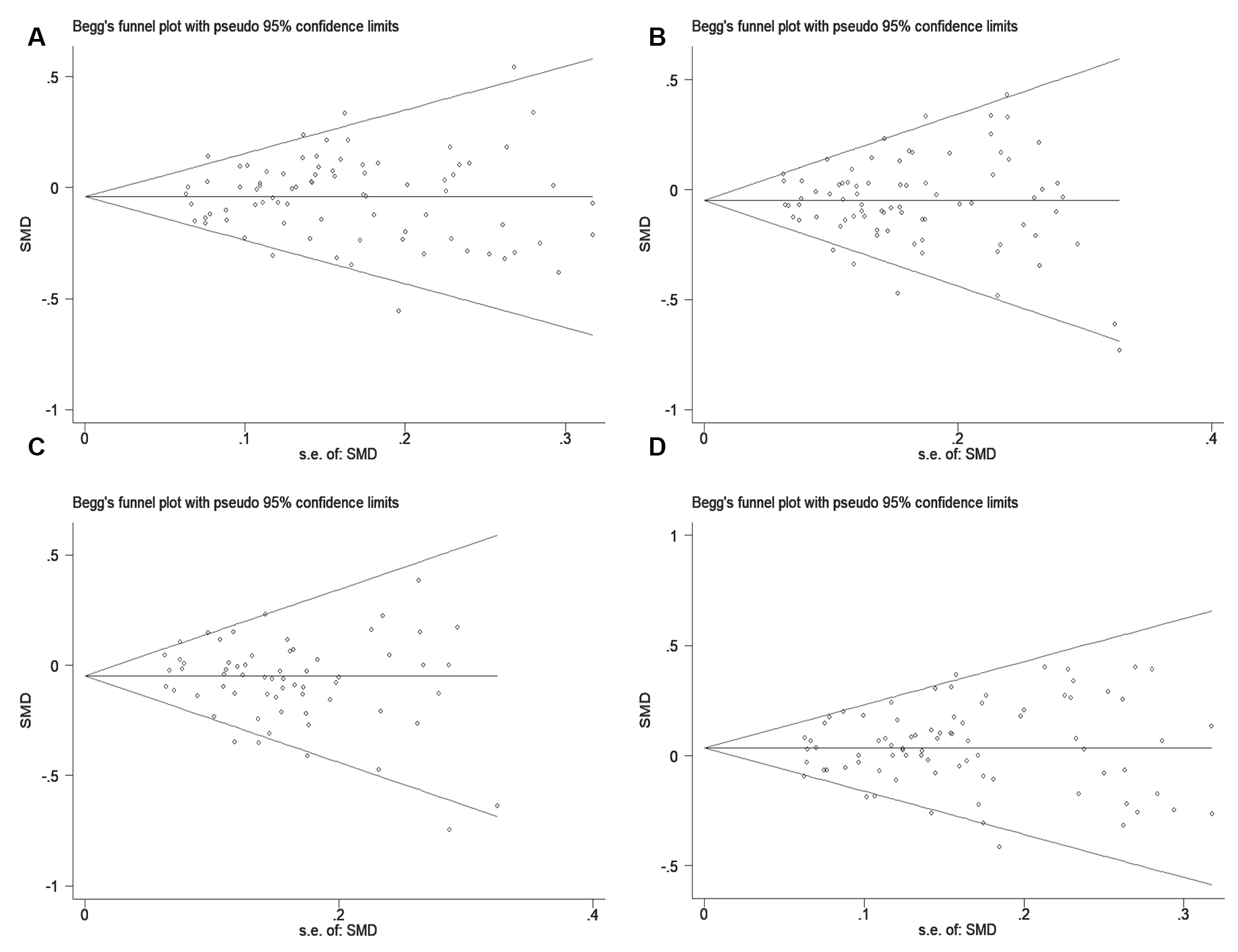


**Figure S15.** Begg’s funnel plot evaluate the publication bias of the effects of the rs1501299 polymorphism on lipid levels. Each small circle represents a separate study. The diverging lines represent 95% CI and the central line is SMD. The funnel plots should be asymmetric when there is publication bias and symmetric in the case of no publication bias [**A**: rs1501299 on TG (*P* = 0.86); **B**: rs1501299 on TC (*P* = 0.77); **C**: rs1501299 on LDL-C (*P* = 0.64); **D**: rs1501299 on HDL-C (*P* = 0.69)].


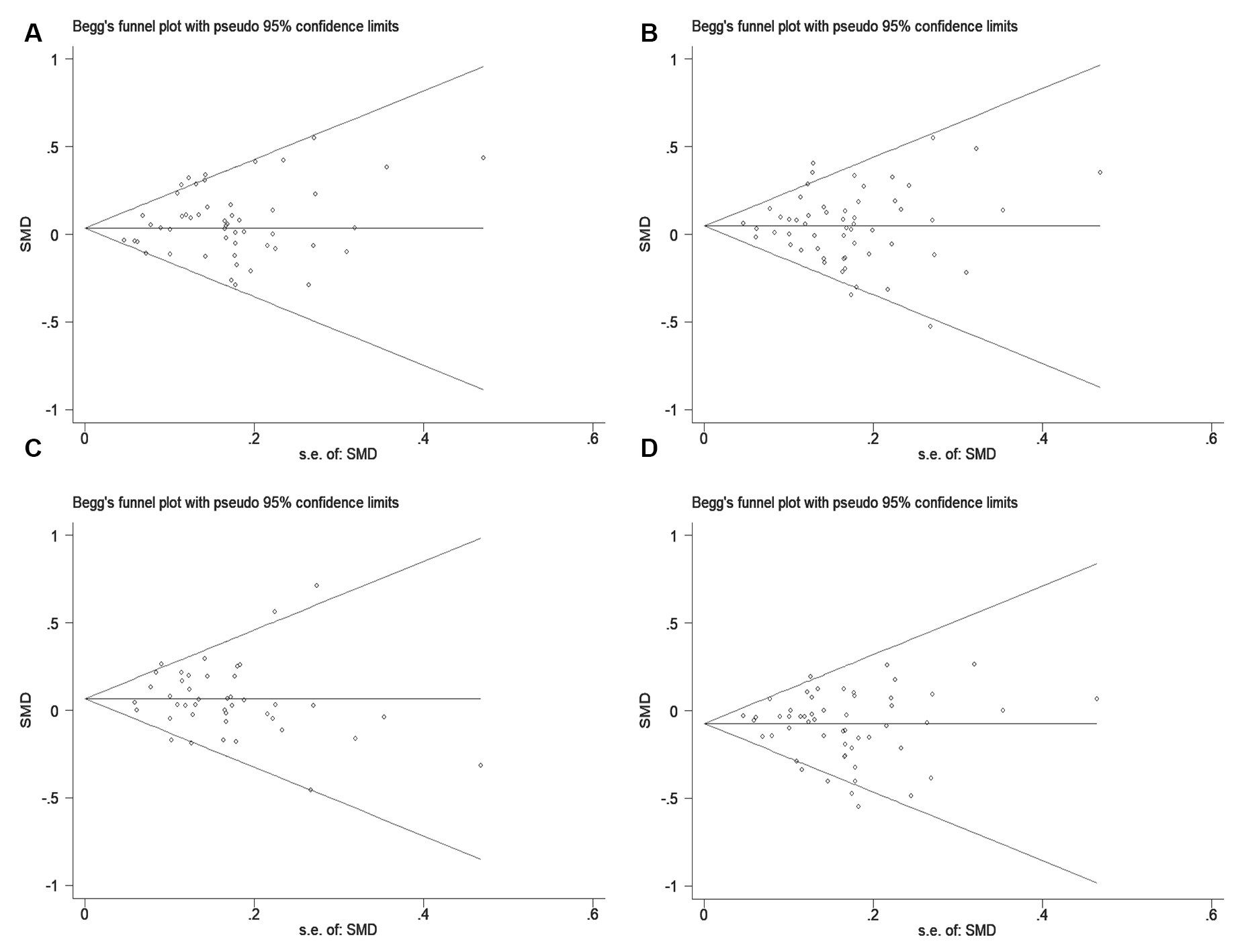


**Figure S16.** Begg’s funnel plot evaluate the publication bias of the effects of the rs266729 polymorphism on lipid levels. Each small circle represents a separate study. The diverging lines represent 95% CI and the central line is SMD. The funnel plots should be asymmetric when there is publication bias and symmetric in the case of no publication bias [**A**: rs266729 on TG (*P* = 0.97); **B**: rs266729 on TC (*P* = 0.88); **C**: rs266729 on LDL-C (*P* = 0.52); **D**: rs266729 on HDL-C (*P* = 0.43)].


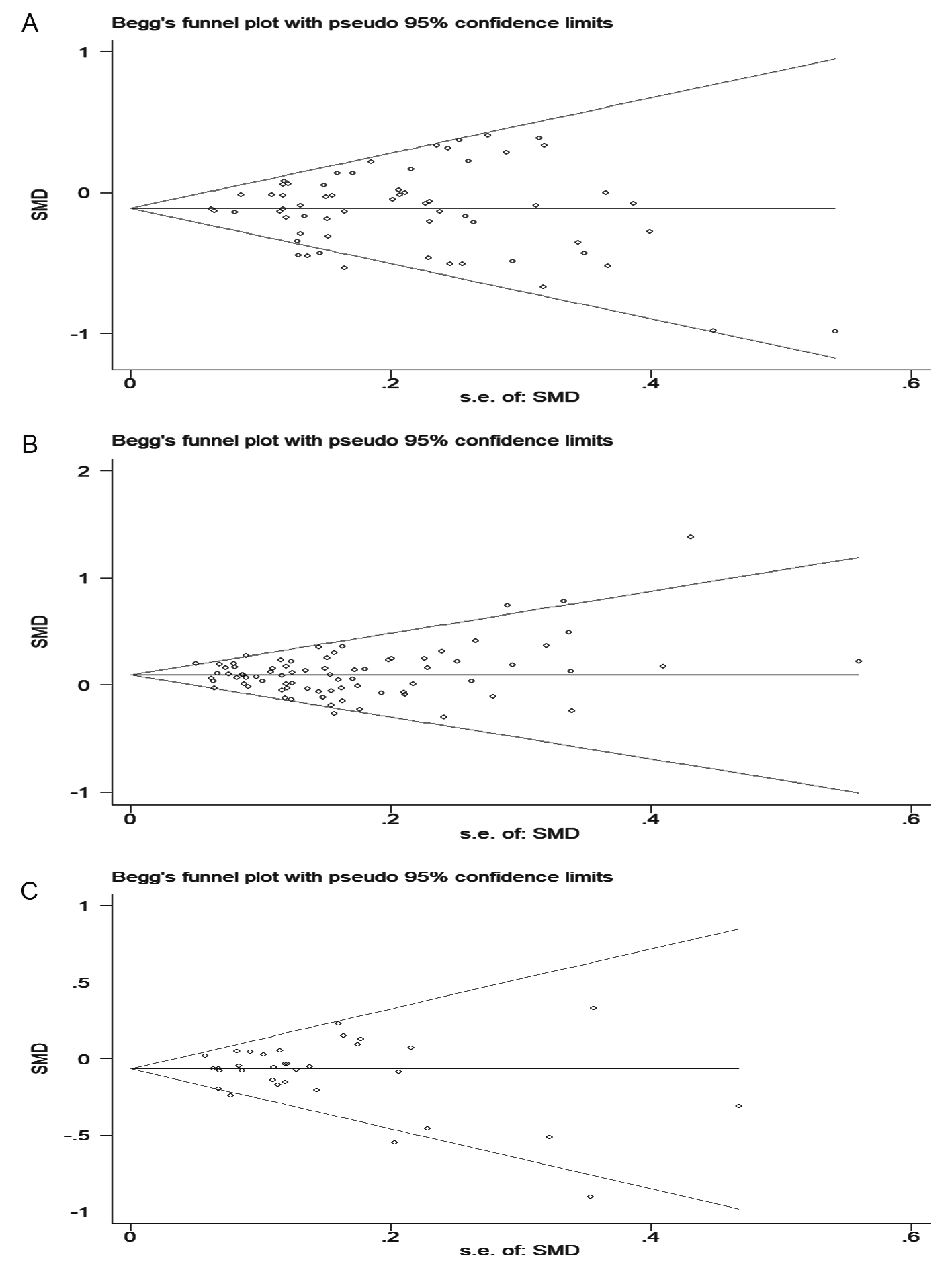


**Figure S17.** Begg’s funnel plot evaluate the publication bias of the effects of rs2241766, rs1501299 and rs266729 on circulating adiponectin levels. Each small circle represents a separate study. The diverging lines represent 95% CI and the central line is SMD. The funnel plots should be asymmetric when there is publication bias and symmetric in the case of no publication bias [**A**: rs2241766 on adiponectin levels (*P* = 0.57); **B**: rs1501299 on adiponectin levels (*P* = 0.44); **C**: rs266729 on adiponectin levels (*P* = 0.50)].
